# Transfer from spatial education to verbal reasoning and prediction of transfer from learning-related neural change

**DOI:** 10.1126/sciadv.abo3555

**Published:** 2022-08-10

**Authors:** Robert A. Cortes, Emily G. Peterson, David J. M. Kraemer, Robert A. Kolvoord, David H. Uttal, Nhi Dinh, Adam B. Weinberger, Richard J. Daker, Ian M. Lyons, Daniel Goldman, Adam E. Green

**Affiliations:** ^1^ Department of Psychology, Georgetown University, DC, USA.; ^2^ School of Education, American University, DC, USA.; ^3^ Department of Psychological and Brain Sciences, Dartmouth College, Hanover, NH, USA.; ^4^ College of Integrated Science and Engineering, James Madison University, Harrisonburg, VA, USA.; ^5^ Department of Psychology, Northwestern University, Evanston, IL, USA.; ^6^ Bloomberg School of Public Health, Johns Hopkins University, Baltimore, MD, USA.; ^7^ Penn Center for Neuroaesthetics, University of Pennsylvania, Philadelphia, PA, USA.; ^8^ Interdisciplinary Program in Neuroscience, Georgetown University, DC, USA.

## Abstract

Current debate surrounds the promise of neuroscience for education, including whether learning-related neural changes can predict learning transfer better than traditional performance-based learning assessments. Longstanding debate in philosophy and psychology concerns the proposition that spatial processes underlie seemingly nonspatial/verbal reasoning (mental model theory). If so, education that fosters spatial cognition might improve verbal reasoning. Here, in a quasi-experimental design in real-world STEM classrooms, a curriculum devised to foster spatial cognition yielded transfer to improved verbal reasoning. Further indicating a spatial basis for verbal transfer, students’ spatial cognition gains predicted and mediated their reasoning improvement. Longitudinal fMRI detected learning-related changes in neural activity, connectivity, and representational similarity in spatial cognition–implicated regions. Neural changes predicted and mediated learning transfer. Ensemble modeling demonstrated better prediction of transfer from neural change than from traditional measures (tests and grades). Results support in-school “spatial education” and suggest that neural change can inform future development of transferable curricula.

## Teaser

A spatial cognition–based curriculum in schools improved verbal reasoning, and neural changes outpredicted tests and grades.

## INTRODUCTION

The promise of neuroscience to support education remains theoretically intriguing but empirically underdeveloped (*1*–*3*). A central question is whether brain imaging can improve traditional performance-based means of assessing teaching and learning. More accurate prediction of learning transfer (i.e., predicting when learning a curriculum will generalize to untrained skill domains) would be especially valuable because transferability is often difficult to capture with traditional assessments (*4*). Other questions concern the potential of neuroscience to achieve broad impacts for education given the impracticality of large-scale neuroimaging (we cannot scan every student’s brain). This limitation may constrain the impacts of approaches intended to assess individual students’ learning or individual abilities and needs. However, research that can also support the design and improvement of curricula, assessing the curriculum rather than the student, might achieve broad impacts from relatively small-scale implementations because curricula that are developed at small/local scales can be applied broadly (*5*–*7*). Thus, determining whether neural changes that accompany curriculum learning can be leveraged to evaluate the efficacy of curricula (e.g., using neural changes to help identify curricula that impart transferable learning) has the potential for broad impacts.

A longer-standing question in philosophy and psychology concerns the extent to which spatial cognitive processes might covertly underlie seemingly verbal forms of human cognition (*8*, *9*). Mental model theory (MMT) posits that ostensibly verbal information is processed by co-opted neural resources that evolved to support visuospatial operations in primates (*8*, *10*). If this is correct, then fostering spatial processing could yield transfer to improved performance beyond the spatial domain. MMT specifically posits that the spatial process of “scanning,” inspecting spatial representations of information for key features, supports mental model–based reasoning (e.g., scanning spatial representations of verbal reasoning premises to determine whether they align to validate a logical conclusion) (*10*). This leads to the educational hypothesis that curricula designed to foster spatial cognition, especially curricula that develop spatial scanning ability, might yield transfer to cognitive abilities supported by mental modeling beyond the spatial domain, especially verbal reasoning. Behavioral and neuroimaging evidence indicates that verbal deductive reasoning often elicits spatial representations and engages spatially implicated brain regions [especially posterior parietal cortex (PPC)] (*10*–*12*). However, beyond the binary question of whether spatial resources are engaged, MMT suggests that educational improvement is likely to depend on developing the effectiveness of these resources (i.e., behavioral and neural indicators of improved spatial processing) and their integration with “executive” resources that guide reasoning [e.g., functional connectivity of PPC to dorsolateral prefrontal cortex (DLPFC) during reasoning] (*10*, *13*–*16*).

These broader questions inform a timely decision for educators and policy-makers, particularly in STEM (science, technology, engineering, and mathematics), concerning whether schools should adopt “spatial education” (i.e., classroom curricula devised to integrate visualization tools and spatial problem-solving strategies to develop spatial thinking abilities) (*17*, *18*). Individual differences in spatial ability robustly predict STEM achievement (*17*–*21*); however, implementations of curricula designed to bolster spatial ability remain rare in real-world classrooms (*17*, *18*, *21*). This scarcity of school-based spatial education has made it difficult to study outcomes. Reports from the National Research Council (NRC) (*17*), the Organisation for Economic Cooperation and Development (OECD) (*18*), and American Enterprise Institute (AEI) (*21*), indicate that, despite great theoretical promise to support STEM achievement, wider in-school adoption of spatial education is likely to depend on empirically demonstrating efficacy and mechanisms of change for spatial curricula implemented in real-world classroom settings.

Here, we investigated a curriculum devised to foster spatial cognition in real-world high school STEM classrooms and tested near- and far-transfer hypotheses. We first tested the hypotheses that the spatial curriculum would yield near transfer to improved spatial scanning and mental rotation abilities and far transfer beyond the spatial domain to verbal reasoning. To further test the hypothesis that improved spatial ability accounted for improved verbal ability (as proposed in MMT), we investigated whether the amount of improvement in spatial ability predicted the amount of improvement in verbal reasoning and whether improvement in spatial ability mediated improvement in verbal reasoning.

At the neural level, we used longitudinal functional magnetic resonance imaging (fMRI) in conjunction with the spatial curriculum with a primary goal of testing whether neural change could predict spatial-to-verbal transfer better than traditional performance-based learning assessments. Learning is fundamentally a change, so brain-based assessment of learning requires measurement at more than one time point. Longitudinal neuroimaging is frequently used to study learning in laboratory contexts but is paired with laboratory-based interventions and/or intensive training, rather than ecologically valid K-12 curricula in real-world classrooms. By contrast, in-school neuroimaging research has often used single time points of neural measurement (e.g., before or after a curriculum). Pairing longitudinal fMRI with the spatial curriculum in the present study (as an in-school learning intervention) enabled investigation of learning in terms of experimental intervention-related change effects rather than the more purely descriptive observations that are available at a single time point. We first investigated curriculum-related changes in neural activity, connectivity, and representational similarity in brain regions implicated in spatial cognition. We then investigated whether these neural changes were associated with improvements in verbal reasoning and spatial scanning and, if so, whether they mediated transfer from the curriculum to these abilities. For example, on the basis of the MMT claim that reasoning is supported by the integration of spatial neural resources with executive function resources (*10*, *13*–*16*), we tested whether improvement on verbal reasoning in the spatial curriculum was associated with increased functional connectivity of spatial cognition–implicated brain regions (including PPC) to a DLPFC region that is meta-analytically implicated in verbal reasoning. Toward our primary objective of predicting transfer from neural change, we tested whether curriculum-related neural changes during the task of spatial scanning (the spatial ability that we predicted would support mental model–based reasoning) were associated with near transfer to improved spatial scanning performance and (mirroring the behavioral analysis of spatial scanning) whether these changes also predicted improvement in verbal reasoning performance (far transfer). Note that the term prediction refers here to statistical prediction of curriculum-related change in task performance based on cognitive and brain-based measures collected for all tasks at the same two time points, as opposed to prediction of future outcomes.

Last, to compare neural change versus traditional performance-based assessment, we tested whether neural changes observed during the spatial scanning task predicted transfer to verbal reasoning over-and-above performance on the same spatial scanning task and over-and-above performance on a broader set of academic and cognitive assessments, including tests and grades.

Random assignment to in-school curricula is not possible in real-world high schools because researchers cannot assign students to classes (students choose for themselves which classes they take). Thus, to bridge from the laboratory to the classroom, we used a quasi-experimental approach that is devised to minimize selection bias in real-world contexts, including propensity scoring–based matching within schools (Supplementary Materials) (*22*, *23*). Students who received the spatial curriculum were compared to propensity scoring–matched control students at the same schools.

## RESULTS

Spatial education was studied in five public high schools in northern Virginia that adopted a spatially enriched Geoscience curriculum (*24*) in which students construct and evaluate spatial representations (maps) of real-world geographical datasets using geographic information systems (GIS) technology (Supplementary Materials; fig. S1). The course, first adopted by a group of Virginia high schools in 2005, is named “The Geospatial Semester,” although it is now implemented over two semesters. A central component of the curriculum, designed by R.A.K. and classroom educators, is to develop students’ ability to scan maps to identify key spatial features and determine how these features are related across maps (e.g., across different times or places; Supplementary Materials; fig. S1) (*24*). The curriculum is also intended to foster more frequent use of spatial thinking strategies through the use of GIS technology to visualize and analyze spatial representations between a wide range of real-world variables and draw conclusions about the relationships between these variables. In the present study, the intervention was the Geospatial course itself; that is, it was a course offered by the schools, as opposed to any sort of program or training outside the school curriculum. The structure and content of the curriculum were completely unaltered by the researchers to ensure that the study reflected an ecologically valid classroom implementation of spatial education rather than a laboratory-based intervention.

**Fig. 1.**
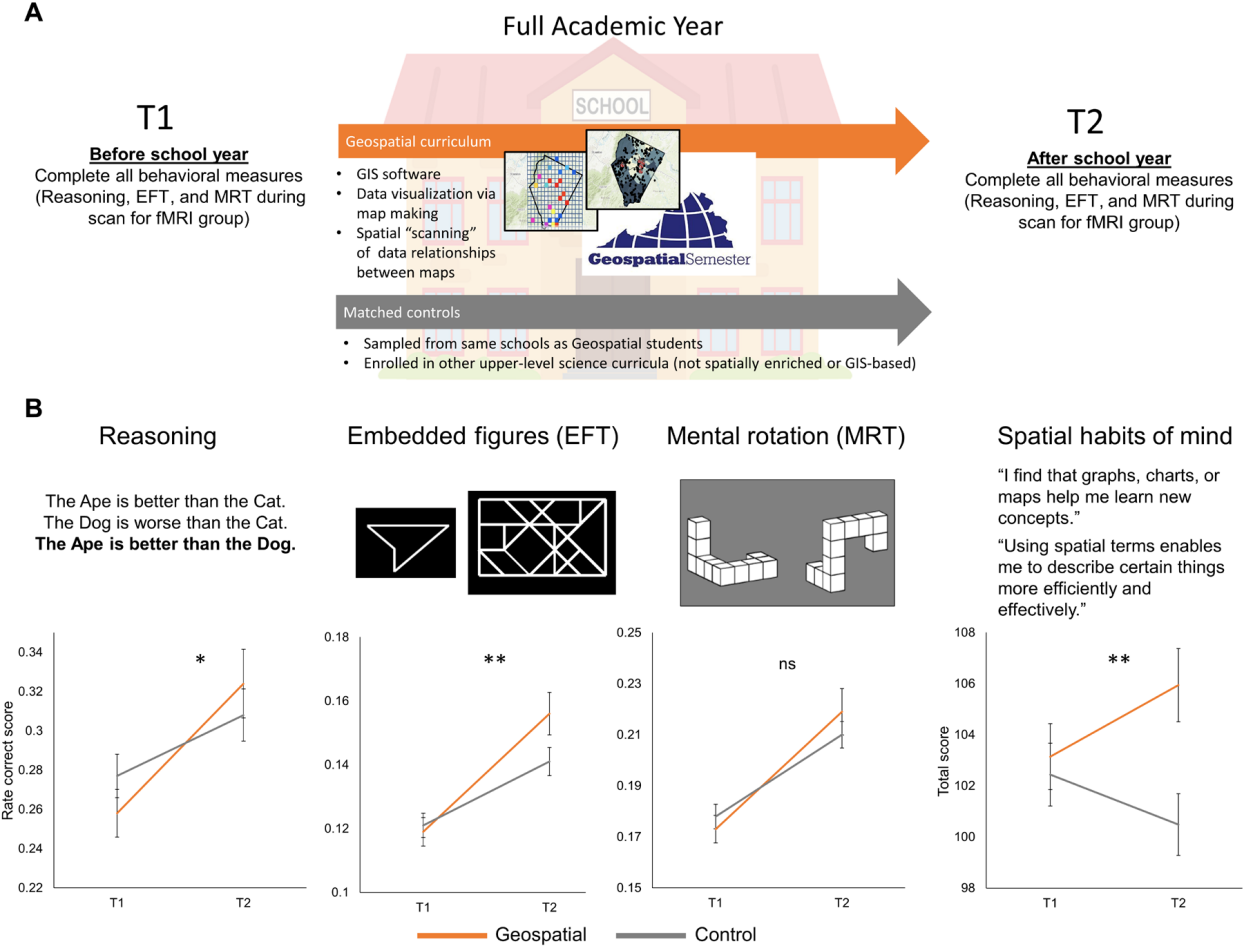
Study design and transfer results. (**A**) The longitudinal (pre-post) quasi-experimental in-school design comparing Geospatial students to matched controls at the same high schools. The paired map images representing the Geospatial curriculum (called “Geospatial Semester”) are an example GIS-based visualization of spatial data relationships, taken from a student project mapping the distribution of high-speed internet resources within a geographic region. (**B**) Example stimuli for tasks administered before and after the school year and longitudinal performance change for these tasks (*P < 0.05 and **P < 0.01, ns, not significant). Alternate versions of Reasoning, embedded figure task (EFT), and mental rotation task (MRT) were counterbalanced across T1 and T2. The spatial habits of mind inventory was administered at pre-test (before T1) and at T2.

In 346 students (206 females and 140 males; mean age = 16.61 years), propensity scoring methods were used using linear combinations of background variables including experience, interests, demographics, and academic performance (Supplementary Materials) to match Geospatial students with controls on their overall “propensity” to enroll in the Geospatial course (*22*, *23*). Propensity scoring methods reduce selection bias in real-world experimental paradigms for which randomization to groups is impossible (*22*). Participants in the main study (*N* = 182), comprising Geospatial (*n* = 77; 32 females; 16.66 years) and control (*n* = 105; 56 females; 16.63 years) groups, were tested in the summers before (T1) and after (T2) the school year on measures of verbal reasoning (*10*), spatial scanning (*25*), and mental rotation (Fig. 1) (*26*). Sixty-three participants performed these tasks during fMRI (Geospatial, *n* = 32; 13 females; 16.36 years; control, *n* = 31; 18 females; 16.60 years). The primary performance outcome was correct responses per second (Supplementary Materials) (*27*). Participants also completed a survey measure of spatial “habits of mind” (*28*) before and after the school year. Geospatial and control groups (and fMRI subgroups) did not differ significantly on any task at T1. Likewise, the final Geospatial and control groups (and fMRI subgroups) were equated on all background variables that predicted Geospatial enrollment and other demographic, academic, and spatial ability variables (table S1), indicating that propensity score matching was successful (*29*).

### Assessing spatial learning and verbal transfer

We tested whether participation in the Geospatial curriculum yielded improvement on behavioral measures via Group(Geospatial, control)-by-Time(T1, T2) analyses of variance (ANOVAs) covarying academic ability preliminary scholastic aptitude test (PSAT), scholastic performance grade point average (GPA), and gender (*30*) in the full main study sample (*N* = 182). To test the MMT-based hypothesis of transfer from spatial education to verbal reasoning, participants completed a syllogistic deductive verbal reasoning task (Reasoning) modified from the work of Ruff *et al.* (*31*). MMT research has strongly indicated that syllogistic deduction is supported by mental modeling (*32*). On each trial, participants read two premises and determined whether a subsequent conclusion was valid on the basis of the premises (Fig. 1B). Half of the trials included words that referred to overtly spatial relations (e.g., “The cow is above the pig”), and half included words that referred to overtly nonspatial relation trials (e.g., “The cow is better than the pig”). Consistent with the MMT-based prediction of verbal-domain transfer, Geospatial students showed greater Reasoning improvement than controls from T1 to T2 (Group-by-Time interaction: *F*_4,15_ = 5.60, *P* = 0.019, *np*^2^ = 0.034; Fig. 1B and table S3). Geospatial students also showed greater improvement when nonspatial relation trials (e.g., “better than”) were analyzed separately (*F*_4,158_ = 6.65, *P* = 0.011, *np*^2^ = 0.040; table S4), providing additional evidence that transfer extended beyond the spatial domain.

Two measures tested spatial learning outcomes related to the Geospatial curriculum (Fig. 1B). The embedded figure task (EFT) (*25*), taken from the work of Walter and Dassonville (*33*), was selected to assess spatial scanning because of the theorized role of spatial scanning in supporting mental modeling (*10*). The Geospatial curriculum aims to develop spatial scanning of maps (e.g., scanning the spatial features represented on a map to determine whether they align with spatial features on another map). In EFT, participants spatially scanned features of complex figures to determine whether simpler target figures aligned with figures embedded in the complex figures. Consistent with the prediction of improved spatial scanning ability in the spatial curriculum, Geospatial students improved more than controls on EFT (*F*_4,158_ = 5.38, *P* = 0.022, *np*^2^ = 0.033; Fig. 1B and table S2). Although EFT was overtly spatial and measured a skill emphasized by the Geospatial curriculum (two-dimensional spatial scanning), students were not exposed to EFT during the Geospatial course. The curriculum-driven improvement in EFT can thus be considered near transfer. Geospatial students also increased more than controls in self-reported use of spatial thinking strategies on the spatial habits of mind inventory (*F*_4,158_ = 10.27, *P* = 0.002, *np*^2^ = 0.062; Fig. 1B and table S6) (*28*). The Geospatial curriculum thus achieved two primary learning goals within the spatial domain (improved spatial scanning and increased spatial habits of mind). In addition, Geospatial course grades were generally high (78.4% A or A+), indicating strong curriculum learning (Supplementary Materials).

A third spatial measure, mental rotation task (MRT), taken from the work of Shepard and Metzler (*26*, *34*), was used to assess transfer to three-dimensional spatial rotation of objects. Participants determined whether two images of three-dimensional objects depicted rotations of the same object. Although three-dimensional rotation is not strongly related to the content of the Geospatial curriculum, which focuses on two-dimensional spatial representations (as in EFT), we hypothesized that the Geospatial course might improve spatial cognition broadly, and MRT was of interest because it is implicated in mental modeling (*8*, *10*). However, MRT performance did not show significant curriculum-specific improvement (Group-by-Time interaction: *F*_4,158_ = 1.40, *P* = 0.239, *np*^2^ = 0.009; Fig. 1B and table S5), although Geospatial students improved nominally more than controls. All behavioral Group-by-Time effects of the Geospatial curriculum remained significant (all *P* < 0.044) after experiment-wise correction for multiple comparisons (correcting for all Group-by-Time models) (*35*).

### Further testing the educational prediction of MMT

**Fig. 2.**
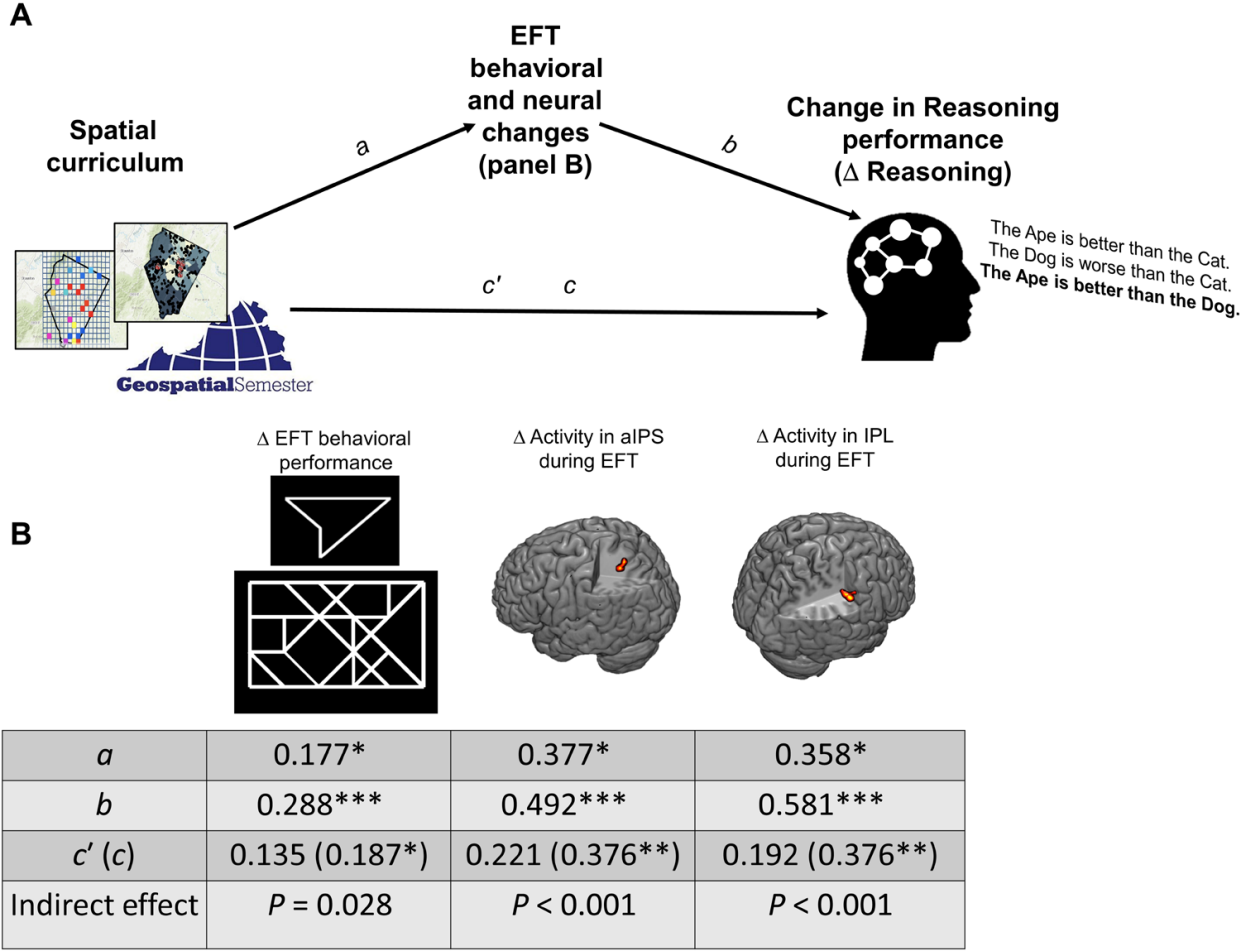
Mediation of transfer by changes in spatial scanning. (**A**) Mediation of transfer from the spatial curriculum to improved reasoning by the performance changes and neural changes on the spatial scanning task (EFT) that are shown in (**B**). The brain images in (B) display clusters in anterior intraparietal sulcus (aIPS) and inferior parietal lobule (IPL) where Geospatial students showed greater longitudinal increases in activation than controls during EFT. The table in (B) displays the path coefficients and indirect effects for models with each of the three EFT change variables as mediators. (*P < 0.05 and **P < 0.01, ns, not significant).

We next investigated the MMT-based hypothesis of spatial curriculum transfer in greater depth. If development of the spatial processes theorized to support mental modeling actually accounted for the transfer to verbal reasoning that we observed, then improvement in verbal reasoning should be related to improvement in these spatial abilities. We tested this prediction, again controlling for PSAT, GPA, and gender in all models. Consistent with MMT, longitudinal change in spatial scanning performance on EFT from T1 to T2 (ΔEFT) predicted change in Reasoning performance from T1 to T2 (ΔReasoning) for all Reasoning trials (β = 0.288, *P* < 0.001; table S11) and for the subset of nonspatial relation trials (e.g., “better”; β = 0.291, *P* < 0.001; table S12). That is, the more students’ spatial scanning improved, the more their verbal reasoning improved. Furthermore, ΔEFT mediated the transfer association of the spatial curriculum to improved reasoning for all Reasoning trials [12% of total variance explained by mediator (*36*–*38*); indirect effect: *P* = 0.028; Fig. 2] and nonspatial relation trials separately (10% of total variance; *P* = 0.036; fig. S6), further suggesting that improvement in spatial scanning may have been a cognitive mechanism by which spatial education improved verbal reasoning. In addition, supporting MMT, longitudinal change in mental rotation (ΔMRT) predicted ΔReasoning for all trials (β = 0.453, *P* < 0.001; table S13) and nonspatial trials separately (β = 0.522, *P* < 0.001; table S14). That is, the more students’ three-dimensional rotation improved, the more their verbal reasoning improved. ΔMRT was not tested as a mediator because, as noted above, it was not significantly associated with the independent Group variable (Geospatial versus control). Simultaneous regression showed that ΔEFT and ΔMRT each independently predicted ΔReasoning (for all Reasoning trials and for the subset of nonspatial relation trials; all β ≥ 0.149, all *P* ≤ 0.034; tables S15 and S16).

### Spatial curriculum–related neural changes during verbal reasoning

**Fig. 3.**
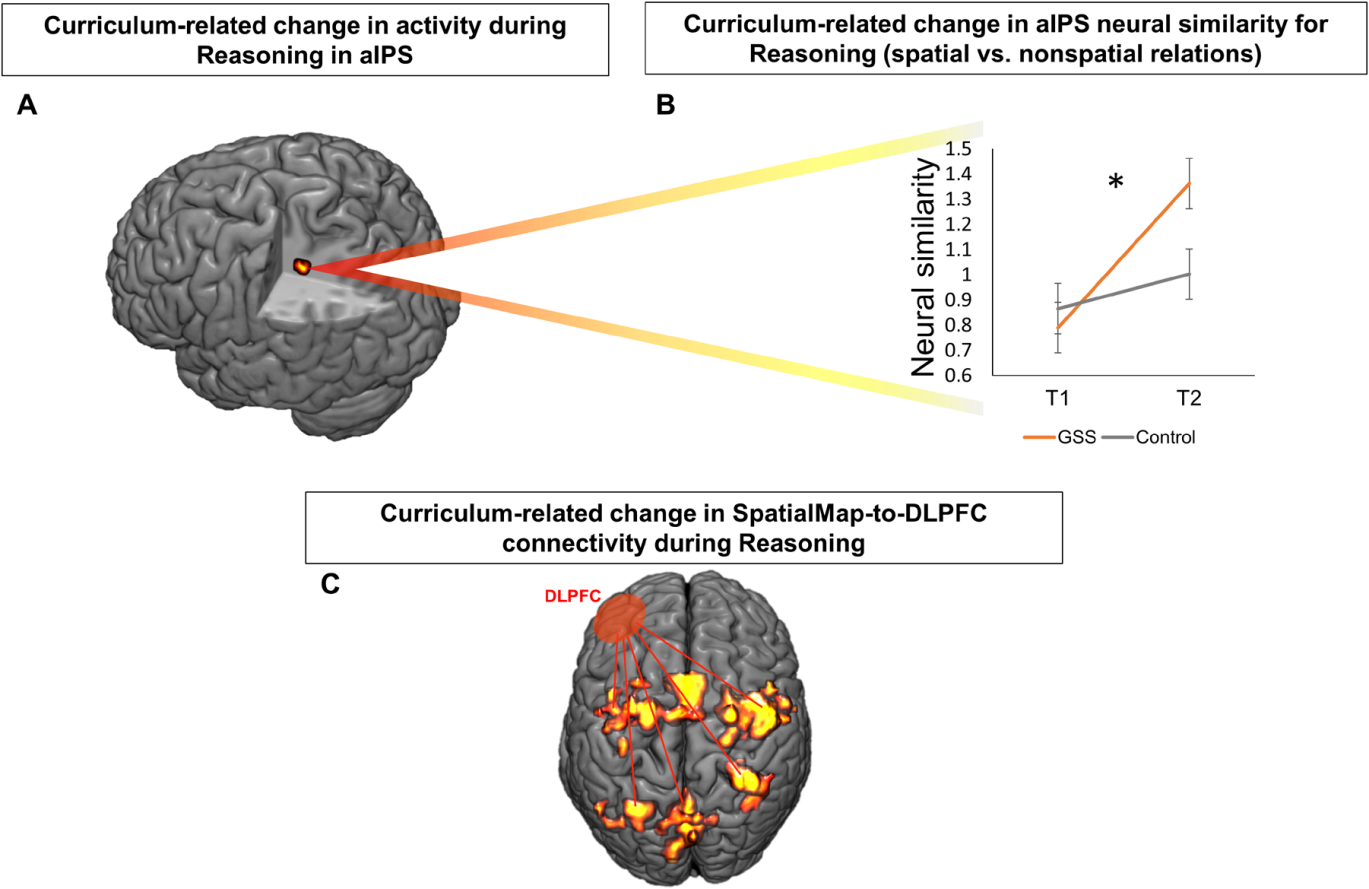
Longitudinal neural changes during Reasoning. Relative to control students, Geospatial students showed greater longitudinal increase in aIPS activity within Neurosynth-based SpatialMap (**A**), increased representational similarity between spatial and nonspatial reasoning relations in this aIPS cluster (**B**) (*P < 0.05 and **P < 0.01, ns, not significant), and increased connectivity of SpatialMap to a DLPFC region meta-analytically implicated in syllogistic deductive verbal reasoning (**C**) (*12*).

Having observed transfer from the spatial curriculum to verbal reasoning performance, we sought to relate this transfer to curriculum-driven neural changes. We first investigated whether transfer was accompanied by concurrent longitudinal changes at the neural level during the Reasoning task, using whole-brain Group(Geospatial, control)-by-Time(T1, T2) ANOVA in the 63 fMRI participants. This analysis revealed a Group-by-Time interaction, indicating longitudinal changes in activity during Reasoning that were greater for students who received the Geospatial curriculum than for control students in two regions of PPC: left anterior intraparietal sulcus (aIPS; Fig. 3A) and a cluster extending from left aIPS into left superior parietal lobule (aIPS-SPL; table S19). Because we hypothesized that the spatial curriculum would yield neural change in spatial cognition–implicated regions, we masked the whole-brain results with the meta-analytic Neurosynth (*39*) association test map for the term “spatial” (henceforth, “SpatialMap”). The more anterior IPS cluster overlapped with SpatialMap and was thus included in subsequent analyses. The aIPS-SPL cluster did not overlap with SpatialMap and was thus subsequently excluded. aIPS activity is strongly implicated in spatial cognition and especially spatial attention (*33*, *40*–*42*). No changes were greater for control than Geospatial within SpatialMap (Supplementary Materials). We additionally explored whether the effects of the spatial curriculum differed by gender, but Group-by-Time-by-Gender ANOVA indicated no three-way interaction.

MMT posits that spatial cognition resources operate in conjunction with executive function resources to support reasoning (*10*, *13*–*16*). Prior neuroimaging evidence indicates that spatial cognition–implicated brain regions interact with domain-general reasoning resources centered in DLPFC to support mental model–based reasoning (*10*, *14*). Thus, concurrent with our prediction that the spatial curriculum would improve reasoning (supported by the above behavioral findings), we predicted that the spatial curriculum would be associated with longitudinally increased functional connectivity of DLPFC to SpatialMap during Reasoning. To represent this prediction, we generated a seed region for connectivity analysis using a DLPFC region identified by a meta-analysis of neuroimaging studies of verbal syllogistic deductive reasoning (the form of reasoning studied here) (*12*). Whole-brain Group-by-Time ANOVA for psychophysiological interaction (PPI)–based functional connectivity to the DLPFC seed region was then masked with SpatialMap. Consistent with the MMT-based prediction, this analysis revealed that Geospatial students increased more than controls in connectivity of SpatialMap regions to DLPFC (Fig. 3C, fig. S10, and table S23). No connectivity changes were greater for control students than Geospatial students. A Group-by-Time-by-Gender interaction emerged whereby female Geospatial students showed greater curriculum-related increase in connectivity to parietal and premotor regions than males (fig. S11 and table S24), suggesting that this connectivity increase was especially strongly associated with spatial curriculum–based reasoning improvement in female students.

A pedagogical goal of spatial education is to encourage students to “spatialize” the information that they encounter (i.e., developing spatial representations of information even when the information is presented in nonspatial modalities) (*20*, *24*). As noted above, the Reasoning task was devised such that half the trials included overtly spatial relations (e.g., “above”) and the other half included overtly nonspatial relations (e.g., better). A primary reason for this was to enable us to directly test the prediction that the Geospatial curriculum was associated with “spatialized” representation of overtly nonspatial relations. We operationalized this prediction using representational similarity analysis (RSA; Supplementary Materials). Specifically, we tested whether the neural patterns associated with overtly nonspatial relation trials became more similar to the patterns associated with overtly spatial relation trials in the SpatialMap cluster that showed spatial curriculum–related change in activity during Reasoning (aIPS; Fig. 3A) and whether such spatialization was greater for Geospatial relative to control students. Consistent with these predictions, Geospatial students showed increased similarity between nonspatial and spatial trials in aIPS (paired *t* test for T1 versus T2 similarity in Geospatial students: *t* = 3.43, *P* = 0.001, *d* = 0.83; Supplementary Materials). Further analysis of aIPS patterns indicated that the change in nonspatial-to-spatial similarity was primarily driven by changes in the nonspatial trials (i.e., nonspatial trials becoming more similar to spatial trials). That is, nonspatial relation trials at T1 were significantly less similar to nonspatial relation trials at T2 than spatial relation trials at T1 were to spatial relation trials at T2 (*t* = 2.44, *P* = 0.018, *d* = 0.80). Group-by-Time ANOVA showed that this increase in nonspatial-to-spatial similarity in aIPS was greater in Geospatial students than in controls (*F*_2,61_ = 4.71, *P* = 0.034, *np*^2^ = 0.072; Fig. 3B and table S25).

To further investigate potential neural mechanisms of change associated with the effect of the spatial curriculum on verbal reasoning, we tested whether each curriculum-related longitudinal neural change that we observed for Reasoning was associated with change in Reasoning performance (ΔReasoning). All models again controlled for PSAT, GPA, and gender. Local change in Reasoning activity in aIPS was not strongly associated with change in Reasoning performance (β = 0.152, *P* = 0.238; table S31). RSA-based nonspatial-to-spatial similarity increase within the aIPS cluster showed a medium effect on ΔReasoning (β = 0.242), although this association was marginally nonsignficant (*P* = 0.062; table S32). Increased connectivity of SpatialMap to DLPFC showed a medium effect on ΔReasoning (β *=* 0.309), and this association was significant (*P* = 0.016; table S30). That is, the more SpatialMap-to-DLPFC connectivity during Reasoning increased longitudinally, the more students’ reasoning improved. Subsequent mediation analyses indicated a medium indirect effect of the Geospatial curriculum on ΔReasoning (14% of total variance) (*36*–*38*), although this was not significant (indirect effect, *P* = 0.088).

### Spatial curriculum–related neural changes during spatial scanning (EFT)

As described above, the spatial curriculum was associated with improvement in spatial scanning performance on EFT. As above, our first pass at the imaging data was to investigate whether this improvement was accompanied by concurrent curriculum-driven longitudinal changes in neural activity for EFT. Whole-brain Group(Geospatial, control)-by-Time(T1, T2) ANOVA controlling for PSAT, GPA, and gender in the 63 fMRI participants identified longitudinal changes that were greater in Geospatial students than controls in two PPC regions (Fig. 2): left aIPS (proximate to, but not overlapping, the change in aIPS during Reasoning) and right inferior parietal lobule (IPL), as well as regions in middle frontal gyrus, frontal pole, and premotor cortex (fig. S7 and table S18). Because EFT is a spatial cognition task, we did not mask the results with SpatialMap to identify regions implicated in spatial cognition. While all of the regions where longitudinal changes were observed for EFT have previously been associated with spatial function, this evidence is strongest for the PPC regions (*40*, *43*–*45*). No changes in activity were greater for control students than Geospatial students, and Group-by-Time-by-Gender ANOVA indicated no three-way interaction. No connectivity or RSA analyses were conducted for EFT because there were no relevant hypotheses.

Paralleling the Reasoning task analyses above, we next tested whether each of these curriculum-related changes for EFT neural activity was associated with change in spatial scanning performance (ΔEFT), again controlling for PSAT, GPA, and gender. Longitudinal change in each of the observed PPC regions, i.e., change in aIPS activity (ΔaIPS_EFT) and change in IPL activity (ΔIPL_EFT), was strongly associated with ΔEFT (all β ≥ 0.424, all *P* < 0.001; tables S26 and S27). That is, the more that activity in each of these PPC regions increased longitudinally, the more students improved in spatial scanning performance. The activity changes observed in frontal and premotor regions were not strongly associated with ΔEFT (All β ≤ 0.201, all *P* ≥ 0.091). IPL and aIPS (as noted above) have been consistently implicated in spatial attention (*40*, *43*–*45*), including the attentional demands of spatial scanning in EFT (*33*). Notably, these regions also appear to support attentional demands of ostensibly nonspatial tasks (e.g., mental modeling–associated verbal reasoning, numerical cognition, and nonspatial working memory) (*33*, *41*, *42*, *46*).

fMRI data for the other spatial ability measure that we administered (i.e., MRT) were collected, but analyses of those data were not included among the primary analyses that sought to identify neural correlates of spatial curriculum–related change in performance because, as described above, MRT performance was not significantly related to the Geospatial curriculum. fMRI analyses for MRT are reported in the Supplementary Materials (fig. S9 and tables S21 and S22).

### Does neural change predict transfer?

A primary objective of this research was to test whether neural change predicted learning transfer and, if so, whether neural change offered predictive value over-and-above traditional performance-based assessments. We specifically sought to test the brain-based hypothesis of MMT that development of spatial resources, especially resources associated with spatial scanning, predicted improved verbal reasoning. We thus investigated whether neural changes on the spatial scanning task (EFT) predicted transfer to Reasoning. All models again controlled for PSAT, GPA, and gender. Neural change on Reasoning was not considered in these analyses because the goal was to identify neural changes that could predict transfer to Reasoning. If the transfer task itself (Reasoning) is required to obtain a neural measure of transferable learning, then it is not clear that a meaningfully predictive inference can be made. Consistent with MMT, both of the spatial curriculum–related longitudinal changes in PPC activity identified for EFT (ΔaIPS_EFT and ΔIPL_EFT) were strongly associated with change in Reasoning performance (ΔReasoning; All β ≥ 0.492, all *P* < 0.001; tables S28 to S29). That is, the more that spatial scanning–related activity in aIPS and IPL increased, the more students improved on verbal reasoning. Recall that, consistent with MMT, improved spatial scanning performance (ΔEFT) mediated the transfer association of the Geospatial curriculum to improved reasoning. We thus sought to mirror this analysis at the neural level, testing ΔaIPS_EFT and ΔIPL_EFT as neural mediators of transfer. Consistent with the behavioral findings, ΔaIPS_EFT and ΔIPL_EFT each mediated the transfer association of the Geospatial curriculum to ΔReasoning (ΔaIPS_EFT: 41% of total variance, *P* < 0.001; ΔIPL_EFT: 49% of total variance, *P* < 0.001; Fig. 2). The changes in EFT activity in prefrontal and premotor regions that were not associated with ΔEFT (above) were also not associated with ΔReasoning (All β ≤ 0.124, all *P* ≥ 0.312).

### Does neural change outpredict traditional performance-based assessments?

Having identified two curriculum-related changes in neural activity during spatial scanning (EFT) that predicted transfer to verbal reasoning, we next sought to compare these neural changes (ΔaIPS_EFT and ΔIPL_EFT) to performance-based assessments. To enable a direct comparison of the predictive value derived from neural change versus performance-based learning assessment, we compared EFT neural change versus performance-based learning on the same EFT task (ΔEFT). ΔEFT represented curriculum learning (specifically, the curriculum-related improvement in spatial scanning). As described above, ΔEFT was also highly predictive of improvement in the Reasoning transfer task (ΔReasoning), which was the outcome variable that we sought to predict in the current analysis. Thus, ΔEFT represented a strong performance-based predictor against which to compare neural change variables. Performance change on another curriculum-related learning outcome (spatial habits of mind) (*24*, *28*) was also included as a predictor of transfer, as were change in MRT performance (ΔMRT), traditional academic assessments (PSAT and GPA), and gender. In addition, Geospatial course grades were included for models considering Geospatial students only. Including these multiple performance-based measures, in addition to ΔEFT, was intended to further increase the rigor of the comparison to neural change and to reflect that the standard for demonstrating the value of neuroimaging predictors should be comparison to multiple performance-based tasks (not just one task) because of the greater pragmatic burden required for obtaining neuroimaging measures.

Multiple regression models (Supplementary Materials) found the following: (i) The two neural change predictor variables (ΔaIPS_EFT and ΔIPL_EFT) each individually yielded significantly better model fit for predicting ΔReasoning than any of the performance-based assessments in the full neuroimaging sample and in the sample of Geospatial students only [ΔEFT, ΔMRT, change in spatial habits of mind, PSAT, GPA, Geospatial course grade (Geospatial students only), and gender; likelihood ratio tests: all *P* < 0.001]. (ii) The combination of ΔaIPS_EFT and ΔIPL_EFT yielded significantly better fit than the best-fitting combinations of performance-based assessments (in the full neuroimaging sample: ΔEFT, ΔMRT, and PSAT; in Geospatial students only: ΔEFT, ΔMRT, and Geospatial course grades; all *P* ≤ 0.007; tables S45 and S46). (iii) Adding ΔaIPS_EFT and ΔIPL_EFT to the best-fitting combination of performance-based assessments in the full and Geospatial-only neuroimaging samples significantly improved fit (all *P ≤* 0.043; table S49 and S52), and adding either ΔaIPS_EFT or ΔIPL_EFT individually improved fit for the full sample (all *P* ≤ 0.002; tables S47 and S48) and for the Geospatial-only sample in the case of ΔaIPS_EFT (*P* = 0.021; table S50) but not ΔIPL_EFT (*P* = 0.066; table S50). Notably, although the neural change variables were the strongest individual predictors, the strongest fit among all models was achieved by a model combining the two neural change variables (ΔaIPS_EFT and ΔIPL_EFT) with three performance-based assessments (ΔEFT, ΔMRT, and PSAT; adjusted *R*^2^ = 0.39; table S53).

We further used data-driven ensemble modeling prediction analysis (Supplementary Materials) (*47*) to identify the predictor variables that are most likely, in general, to be predictive of the outcome variable (in this case, ΔReasoning); that is, the variables that contribute the most to prediction across the possible models represented by the ensemble rather than within an individual model. We first used an ensemble model “boosting” analysis intended to optimize model accuracy (Supplementary Materials) (*46*, *47*), again including ΔaIPS_EFT and ΔIPL_EFT as the neural predictors, along with ΔEFT and the same set of additional performance-based predictors [ΔEFT, ΔMRT, change in spatial habits of mind, PSAT, GPA, Geospatial course grade (Geospatial students only), and gender]. The prediction analysis identified the two neural change predictor variables as the variables that contributed most to prediction of ΔReasoning (table S33): ΔIPL_EFT (accounting for 38% of predictor “importance”) and ΔaIPS_EFT (accounting for 28%). Of the performance-based assessment variables, ΔEFT was assigned the highest predictor importance (16%) across the ensemble model. When Geospatial course grade was included as a predictor (Geospatial students only), the model again indicated ΔaIPS_EFT (26%) and ΔIPL_EFT (24%) as the most important predictors, with ΔEFT next at 18% (table S35). Additional ensemble modeling analyses to build “standard” prediction models (tables S33 to S44) largely converged with the boosting analyses. That is, data-driven variable selection resulted in the inclusion of neural change predictors in the most informative model (Akaike information criterion). Specifically, three variables were selected for inclusion in the standard prediction model: ΔIPL_EFT, PSAT, and ΔaIPS_EFT (Supplementary Materials). Iterating this analysis with the Geospatial course grades included (Geospatial students only) resulted in the selection of two variables: ΔaIPS_EFT and PSAT.

Last, post hoc exploratory analyses investigated whether the longitudinal neural changes that were associated with improved Reasoning performance were also associated with each other. As described above, the change in SpatialMap-to-DLPFC connectivity was the only longitudinal neural change during the Reasoning task that was significantly associated with improved reasoning performance. As also described above, changes in two PPC regions (ΔaIPS_EFT and ΔIPL_EFT) predicted improved reasoning performance. We thus sought to test whether ΔaIPS_EFT and ΔIPL_EFT were related to change in SpatialMap-to-DLPFC connectivity during Reasoning. The ΔaIPS_EFT and ΔIPL_EFT clusters spatially overlapped (in IPS and IPL) with areas of increased SpatialMap-to-DLPFC connectivity during Reasoning, and both ΔaIPS_EFT (β = 0.414, *P* < 0.001; table S55) and ΔIPL_EFT (β = 0.262, *P* = 0.038; table S56) predicted the increase in SpatialMap-to-DLPFC connectivity. This post hoc finding provides an additional indication that spatial education may influence IPS and IPL function in ways that support both spatial scanning and verbal reasoning (putatively because the same spatial attentional resources that support spatial scanning also contribute to verbal reasoning, including via communication with DLPFC). Relatedly, as well as post hoc, we tested whether the longitudinal changes in aIPS activity that we observed separately in EFT and Reasoning were associated with each other and found that they were (β = 0.340, *P* = 0.006; table S57).

## DISCUSSION

Spatially based accounts of human cognition (e.g., MMT) (*9*, *48*) suggest the educational hypothesis that fostering improvement in spatial cognitive processes might improve abilities beyond the spatial domain, including verbal reasoning. Consistent with this prediction, a spatial STEM curriculum in the present study transferred to an untrained verbal reasoning task that is strongly linked to mental modeling. Furthermore, the more students improved on spatial scanning and mental rotation, abilities that are specifically theorized to support mental modeling (*10*), the more they improved on verbal reasoning, and improvement on spatial scanning mediated the association of the spatial curriculum to improved verbal reasoning. Likewise, at the neural level, longitudinal fMRI detected spatial curriculum–related increases in the activity of spatial cognition–implicated brain regions (aIPS and IPL) during spatial scanning (EFT). These neural changes were associated with improvement in spatial scanning performance and mediated the association of the spatial curriculum to verbal reasoning. These findings extend evidence for mental modeling as a basis of human reasoning (*8*) by experimentally intervening to improve spatial ability as a means of improving verbal ability and indicate that MMT can be translated to real-world STEM classrooms via spatial education. The present evidence suggests further exploration of spatial scanning in particular as a trainable ability to support reasoning in educational and other contexts. Although we did not find curriculum-specific effects on MRT, mental modeling involves multiple aspects of spatial cognition (including both spatial scanning and mental rotation) (*8*), and it is possible that a curriculum focused on three-dimensional objects and rotation might have produced greater improvements in MRT performance.

Addressing research priorities noted in the NRC, OECD, and AEI reports (*17*, *18*, *21*), the present work provides evidence for the real-world efficacy of an in-school implementation of spatial education and empirically indicates plausible cognitive and neural mechanisms of change. With respect to efficacy, improved spatial scanning, spatial habits of mind (another outcome strongly linked to STEM achievement) (*28*), and verbal reasoning represent spatial curriculum–driven gains both within and beyond the spatial domain. This evidence was strengthened by the use of propensity scoring methods to reduce selection bias. Prior research has demonstrated transfer effects from implementations of spatial training (*49*–*52*); however, these studies have assessed transfer to measures of nonverbal cognition, including spatial tasks, such as mental rotation (*49*, *50*), and mathematics, such as numerical processing and geometry (*51*, *52*). That work is thus distinct from the present evidence of transfer from the spatial curriculum to verbal reasoning. Regarding mechanisms of change, the above-noted findings implicate curriculum-related cognitive and neural changes in spatial scanning as plausible mechanisms. Additional evidence indicated that SpatialMap connectivity to a reasoning-associated DLPFC region increased longitudinally in Geospatial students relative to controls. The amount of increase in this connectivity predicted the amount of improvement in reasoning. These findings point to increased SpatialMap-to-DLPFC communication as a neural change that may enable better reasoning, putatively because this communication facilitates greater contribution of spatial resources to reasoning (e.g., spatial attentional resources linked to IPL and IPS). Female students showed greater curriculum-driven increases in connectivity than males, suggesting that this connectivity may be especially important for the effects of spatial education in girls. This is noteworthy with respect to previous evidence indicating the potential of spatial education to support female students’ participation and achievement in STEM (*53*), particularly given the relationship of both spatial abilities and reasoning to STEM achievement (*19*). In addition, evidence that the spatial curriculum was associated with changes in activity and representational similarity in IPS during reasoning, although these changes were not associated with task performance, further suggests that spatial education influences the function of IPS, which is associated with diverse spatial cognition–related abilities (*33*, *40*–*42*). In the context of growing evidence and enthusiasm for the importance of spatial cognition among education researchers, especially in STEM (*17*, *18*, *48*), which has not yet permeated education policy and practice (*17*, *18*, *21*), the classroom-based evidence obtained in the present study provides new empirical support for the adoption of spatial education.

Interpretation of the present results with respect to transfer to reasoning in real-world contexts is constrained by the use of a traditional laboratory test of verbal reasoning as our reasoning transfer measure (rather than an assessment of real-world reasoning), although syllogistic verbal reasoning of the kind studied here has been shown to predict real-world academic and professional achievement across a variety of domains (e.g., reading, chemistry, nursing, and medicine) (*54*). Note that, while all Reasoning trials were verbal, only half (20 trials) involved overtly nonspatial relations (e.g., better), limiting the number of trials upon which the most direct evidence regarding transfer beyond the spatial domain is based.

The present research shows proof of principle for using in-school neural change to predict learning transfer more accurately than traditional performance-based assessments. We predicted improvement on the Reasoning transfer task from neural change on a separate task (EFT; reflecting the MMT-based hypothesis that spatial scanning supports mental model reasoning) (*10*). Direct model comparisons and ensemble modeling prediction analysis showed that EFT neural changes in IPL and IPS were stronger predictors of transfer than performance-based academic and cognitive assessments (even when comparing neural versus performance change on the same EFT task). Notably, however, the best prediction of learning transfer was achieved by combining neural changes with traditional assessments. These findings indicate that neural change has the capacity to improve assessment of the transferability of curricula and suggest that the most effective approaches may use measures of neural change to bolster (but not replace) traditional assessments.

Assessing the transferability of curricula used for classroom teaching can be accomplished on relatively small scales, including the small-scale development of the Geospatial curriculum by researchers and high school educators in Virginia (*24*). Nonetheless, the curricula that emerge from these assessment efforts can have broad impacts (*5*–*7*). Leveraging neural changes that predict transferability to enable neurally informed curriculum assessment thus has long-term promise as a means by which neuroscience may inform education by informing curriculum design (e.g., by identifying existing curricula that are likely to achieve transfer, identifying changes to curricula that can improve transfer, and informing development of novel transferable curricula) while avoiding the practical and ethical limitations of large-scale neuroimaging (i.e., by assessing curricula rather than individual students). At a more basic implementation level, fMRI detection of curriculum-specific, in-school longitudinal changes in neural activity, connectivity, and representational similarity in the present study supports the efficacy of longitudinal fMRI to study learning of specific curricula in real-world K-12 classroom environments. This suggests further potential for longitudinal fMRI to help address the gap between real-world school settings, where learning is perhaps most consequential, and laboratory settings, where longitudinal neural measurement is regularly applied to specific in-laboratory curricula/regimens. Insightful work on first grade attendance/exposure and an intensive Law School Admission Test (LSAT) preparation course in college classrooms (*55*) and neuroimaging during lectures viewed remotely by college students outside the classroom (*56*) have previously suggested the potential efficacy of longitudinal fMRI, although they have not sought to measure curriculum-specific neural change in real-world K-12 classroom environments.

An important limitation/caveat to consider in such work, including in the current study, is that, when neural change is accompanied by change in behavioral performance, it may be difficult to fully rule out effects of factors that influence performance (e.g., difficulty/effortfulness) as opposed to the hypothesized changes in neural recruitment that reflect curriculum learning. While we cannot conclusively rule out such potentially confounding influences, these influences do not appear likely to account for the differences in activity that are the focus of the current study. In particular, performance improved from T1 to T2 in Geospatial students (suggesting less difficulty at T2), yet activity increased in specific parietal regions heavily associated with spatial cognition. By contrast and speaking in very broad terms, decreased difficulty is generally associated with decreased activation (*57*, *58*), and activation did show more global decreases from T1 to T2 (Supplementary Materials). In addition, the regions that we focused on do not appear to be strongly reflective of task difficulty in general (*57*–*60*). Furthermore and in our view, the MRI analyses of greatest interest in the present study (i.e., analyses leveraging neural data to predict transfer) appear to be less susceptible to potential interpretive confounds related to task performance. In particular, we found that EFT neuroimaging outcomes (e.g., changes in activation in aIPS and IPL during EFT) predicted curriculum-related far transfer to performance on a separate transfer task (Reasoning) even when controlling for EFT behavioral performance (i.e., controlling for the performance that occurred for this task in the scanner while the MRI data were being collected). The differences in MRI data from T1 to T2 during EFT provided much stronger prediction of transfer to Reasoning than the behavioral data on the EFT task in both the conventional regression models and the ensemble modeling prediction analyses. These results suggest that the differences that we observed in imaging data are meaningfully distinct from the behavioral performance and are unlikely to be simply attributable to differences in performance.

While neural change–based prediction of learning transfer suggests future educational applications, determining how broad or specific such applications might be will require investigating the breadth of transferability that neural changes, especially in spatial cognition–related regions, can predict. Whereas this proof-of-principle study focused on spatial scanning in EFT (near transfer) and more distant transfer to verbal Reasoning, it seems unlikely that the curriculum-related neural changes observed here would uniquely influence these two tasks. Testing wider arrays of transfer tasks to determine the extent to which neural changes reflect development of generalizable underlying abilities (i.e., abilities that are not task-specific) can determine the more global value of neuroimaging-based transfer prediction. Neural changes in spatial cognition–implicated brain regions are plausible candidates to predict broad transferability. Transfer from spatial training to math has been recently observed (*61*), and spatial resources, including spatial attentional resources associated with IPL and IPS. appear to support diverse forms of cognition (e.g., planning, creativity, numeracy, causal understanding, linguistic representation, and physics concept knowledge), many of which are linked to mental modeling (*8*, *46*, *62*–*64*).

## MATERIALS AND METHODS

### The Geospatial course

The Geospatial course (*24*) was created as a partnership between public high schools in Virginia and the Integrated Science and Technology Department at James Madison University (JMU). Students take the course for either 45 min daily or 90 min every other day and receive college credit from JMU. JMU faculty members mentor the high school teachers, providing technical support, teaching and observing classes, and assisting in mentoring students on their course projects. The Geospatial Course curriculum is designed to enhance spatial thinking skills, including spatial scanning, and foster increased use of spatial thinking strategies through the use of geospatial technology such as GIS (e.g., ArcGIS; www.arcgis.com). More information about the Geospatial Course is provided at www.isat.jmu.edu/geospatialsemester/. This study (protocol 2014-0725; title: “Cognitive and neural indicators of school-based improvements in spatial problem solving”) was approved by the Georgetown University Institutional Review Board.

### Behavioral tasks

#### Reasoning

On each Reasoning trial [see fig. S2 and see the work of Ruff *et al.* (*31*)], participants indicated by keypress (“yes”/“no”) whether a conclusion sentence followed logically from two preceding premise sentences. Of 60 total trials, 40 were reasoning problems, 20 involving spatial relations (e.g., above/“below”) and 20 involving nonspatial relations (e.g., better/“worse”). The remaining 20 trials were matching problems, which required participants to determine whether the conclusion exactly matched either of the two premises. Following Ruff *et al.* (*31*), these matching items served as a control condition for neuroimaging analyses and were not included in behavioral performance analysis. For each trial type, half were true and half were false. Once the conclusion sentence appeared, participants had up to 8 s to respond (Supplementary Materials; fig. S2 displays complete timing). Behavioral performance scores for the Reasoning task (and all behavioral tasks) were computed as the number of correct responses per second (rate correct score; Supplementary Materials) (*65*). Table S8 displays descriptive statistics for Reasoning. Results of Group(Geospatial, control)-by-Time(T1, T2) ANOVA are shown in table S3.

#### Embedded figure task

On each EFT trial [fig. S3; see the work of Walter and Dassonville (*33*)], participants were given up to 10 s to respond by keypress (yes/no) to indicate whether a simple figure could be found within a more complex figure. There were 30 total trials (20 true and 10 false). Task trials were compared to baseline fixation in the fMRI analyses, as in previous implementations (*66*, *67*). Descriptive statistics for EFT are shown in table S7. Results of Group(Geospatial, control)-by-Time(T1, T2) ANOVA are shown in table S2.

#### Mental rotation task

On each MRT trial [fig. S4; see the work of Shepard and Metzler (*26*, *34*)], participants were given up to 7 s to indicate by keypress (“Yes”/“No”) whether two images depicted rotations of the same three-dimensional object. Of 84 total trials, each of three different angles of rotation (50°, 100°, and 150°) was used 24 times. Following the work of Voyer and Hou (*68*), we used a 2:1 ratio of true (same object) to false (different objects) trials across all trial types. Consistent with prior work (*45*), fMRI analysis contrasted true trials versus control trials (0° of rotation). Descriptive statistics for MRT are shown in table S9. Results of Group(Geospatial, control)-by-Time(T1, T2) ANOVA are shown in table S5.

#### Spatial habits of mind inventory

The spatial habits of mind inventory (*28*) is a 28-item self-report survey assessing the extent to which individuals engage in essential dimensions of spatial thinking strategy use, including pattern recognition, spatial description, visualization, and spatial concept use. Participants rate (1 to 5) how well statements about spatial thinking strategy use apply to them, and a total sum score is calculated (highest possible score = 140). While other behavioral measures were administered at T1 and T2, spatial habits of mind inventory was administered at pretest and T2. Descriptive statistics for spatial habits of mind inventory are shown in table S10. Results of Group(Geospatial, control)-by-Time(T1, T2) ANOVA are shown in table S6.

### Statistical analysis

All statistical analyses were computed in RStudio (*69*) and SPSS version 27 (*70*). Ensemble modeling prediction analyses were computed via Automatic Linear Modeling in SPSS 27 (*47*). Hierarchical modeling was not an appropriate approach for analyzing our data, as it is generally advised to have at least 10 level 2 observations (i.e., schools) when running HLM or MLM, with some research estimating that 50 or more clusters are necessary for accurately estimating effects (*71*–*74*). Data in the present study were from five schools (each with only one classroom in which the Geospatial curriculum was taught), which is below the recommended 10 level 2 observations. See the Supplementary Materials for more detailed descriptions of analyses.

### fMRI data acquisition

Imaging acquisition was performed on a Siemens 3T TIM Trio MRI scanner. All task fMRI data were acquired from T2*-weighted echoplanar imaging sequences (37 3.0-mm transversal slices; 64 × 64 matrix; repetition time = 2000 ms; echo time = 130 ms; field of view = 192 mm; 3.0 mm by 3.0 mm by 3.0 mm voxels; flip angle = 90°). To account for magnet stabilization, the first two volumes were excluded from analysis. High-resolution T1-weighted anatomical images (176 1.00-mm slices; 256 × 256 matrix; repetition time = 1900 ms; echo time = 2.52 ms; field of view = 250 mm; 1.0 mm by 1.0 mm by 1.0 mm; flip angle = 90°) were obtained for registration of functional data.

### fMRI data preprocessing

All fMRI data processing was carried out using FEAT (fMRI Expert Analysis Tool) version 5.98, part of FSL (FMRIB’s Software Library). General linear model–based analysis in FEAT uses FSL tools including Brain Extraction Tool (BET) (*75*), an affine registration tool, FMRIB’s Linear Image Registration Tool (FLIRT) (*76*, *77*), and a motion correction tool based on FLIRT (MCFLIRT) (*76*). FEAT carries out standard-space registration after time series statistics. FSL time series statistics correct for temporal smoothness by applying prewhitening (*78*). The following prestatistics processing was applied: spatial smoothing using a Gaussian kernel of full width at half maximum of 5 mm, grand-mean intensity normalization of the entire four-dimensional dataset by a single multiplicative factor, and high-pass temporal filtering (Gaussian-weighted least-squares straight line fitting, with sigma = 50.0 s). Registration to high-resolution structural and, subsequently, standard space images was performed using FLIRT.

### fMRI data analysis

#### Whole-brain activation analysis

At the individual subject level, a design matrix was fitted to each subject’s data as part of a general linear model with each condition modeled as events with a specified duration (i.e., the time from stimulus onset to onset of the response) convolved with a canonical hemodynamic response function. This was done separately for each task: EFT, MRT, and Reasoning. For all three tasks, we used a randomized, event-related design in which duration of each trial depended on how fast the participant responded during the response period (meaning that each trial for each participant was modeled in accordance with their actual onset and duration). At a group level, differences in whole-brain activation between the Geospatial and control groups were compared at both sessions (T1 and T2) using a mixed ANOVA model (Group-by-Time) for each of the above-indicated contrasts. This group level analysis was performed using FMRIB’s local analysis of mixed effects (*79*). Group-level analyses were conducted using FLAME1, a mixed-effects model implemented in FSL. FLAME1 is a relatively conservative method for multiple comparisons correction, which effectively mitigates inflated false positive rates (*80*). Corrections for multiple comparisons used Gaussian random field theory (voxel level: *Z* > 3.1, *P* < 0.001; cluster level: family wise error (FWE)-corrected threshold: *P* < 0.05). We subsequently masked the whole-brain results with the meta-analytic Neurosynth (*39*) association test map for the term spatial (SpatialMap).

#### Region of interest analysis

We used region of interest (ROI) analysis to extract levels of activation in the clusters that we identified in the whole-brain Group-by-Time analyses for EFT and Reasoning. This analysis was conducted using FSL’s featquery tool (https://fsl.fmrib.ox.ac.uk/fsl/fslwiki/FEAT/UserGuide#Featquery_-_FEAT_Results_Interrogation). First, we used the fslmaths tool to create masks of the clusters (from the corresponding Group-by-Time interaction *Z* statistic image). Second, we registered these cluster masks to the individual subject level. Third, we used the FSL featquery command to extract mean activation levels in clusters of interest during corresponding contrast. Mean percent signal change in activity was calculated at both time points (T1 and T2), where change in activation was calculated as T2 activation minus T1 activation.

#### Functional connectivity analysis

To examine changes in functional connectivity, we used PPI connectivity analysis in FSL (*81*). We used a standard PPI analysis procedure (*81*, *82*) that explicitly models and controls for overall task activation and hence models effective rather than synchronized task-related coactivation (*81*). We selected an a priori left DLPFC seed region from a neuroimaging meta-analysis of syllogistic verbal deductive reasoning (the form of reasoning used in the present reasoning task) (*12*) and constructed a 10-mm sphere around this peak voxel at the following Montreal Neurological Institute (MNI) coordinates: *X* = −45, *Y* = 35, and *Z* = 10. Our PPI analyses used three regressors: (i) a physiological variable representing the deconvolved time series within the left PFC seed region, (ii) a psychological variable representing the two task conditions, Reasoning versus Matching, and (iii) a PPI term that represented the cross-product of the first two regressors. Whole-brain Group-by-Time analyses of functional connectivity were performed as described above in the “Whole-brain activation analysis” section. We then extracted the degree of connectivity from the left DLPFC seed to brain regions overlapping with the SpatialMap map for each participant at both time points. Analyses were then completed in a similar fashion to the ROI analyses: We registered the masks to the individual subject level, extracted the mean level of connectivity for each participant at both time points and created a change variable for each SpatialMap-to-DLPFC analysis. This variable, which reflects change (from T1 to T2) in connectivity from SpatialMap regions to left DLPFC, during the Reasoning versus Matching contrast, was then used in all relevant correlation, regression, and mediation analyses.

#### Representational similarity analysis

RSA (*83*) was applied within the SpatialMap cluster (aIPS) in which Geospatial students showed increased activity during reasoning. We used the fslstats tool to extract mean activation levels from each voxel in the aIPS cluster during three conditions: spatial reasoning, nonspatial reasoning, and matching (control). For each of these conditions, activity from the baseline fixation condition was subtracted from task-related activity, such that each condition was a contrast (e.g., spatial reasoning > fixation baseline). Controlling for individual baseline activity in this manner is a statistical means of reducing the influence of elements of no interest, such as shared vascular, neural, and imaging elements often found in adjacent voxels (*84*). Then, Pearson’s partial correlations were computed between voxel activity (within the aIPS cluster) during spatial reasoning and nonspatial reasoning while controlling for activity in the matching control condition. This was done for each participant at both T1 and T2. Because *r* values are non-normally distributed, *r* values were next transformed using Fisher’s *z* transformation. All relevant statistics and analyses regarding this RSA were then computed using these *z* values as inputs for each participant at T1 and T2.

## Supplementary Materials

### This PDF file includes:

Supplementary Text Figs. S1 to S22 Tables S1 to S57 References

## Notes

View/request a protocol for this paper from *Bio-protocol*.
