# Supplementary material for: Transfer from spatial education to verbal reasoning and prediction of transfer from learning-related neural change
Source: Sci Adv. 2022 Aug 10;8(31):eabo3555. doi: 10.1126/sciadv.abo3555 (PMC9365289; doi:10.1126/sciadv.abo3555)
Supplement: Supplementary file 1 — Supplementary Text Figs. S1 to S22 Tables S1 to S57 References [file sciadv.abo3555_sm.pdf]

Science Advances
AAAS

Supplementary Materials for

Transfer from spatial education to verbal reasoning and prediction of transfer from learning-related neural change

Robert A. Cortes *et al.*

Corresponding author: Robert A. Cortes, rac114@georgetown.edu; Adam E. Green, aeg58@georgetown.edu

Sci. Adv. **8**, eabo3555 (2022)
DOI: 10.1126/sciadv.abo3555

This PDF file includes:

Supplementary Text
Figs. S1 to S22
Tables S1 to S57
References

Supplementary Text

Participants and Procedure

Student participants (*N*=346; 206 female, 140 male; mean age=16.61 years) were recruited from five public high schools in two northern Virginia counties). The schools were predominately located in suburban areas, with one school located in an urban area. Average demographic data for public schools in the two counties indicate that slightly fewer than 50% of students are White and approximately 15-30% qualify for free or reduced-price lunch. All procedures were approved by the Georgetown University institutional review board. All enrolled participants reported no history of psychoactive drug use, psychiatric diagnosis, or brain damage. All interested students provided informed assent, with informed consent from legal guardians for students under 18 years of age. After a pre-test phase in which background measures of demographics, spatial abilities, and interests were collected, 12 Geospatial students chose not to participate in the main study, and 124 participants not enrolled in the Geospatial course (potential Controls) were excluded from participating in the control group because they could not be matched to Geospatial students on the basis of propensity scoring (see Propensity Scoring Methods). Thus, 210 participants were recruited to participate in the main study. Of these 210 participants, 23 participants did not complete the second time point of the main study (T2) and were therefore excluded from analyses.

Three participants were removed because data were not properly recorded. Data were also excluded for a participant who reported an autism spectrum diagnosis (after reporting no diagnosis on an initial screening for psychiatric history), and another who reported being on prescription pain medication during a testing session soon after major surgery. To ensure appropriate attention and understanding of behavioral tasks (see below), minimum quality control standards of accurate responding on at least 1/3 of trials, and average response time of at least 500 milliseconds were applied (in all cases, these thresholds were more than 3 standard deviations below the mean). Data for one participant in the Embedded Figures Task and one participant in the Mental Rotation Task were excluded for accuracy below the minimum threshold. Robustness checks indicated that none of these exclusions meaningfully affected the results reported in the main text.

The final sample thus included 182 participants who participated in two testing sessions within the main study. The Geospatial group included 77 students (32 female, 45 male; mean age=16.66 years). The Control group included 105 students (56 female, 49 male; mean age=16.63 years). T1 took place during the summer before the start of the Geospatial course (i.e., before the school year). T2 took place following completion of the Geospatial course (i.e., after the school year). The study was conducted over two consecutive school years (2015-2016 and 2016-2017). During both main study testing sessions, participants completed the following tasks (see Behavioral Tasks): Mental Rotation (adapted from Shepard & Metzler) (*26*, *34*), Embedded Figures (adapted from Witkin) (*25*, *33*), a verbal relational reasoning task (adapted from Knauff) (*31*, *85*), and the Spatial Habits of Mind Inventory (*28*) (completed at pre-test and T2). For behavioral participants, testing sessions were conducted either at Georgetown University or at their high school. For neuroimaging participants, testing sessions were conducted at the Georgetown Center for Functional and Molecular Imaging. For the 63 neuroimaging participants (Geospatial: 32 Total, 13 Female 19 Male, mean age=16.36; Control: 31 Total, 18 Female, 13 Male, mean age=16.60), the embedded figures, mental rotation, and verbal relational reasoning tasks were completed during functional magnetic resonance imaging (fMRI) at both time points.

Propensity Scoring Methods

A challenge for real-world education research is that random assignment to conditions (where the conditions are the courses students take) is generally not possible. In order to minimize potential selection bias in the present study, we employed a quasi-experimental design in which propensity scoring methods (*22*) were used to match the Geospatial group with the control group on their overall 'propensity' to take the Geospatial course. Propensity scoring methods (PSM) are effective when the number of prospective control participants is large relative to the number of prospective treatment participants. In the first wave/year of recruitment, we used PSM to narrow the pool of control students. In the second wave/year of recruitment, the proportion of prospective control to Geospatial Semester participants was similar. Therefore, all students in wave/year 2 were invited to participate. In both waves/years, all Geospatial Semester students who returned consent forms were invited to participate. We first collected relevant background measures of experience, interests, demographics, academic performance, spatial thinking abilities, and frequency of spatial thinking strategy use in the pre-test phase from the full initial sample of 346 total participants (which included both Geospatial students and potential control participants). Specifically, we collected the following at the pre-test phase: Gender, Race, Age, Mother's level of education, Frequency of video game use per week, Whether they had previously taken a course with the Geospatial course teacher at their high school, Competency with geographic information software (GIS), PSAT score, Overall GPA, Math GPA, Science GPA, Paper Folding Task (*86*) (measure of spatial ability), Spatial Habits-of-Mind Inventory (*28*) (measure of frequency of spatial thinking strategy use), and Childhood Activity Questionnaire (*87*) (CAQ; measure of engagement in childhood spatial and motor activities, such as sports). Summary statistics for these measures in the full sample (and separately for Geospatial and Control students) can be found in Table S1.

To generate propensity scores, we implemented both 1) a Leaps & Bounds selection process (*88*) to identify covariates that would lead to the best model fit, and 2) inclusion of theoretically important variables, making incremental adjustments after each process to verify if addition or subtraction of variables would lead to better model fit. This process was conducted separately for each county (County 1 and County 2) because they varied demographically (County 1 was suburban-rural and County 2 was urban-suburban) and the majority of our sample came from County 1. Hence, it was possible that different demographic and spatial thinking measures might differentially predict which students enrolled in the Geospatial course in the respective counties. The following variables were identified as the best predictors of GSS enrollment and thus included as predictors to create propensity scores. For County 1: Gender, Race, having taken a class with the Geospatial Course teacher previously, Paper Folding ability, and Math GPA. For County 2: Gender, and Paper Folding ability.

Once propensity scores were created, we then used a 1-5 nearest neighbor matching approach (*23*) with replacement and calipers to restrict matches to Control students with propensity scores that were within 0.15 SD of the Geospatial student's score. This means that although we allowed each Geospatial student to be matched to up to 5 control students, if there were no cases with a propensity ±0.15 SD of the treatment student's estimated score, then no matches would be made. All students enrolled in the Geospatial Semester were invited to participate. Control students were contacted in the order of propensity score match. That is, students with the highest propensity scores, indicating that they were a close match to multiple Geospatial Semester participants, were invited first. Out of 346 participants who completed pre-test, 12 Geospatial participants chose not to participate in the main study, and 124 participants were excluded from participating in the control group because they could not be adequately matched to a Geospatial student. Of the remaining 210 participants, 23 did not complete T2, and five were excluded for data recording errors or poor data quality (see above), resulting in a final sample of 182 participants. Propensity

score matching was successful, as indicated by statistically equivalent Geospatial and control groups (i.e. non-significant t-tests) (*29*) in our final behavioral sample (N=182) for all the pre-test variables (Table S1).

We also sought to ensure similarity between the Geospatial and Control samples by limiting recruitment of Control participants to juniors enrolled in the same schools from which we recruited the Geospatial sample. Importantly, in addition to the effect of propensity score matching of reducing selection bias, the potential for any additional selection bias effects were also mitigated by the difference-of-differences experimental paradigm employed. That is, the study was primarily concerned with difference-of-differences outcomes that reflected change from T1 to T2 rather than measures of performance/ability at a single timepoint (*89*), so key outcomes reflect learning-related changes in experimental measures rather static individual differences in abilities.

The Geospatial Course

The Geospatial Course (*24*) was created in 2005 as a partnership between public high schools in Virginia and the Integrated Science and Technology department at James Madison University (JMU). The Geospatial Course is a dual-enrollment course, in which students take the course at their local high school (for either 45 minutes daily or 90 minutes every other day) and receive college credit from JMU. JMU faculty mentor the high school teachers, providing technical support, teaching and observing classes, and assisting in mentoring students on their course projects. The Geospatial Course curriculum is designed to enhance spatial thinking skills and foster increased use of spatial thinking strategies through the use of geospatial technology such as Geographic Information Systems (e.g., ArcGIS; www.arcgis.com). Students had to maintain at least 70% attendance to receive a grade in the course. The estimated total time spent in the Geospatial course was 135 hours (9 months x 20 days/month x .75 hours/day)—the control group spent the same amount of time in their respective courses. More information about the Geospatial course is provided at https://www.isat.jmu.edu/geospatialsemester/.

The majority of curricular content involves working with (e.g., evaluating, generating, and comparing) spatial representations in the form of maps. These maps enable visualization of spatial relationships between a wide range of real-world variables relevant to, e.g., population, demographics, climate and weather, industrial activity, and health and safety. The purpose is to determine how these variables, represented by visuo-spatial features (usually shapes), relate to each other in space (i.e., within the space of the maps), and how their spatial relationships are similar or different in different geographic locations and/or different times in history. Identifying and relating key features to each other (within and between maps) enables inferences about the causes of data relationships, and provides bases for projecting future relationships and suggesting spatial design-based solutions to current or projected problems. Some examples from the course include mapping the extent of storm surge, mapping evacuation routes following a potential disaster, determining where to locate off-shore windmills, and optimizing strategic placement of fire stations throughout a local county. The course culminates in an extensive final project in which students work independently or in pairs to use spatial information and spatial representations to identify and explore a question of interest, often in their own geographic region. See Fig. S1 for examples of the kinds of maps created and used in the course. The course is designed to foster a range of spatial thinking skills, and great emphasis is placed on interpreting maps by spatially scanning for key features and relating spatial features and relationships within and across maps. Spatial habits-of-mind are developed through the practice of using (and seeing the effectiveness of teachers and other students using) spatial thinking strategies to understand real-world data relationships and to support real-world problem solving.

In the present study, the intervention was the Geospatial course itself; that is, it was simply a course offered by the schools, as opposed to any sort of program or training outside the school curriculum. The structure and content of the curriculum was completely un-altered by the researchers to ensure that the study reflected an ecologically valid classroom implementation of spatial education, rather than a laboratory-based intervention.

Behavioral Tasks

Verbal Relational Reasoning Task

A computerized verbal relational reasoning task (*32*, *85*, *90*) was used to measure deductive reasoning ability closely linked to mental modeling (*10*, *32*). Stimuli were taken from Ruff et al., (*31*). Each task trial contained three sentences: two premises and a conclusion. Each premise described a relation between two animals (e.g., "The Dog is better than the Cat"), and participants indicated whether the conclusion followed logically from the premises by pressing keys for either "Yes" or "No". There were 60 total trials: 40 reasoning problems and 20 matching problems. Of the 40 reasoning trials, 20 involved spatial relations (e.g., above/below) and 20 involved nonspatial relations (e.g., better/worse). The matching problems required participants to determine whether the conclusion exactly matched either of the two premises. These matching items were devised as a control condition (*31*) for neuroimaging analyses; performance on matching trials was not used to compute behavioral performance scores for this task. Across all trial types, half were True and half were False. A single letter was presented before each trial to indicate whether it was a reasoning trial or matching trial: participants were shown 'R' before Reasoning trials and 'M' before Matching trials. The premises and conclusion then appeared (first premise alone for 3000 ms, then both premises for 3000 ms, then both premises and the conclusion for 8000 ms). The complete timing of the phases of each trial is indicated in Fig. S2. Once the conclusion was presented, participants were given up to 8 seconds to respond. If they responded before 8 seconds, the screen advanced to the next trial. Jittered fixations ranging from 4 to 5 seconds were presented between trials. The fMRI version of the task was identical to the behavioral version, except that it was presented in the fMRI scanner. Behavioral performance scores for the Reasoning task were again computed as the number of correct responses per second. Descriptive statistics for Reasoning are shown in Table S8. Results from the Group (Geospatial, Control) by Time (T1, T2) ANOVA for Reasoning (covarying Gender, GPA, and PSAT) are shown in Table S3; results for nonspatial reasoning trials only are shown in Table S4.

Embedded Figures Task (EFT)

A computerized version of the Embedded Figures Task (*25*) was used to assess participants' spatial scanning ability. Stimuli were taken from Walter & Dassonville (*33*). In the EFT, participants determine whether a target figure is embedded within a more complex figure (see Fig. S3). Participants were given up to 10 seconds to respond to each trial by pressing keys to indicate "Yes" (if the simple shape could be found within the complex shape) or "No" (if it could not). If they responded before 10 seconds, the screen advanced to the next trial. There were 30 total trials, two-thirds of which were True. Thirty 'match' trials were also initially included as control trials for fMRI contrasts but, in post-study debriefing interviews, participants consistently reported solving match trials in the same fashion as the search trials (i.e., by applying spatial scanning) indicating that they were not appropriate controls for isolating spatial scanning processes. We therefore did not use Match trials in any fMRI or behavioral analyses; Search trials were compared to Baseline Fixation in the fMRI analyses, as in prior studies (*66*, *67*). Jittered fixations ranging from 2 to 4 seconds were presented between trials (Fig. S3). The fMRI version of the task was identical to the behavioral version, except that it was presented in the fMRI scanner.

Behavioral performance scores for the EFT (and all behavioral tasks) was computed as the number of correct responses per second (Rate Correct Score;(*65*); see below). Descriptive statistics (accuracy, reaction time, and rate correct score) for EFT are shown in Table S7. Results from the Group (Geospatial, Control) by Time (T1, T2) ANOVA for EFT (covarying Gender, GPA, and PSAT) can be seen in Table S2.

Mental Rotation Task (MRT)

A computerized version of the Mental Rotation Task (MRT) developed by Shepard & Metzler (*26*, *34*) was used to assess mental rotation of objects. Stimuli were taken from Shepard & Metzler (*26*, *34*). In each trial, participants saw two images of 3-dimensional objects (Fig. S4) and were given up to 7 seconds to decide whether they were rotated images of the same object vs. images of different objects by pressing keys for either "Yes" (the images show the same object) or "no" (the images show different objects). If participants responded before 7 seconds, the screen advanced to the next trial. To create conditions of varying difficulty, objects were shown at three different angles of rotation relative to each other (50, 100, 150 degrees); there were 24 trials of each rotation angle (72 total trials). We also included 12 trials with 0 degrees of rotation as a control condition. Thus, there were a total of 84 trials. Following previous implementations of MRT (*68*), we used a 2:1 ratio of True (same object) to False (different objects) trials across all trial types. Jittered fixations ranging from 3 to 8 seconds were presented between trials (Fig. S4). The fMRI version of the task was identical to the behavioral version, except that it was presented in the fMRI scanner. Behavioral performance scores for the MRT were again computed as the number of correct responses per second. Descriptive statistics for MRT are shown in Table S9. Results from the Group (Geospatial, Control) by Time (T1, T2) ANOVA for MRT (covarying Gender, GPA, and PSAT) are shown in Table S5.

Spatial Habits of Mind Inventory (SHOMI)

A paper form of the Spatial Habits of Mind Inventory (*28*) was used to measure spatial strategy use. The SHOMI is a self-report survey composed of 28 items which assess the extent to which individuals engage in essential dimensions of spatial thinking strategy use, including pattern recognition, spatial description, visualization, spatial concept use, and spatial tool use. The Geospatial curriculum studied here was devised to foster spatial "habits-of-mind," and previous work has demonstrated that the Geospatial curriculum was associated with improved SHOMI scores compared to a control science curriculum (*28*). For each item, participants read a statement about their own spatial strategy use and rated the extent to which they agreed with the statement on a scale of 1-5. Items presented with negative phrasing (i.e., describing less spatial strategy use) were reverse coded. A total sum score was calculated by adding rated agreement for all 28 items, where the highest possible score was 140. In contrast to the other behavioral measures (which were administered at T1 and T2), SHOMI was administered at pre-test and T2. Descriptive statistics for SHOMI are shown in Table S10. Results from the Group (Geospatial, Control) by Time (T1, T2) ANOVA for SHOMI (covarying Gender, GPA, and PSAT) can be seen in Table S6.

Counterbalancing, Randomization, and Practice

To avoid confounds related to item- and order-specific effects, there were two versions (A and B) of the EFT, MRT, and Reasoning tasks; each version contained the same type and number of trials, but used a different set of stimuli (e.g., different objects in the MRT, different shapes in the EFT, and different sentences in the Reasoning task) and a different trial order. The order of the tasks was also counterbalanced by timepoint, such that version A was ordered EFT, MRT, Reasoning and version B was ordered Reasoning, MRT, EFT. These versions were counterbalanced randomly across all participants at both timepoints, such that half of the

participants completed version A at T1 and version B at T2 (and vice versa for the other half). Within each task, the duration of the response phase of each trial depended on how quickly the participant responded (i.e. self-advancing).

Both behavioral-only and neuroimaging participants completed instructions and practice for EFT, MRT, and Reasoning task on a laptop computer before beginning the actual tasks (neuroimaging participants completed instructions and practice outside of the scanner immediately before the scanning session). For each task, participants received detailed instructions, were given opportunities and prompts to ask questions about each task, and completed 10 practice problems. The order of the tasks instructions was also counterbalanced by timepoint (as described above).

PSAT

The Preliminary Scholastic Aptitude Test (PSAT) is a validated measure of college readiness used to determine whether students qualify for the National Merit Scholarship Program in the United States (*91*). PSAT scores in the present dataset represented the composite (sum) score from both the reading and math sections of the PSAT (range=40 to 160). Students complete the PSAT free-of-charge at their school. PSAT scores were obtained from students' high school record archives in accordance with pre-approved IRB protocol.

GPA

Grade Point Average (GPA) measured the average grade points earned in high school prior to enrollment in the study (range=0 to 4.3). GPA values were obtained from student's high school record archives in accordance with pre-approved IRB protocol.

Geospatial Course Grades

Grades for the Geospatial course reflected students' final grade in the course (range=0 to 4.0). Grades were generally high (78.4% A or A+, lowest was a single B- grade). This indicates that, based on the teachers' assessments, all Geospatial students in the study successfully learned the content of the Geospatial curriculum. The clustering of scores around ceiling, however, represented a limited range and a Shapiro-Wilk normality test indicated that grades were not normally distributed (W=0.71553, $p < .001$). Therefore, we transformed the Geospatial course grade variable into a binary variable where 1=received an A or higher and 0=received a grade lower than A. 78.4% of Geospatial students received an A and 21.6% received a grade lower than A.

Rate Correct Score

Rate correct score (RCS), a composite measure of accuracy and response time (*65*, *92*), was used to measure behavioral performance on EFT, MRT, and Reasoning. RCS is calculated as frequency of correct responses of considered trials divided by total reaction time spent on considered trials, and is interpreted as number of correct responses per unit time (*92*). When the same (or largely overlapping) cognitive abilities/resources support task performance in the forms of both faster responding and more accurate responding, composite measures integrating RT and accuracy yield an integrated effect size that recovers information present in both component measures, and RCS appears to perform especially well in this regard (*65*). This enables composite measures to account for a larger proportion of the variance than the separate component measures individually (*65*). RCS is most appropriate when accuracy is relatively high (*65*), which was the case for all three tasks at both timepoints (T1: EFT average accuracy=.71, MRT average accuracy=.78, Reasoning mean accuracy=.82; T2: EFT average accuracy=.73, MRT average accuracy=.81, Reasoning mean accuracy=.84), and when RT and accuracy are not positively correlated (*65*) (when faster performance is not correlated with decreased accuracy; i.e. no speed-

accuracy tradeoff). In the full behavioral sample (*N*=182), there was no positive association between RT and accuracy at any time point for any task and, in fact, directionally negative associations were observed at all timepoints for all tasks (significant in most cases), indicating the appropriateness of RCS for the present data. This same pattern was also observed in the neuroimaging sample (*N*=63). This pattern is consistent with our *a priori* theoretical consideration of these tasks. In particular, we anticipated that accuracy and reaction time would both reflect underlying processing advantages in the spatial brain. For example, if deployment of spatial resources (e.g., spatial attention) supports scanning for relevant properties of spatial features (in complex figures or mental models of premises), then features should be identified, or appropriately dismissed, more efficiently (supporting response time), and the features that are identified should more frequently provide accurate indicators (supporting accuracy). Note that these elements of processing the stimuli are not independent of each other (e.g., inaccurately identifying relevant features on a first scan often requires additional scanning, thereby increasing response time), again indicating that composite measurement best reflects the meaningful variance in the data (i.e., variance in the processing that underlies performance).

A recent study comparing seven composite performance measures (*65*) indicated that RCS and a measure developed by that study's author (LISAS) were the most effective in detecting meaningful effects and accounted for a larger proportion of the variance than other composite measures. Specific features of RCS made it preferable to LISAS in the present study. RCS can be calculated for the full range of accuracy for each participant on every task, whereas LISAS cannot be calculated when accuracy is at ceiling (i.e. 100%) for an individual participant on an individual task because the denominator becomes zero. Thus, one of the reasons RCS was preferable to LISAS in the present study was that using LISAS would have required us to throw out or artificially adjust data (or to use RT alone) in instances where any individual participant performed at 100% for any individual task at any time point. Although no task showed ceiling effects overall, there were several instances in which an individual participant answered correctly on all trials for individual tasks at an individual time point. In addition, RCS values are intrinsically interpretable (i.e., each RCS value conveys the number of correct responses per unit time), whereas individual LISAS values do not have any direct interpretation. A subsequent study by the same author (*93*) indicated that LISAS provided a closer approximation of effect sizes for within-subject response time and accuracy comparisons in a task-switching paradigm involving fine-grained response time differences. Reanalyzing the behavioral data in the present study using LISAS in place of RCS did not alter any of the results reported in the main text (e.g., all significant Group-by-Time interactions and associations remained significant). We additionally found that RCS and LISAS effect sizes were highly correlated across all within-subject and between-subject comparisons (r=.98), and did not differ from each other for within-subject or between-subject effect sizes (all p>.90), and that correlations with accuracy and response time effect sizes did not differ for RCS vs. LISAS (all p>.85). Recent work using complex cognitive tasks with longer response times in an individual-differences paradigm (similar to the present study, but unlike the task switching paradigm studied with LISAS) also found RCS to appropriately reflect meaningful variance (*94*).

Statistical Analysis

All statistical analyses were computed in R-studio (*69*) and SPSS Version 27 (*70*).

Behavioral Task Performance Analysis

Analyses of task performance focused on testing whether Geospatial students showed greater longitudinal improvement in performance (from T1 to T2) than Controls (i.e., Group-by-Time

interactions). Analysis of Variance (ANOVA) results for each of the behavioral tasks, covarying Gender, GPA, and PSAT, can be seen in Tables S2-6.

Regression Models
Results of the regression models relating change in performance on the Reasoning transfer task (ΔReasoning) to change in performance on spatial tasks (ΔEFT, ΔMRT, ΔSHOMI) can be found in Tables S11-17. Results of the regression models relating neural change variables (ΔaIPS_EFT, ΔIPL_EFT, Change in SpatialMap-to-DLPFC Connectivity during Reasoning, ΔaIPS_Reasoning, and RSA-based change in similarity between nonspatial and spatial relation Reasoning trials in aIPS) to behavioral performance variables (ΔEFT and ΔReasoning) can be found in Tables S26-32.

Mediation Models
All mediation models were computed in R-studio using the package 'mediation' (*95*). Bootstrapping confidence intervals were set to 95% with 500 Monte Carlo draws, and standardized indirect effects were estimated with ACME (*95*, *96*). All mediation analyses covaried GPA, PSAT, and Gender. Mediation models and results for analyses considering behavioral change in spatial scanning performance (ΔEFT) as the mediator, Geospatial vs. Control as the independent variable, and behavioral change in reasoning performance (ΔReasoning) as the dependent variable can be found in Figs. S5-6. Behavioral change in mental rotation performance (ΔMRT) was not considered as a mediator because this variable was not associated with the Geospatial curriculum (i.e., it was not associated with the independent variable). Models considering neural changes as mediators can be found in Figs. S12-15. See Neural Change Mediation Analyses below for more detailed descriptions of these models.

fMRI Data Acquisition
Imaging acquisition was performed on a 3T Siemens Trio Tim MRI scanner. All task fMRI data were acquired from T2*-weighted echoplanar imaging sequence (37 3.0 mm transversal slices; 64 x 64 matrix; repetition time=2000 ms; echo time=30 ms; field of view=192 mm; 3.0 x 3.0 x 3.0 mm voxels; flip angle=90 degrees). In order to account for magnet stabilization, the first 2 volumes were excluded from analysis. High-resolution T1-weighted anatomical images (176 1.00 mm slices; 256 x 256 matrix; repetition time=1900 ms; echo time=2.52 ms; field of view=250 mm; 1.0 x 1.0 x 1.0 mm; flip angle=90 degrees).

fMRI Data Preprocessing
All fMRI data processing was carried out using FEAT (fMRI Expert Analysis Tool) Version 5.98, part of FSL (FMRIB's Software Library). General Linear Model-based analysis in FEAT uses FSL tools including Brain Extraction Tool (BET) (*75*), an affine registration tool, FMRIB's Linear Image Registration Tool (FLIRT) (*76*, *77*), and a motion-correction tool based on FLIRT (MCRFLIRT) (*76*). FEAT carries out standard-space registration after time-series statistics. FSL time-series statistics correct for temporal smoothness by applying pre-whitening (*78*). The following pre-statistics processing was applied: spatial smoothing using a Gaussian kernel of FWHM 5 mm; grand-mean intensity normalization of the entire 4D dataset by a single multiplicative factor; highpass temporal filtering (Gaussian-weighted least-squares straight line fitting, with sigma=50.0s). Registration to high resolution structural and, subsequently, standard space images was performed using FLIRT.

fMRI Data Analysis

Whole-Brain Activation Analysis

At the individual subject level, a design matrix was fitted to each subject's data as part of a general linear model (GLM) with each condition modeled as events with a specified duration (i.e., the time from stimulus onset to onset of the response) convolved with a canonical hemodynamic response function. This was done separately for each task: EFT, MRT, and Reasoning. For all three tasks, we utilized a randomized, event-related design in which duration of each trial depended on how fast the participant responded during the response period (meaning that each trial for each participant was modeled in accordance with their actual onset and duration). In EFT and MRT, the trials of interest were modeled using the onset-duration of the entire response period. In the Reasoning task, both the reasoning and matching trials were modeled using the entire trial (premise 1, premise 2, and conclusion/response period). On average, full reasoning trials (events) lasted an average of 9083ms (SD=918ms) and matching trials lasted an average of 8493ms (SD=760ms). Voxelwise contrast and z-statistics images for each task were generated for each participant using the following contrasts: EFT: Search>Baseline (fixation), MRT: All Rotation True (50, 100, & 150 degree rotation true)>0 degree true; Reasoning: Reasoning>Matching.

At a group level, differences in whole-brain activation between the Geospatial and Control groups were compared at both sessions (T1 and T2) using a mixed ANOVA model (Group-by-Time) for each of the above-indicated contrasts. This group level analysis was performed using FMRIB's Local Analysis of Mixed Effects (*79*). Group-level analyses were conducted using FLAME1, a mixed-effects model implemented in FSL. FLAME1 is a relatively conservative method for multiple comparisons correction, which effectively mitigates inflated false positive rates (*80*). Corrections for multiple comparisons used gaussian random field theory (Voxel level: $Z > 3.1$, $P < .001$; Cluster level: FWE-corrected threshold: $P < 0.05$).

Gender analyses for EFT and Reasoning task activation were conducted using a GLM that modeled the three-way Group-by-Time-by-Gender interaction, thus indicating regions where female students (relative to males) in the Geospatial course (relative to Control) showed changes in activation at T2 (relative to T1).

Whole-brain results for EFT can be seen in Fig. S7 and Table S18 (no significant clusters emerged for the Control>Geospatial contrast), Reasoning in Fig. S8 and Tables S19-20, and MRT in Fig. S9 and Tables S21-22. Because Reasoning was not an overtly spatial task (unlike EFT and MRT), and our hypotheses concerned changes in spatial cognition, only clusters emerging from the whole-brain analysis that overlapped with the *a priori* Neurosynth-based meta-analytic map for the term “spatial” (*39*) (henceforth “SpatialMap”) were considered.

Region of Interest Analysis

We used Region of Interest (ROI) analysis to extract levels of activation in the clusters we identified in the whole-brain Group-by-Time analyses for EFT and Reasoning. This analysis was conducted using FSL’s featquery tool. First, we used the fslmaths tool to create masks of the clusters (from the corresponding Group-by-Time interaction Z statistic image). Second, we registered these cluster masks to the individual subject level. Third, we used the FSL featquery command to extract mean activation levels in clusters of interest during corresponding contrast. Mean percent signal change in activity was calculated at both timepoints (T1 and T2), where change in activation was calculated as T2 activation minus T1 activation.

Functional Connectivity Analysis

To examine changes in functional connectivity, we employed psychophysiological interaction (PPI) connectivity analysis in FSL (*81*). We used a standard PPI analysis procedure (*81, 82*) that

explicitly models and controls for overall task activation and, as such, models effective rather than synchronized task-related coactivation (*81*). We selected our *a priori* seed regions from the most recent and comprehensive neuroimaging meta-analysis to our knowledge of verbal deductive reasoning(*97*). We selected an *a priori* left dorsolateral prefrontal cortex (DLPFC) seed region from a neuroimaging meta-analysis of syllogistic verbal deductive reasoning (*12*) (the form of reasoning utilized in the present reasoning task), and constructed a 10mm sphere around this peak voxel at the following MNI coordinates: X= -45,Y=35, Z=10. Our PPI analyses used three regressors: (1) a physiological variable representing the deconvolved time series within the left PFC seed region; (2) a psychological variable representing the two task conditions—Reasoning versus Matching; and (3) a PPI term that represented the cross-product of the first two regressors.

Whole brain Group-by-Time analyses of functional connectivity were performed as described above in the Whole Brain Activation Analysis section. We then extracted the degree of connectivity from the left DLPFC seed to brain regions overlapping with the SpatialMap map for each participant at both timepoints. Analyses were then completed in a similar fashion to the ROI analyses: we registered the masks to the individual subject level, extracted the mean level of connectivity for each participant at both timepoints and created a change variable for each SpatialMap-to-DLPFC analysis. This variable, which reflects change (from T1 to T2) in connectivity from SpatialMap regions to left DLPFC, during the Reasoning versus Matching contrast, was then used in all relevant correlation, regression, and mediation analyses.

Gender analyses were conducted using GLM to model the three-way Group-by-Time-by-Gender interaction, thus indicating regions where female students (relative to males) in the Geospatial course (relative to Control) showed changes in SpatialMap-to-DLPFC connectivity at T2 (relative to T1). As described above, gender analyses were conducted separately for each PFC seed, and combined afterwards into one connectivity map.

Whole-brain results (with SpatialMap map overlayed) for left DLPFC connectivity during Reasoning can be seen in Fig. S10 and Table S23 (no significant clusters emerged for the Control>Geospatial contrast), and results from the Group-by-Time-by-Gender interaction described above can be seen in Fig. S11 and Table S24 (no significant clusters emerged for the Male Geospatial>Female Geospatial contrast). Additional exploratory analyses with right DLPFC as a seed region found no significant increased connectivity (in Geospatial>Control contrast).

Representational Similarity Analysis
To further examine changes in the students' neural representation of reasoning problems involving spatial vs. nonspatial relations, representational similarity analysis (RSA) (*83*) was applied within the SpatialMap cluster (aIPS) in which Geospatial students showed increased activity during reasoning. We used the fslstats tool to extract mean activation levels from each voxel in the aIPS cluster during three conditions: spatial reasoning, nonspatial reasoning, and matching (control). For each of these conditions, activity from the baseline fixation condition was subtracted from task-related activity, such that each condition was a contrast (e.g., Spatial Reasoning>Fixation Baseline). Controlling for individual baseline activity in this manner is a statistical means of reducing the influence of elements of no interest, such as shared vascular, neural, and imaging elements often found adjacent voxels (*84*). Then, Pearson's partial correlations were computed between voxel activity (within the aIPS cluster) during spatial reasoning and nonspatial reasoning, while controlling for activity in the matching control condition. This was done for each participant at both T1 and T2. Because r values are non-normally distributed, r values were next transformed using Fisher's z-transformation. All relevant

statistics and analyses regarding this RSA were then computed using these z values as inputs for each participant at T1 and T2.

We predicted that neural representation of nonspatial information during reasoning would become "spatialized" in Geospatial students – representations of nonspatial relations (e.g., "better") would become more similar to spatial relations (e.g., "above"); these trial types were devised to test this prediction. Consistent with the predicted change in neural representation, in aIPS, Geospatial students showed increased similarity between nonspatial and spatial relation trials from T1 to T2 (t=3.43, p=.001, paired t-test). Further analysis showed that nonspatial relation trials at T1 were significantly less similar to nonspatial relation trials at T2 than spatial relation trials at T1 were to spatial relation trials at T2 (t=2.44, p=.018; i.e. nonspatial relation trials changed more from T1 to T2 than did spatial relation trials), suggesting that increased nonspatial-to-spatial similarity was because the representation of nonspatial relations became more like nonspatial trials. aIPS also showed a greater increase in similarity between nonspatial and spatial trials in Geospatial students relative to Controls (Group-by-Time interaction F=4.71, p=.034; Table S25).

<u>Neural Change Mediation Analyses</u>

We tested whether each of the neural changes that was associated with improved behavioral task performance statistically mediated the association between taking the Geospatial curriculum (i.e., the Geospatial vs. Control group variable) and improved performance. For EFT, mediation analyses (Bootstrapping 95% confidence interval) revealed that ΔaIPS_EFT mediated the association of the Geospatial curriculum to ΔEFT (indirect effect p<.001, Fig. S12), and that ΔIPL_EFT also mediated the association of the Geospatial curriculum to ΔEFT (indirect effect p<.001, Fig. S13). For Reasoning, mediation analyses (Bootstrapping 95% Confidence Interval) revealed that ΔaIPS_EFT mediated the association of the Geospatial curriculum to ΔReasoning (indirect effect P<.001, Fig. S14), and that ΔIPL_EFT also mediated the association of the Geospatial curriculum to ΔReasoning (indirect effect p<.001, Fig. S15). We additionally tested whether the neural change during Reasoning that was associated with improved Reasoning performance (ΔSpatialMap-to-DLPFC connectivity) mediated the association between the Geospatial course and ΔReasoning. Mediation analysis (Bootstrapping 95% Confidence Interval) revealed that ΔSpatialMap-to-DLPFC connectivity did not significantly mediate the association of the Geospatial curriculum to ΔReasoning (indirect effect p=.088).

<u>Predictive Modeling (Ensemble Model Analysis)</u>

A key objective of this study was to test curriculum-related longitudinal neural change as a marker of transferable learning, compared to traditional performance-based assessments such as academic testing, cognitive testing, and grades. To do this, we used a data-driven prediction modeling approach. Note that we use the term, prediction, here to refer to statistical prediction of curriculum-related change in task performance on the basis of cognitive and brain-based measures collected for all tasks at the same two time points, as opposed to prediction of future outcomes. Curriculum-related neural changes were included as predictors alongside performance-based assessments to identify which predictors contributed most to prediction modeling of change in the verbal reasoning transfer measures (ΔReasoning), and to compare the predictive value of neural changes vs. performance-based assessments. For these analyses, we utilized ensemble modeling via Automatic Linear Modeling in SPSS 27 (*47*). This adaptive machine-learning approach assesses the respective contributions ("importance") of predictor variables across an ensemble model (comprising possible permutation models involving the predictor variables). We employed both a "boosting" (*70*, *98*) approach to maximize model accuracy, and a standard model creation approach to identify the predictor variables that constitute the most informative model (Akaike Information Criterion). Data for participants to be included in each analysis (e.g., only fMRI

participants, only Geospatial students) were separated from the full data set prior to running the analysis in order to avoid unintended consideration of other participants during default data preparation. We used the following settings for Automatic Linear Modeling: /build_options objective=boosting (for boosting analysis to optimize model accuracy),=standard (for analyses to build standard models); use_auto_data_preparation=true; confidence_level=95; model_selection=none (for boosting analysis),=bestsubsets (for analyses to build standard models); criteria_best_subsets=aicc; replicate_results=true; seed=54752075 /ensembles combining_rule_continuous=mean; component_models_n=10.

The neural changes included in the prediction modeling analyses were the neural changes in the embedded figures task (EFT) that emerged as Group-by-Time interactions at the whole-brain level, and were subsequently found to be associated with longitudinal improvement in reasoning (ΔReasoning) in models in which ΔReasoning was regressed on each potential neural change. Note that neural change observed during Reasoning also emerged as a Group-by-Time interaction and was associated with ΔReasoning (i.e., SpatialMap-to-DLPFC connectivity), however this neural change was not used in predictive-modeling analyses because the goal of this analysis – using a modeling method intended to enable predictive inference – was to identify neural changes that could *predict* transfer to Reasoning. If the transfer task itself (Reasoning) is required in order to obtain a neural measure of transferable learning, then it is not clear that a meaningfully predictive inference can be made. Neural change during Reasoning has substantial value for elucidating neural mechanism-of-change associated with transfer from spatial education to reasoning, but it does not have clear value as a predictor of transfer. For the predictive-modeling analyses, we instead sought to test whether the transferability of spatial learning could be predictively marked by neural measurement of the separate EFT task, which reflects a construct (spatial scanning) that we hypothesized would support reasoning.

We sought to directly compare curriculum-related neural change to curriculum-related performance change (i.e., neural vs. performance-based assessment of curriculum-related learning). Thus, in comparison to neural change on EFT, a key predictor in these models was performance change on EFT (ΔEFT). The inclusion of ΔEFT as a predictor was important for several reasons: 1) EFT measured spatial scanning ability. Development of spatial scanning was a primary learning goal of the Geospatial curriculum (*24*) (see above). Thus, ΔEFT was a behavioral outcome that measured curriculum learning in the Geospatial course. 2) ΔEFT showed a Group-by-Time interaction such that Geospatial students improved more than controls in their spatial scanning performance from T1 to T2. This indicated that the Geospatial curriculum achieved the learning goal of improved spatial scanning performance. 3) Improved spatial scanning performance (ΔEFT) was a theoretically-grounded predictor of transferable learning based on the Mental Model Theory claim that spatial scanning supports mental modeling in overtly nonspatial tasks, including verbal reasoning (*10*). 4) ΔEFT was strongly predictive of improved verbal reasoning ($p<.001$; i.e., the more students' spatial scanning improved, the more their reasoning improved; Table S10). Thus, ΔEFT was a strong competitor to neural change predictors, suitable for rigorous comparison of the value of the neural change variables relative to performance-based prediction. 5) Directly comparing behavioral performance change (ΔEFT) vs. neural change (ΔaIPS_EFT, ΔIPL_EFT) on the *same task* (i.e., EFT) enabled the most direct test of our hypothesis that neural change as a measure of learning would add value to (and perhaps even outperform) measures of learning that could be obtained without neuroimaging (i.e., change in behavioral performance).

The last (5th) point above has especially meaningful impacts on the interpretation of the findings. Recall that a goal of the present research was to assess the value of longitudinal neural change to

provide targets by which the efficacy of curricula can be evaluated (i.e., to support curriculum development). Comparing behavioral vs. neural change on the *same* task allows a clear interpretation that predictive value was derived from neural measurement over and above the value derived from behavioral measurement. We found that neural changes during EFT (ΔaIPS_EFT, ΔIPL_EFT) were even more important predictors of learning transfer than behavioral change on EFT (ΔEFT; see below). This means, for example, that students who showed relatively lesser improvement in EFT performance but relatively greater increase in activity in IPS and IPL (spatial cognition-implicated brain regions), were generally more likely to transfer learning beyond the spatial domain than students who showed greater improvement in performance on EFT but less increase in the recruitment of these spatial brain regions. Thus, the direct comparison of behavioral vs. neural change on EFT provides a clear demonstration of uniquely predictive neural variance (relative to behavioral measurement) for assessing the transferability of learning. This proof-of-principle demonstration points to the promise of real-world neural changes, particularly in spatial brain regions, for assessing whether curricula impart transferable learning.

Testing proof-of-principle for the predictive value of neural changes also involved comparing neural changes to several other performance-based assessments (scholastic performance, academic testing, cognitive testing, course grades), which were included in the prediction models to increase rigor and to more robustly reflect the range of performance-based assessments that are available without neuroimaging. Obtaining multiple performance-based assessments is generally easier than obtaining neuroimaging data, even in small-scale data collection for curriculum development, so it is important to test the value of neural changes against a range of behavioral measures considered together. Specifically, we included performance change in Spatial Habits-of-Mind (ΔSHOMI), another spatial outcome representing a key curricular learning goal of the Geospatial course. Performance change on mental rotation (ΔMRT) was included as an additional cognitive measure of spatial learning. Grade point average (GPA) prior to taking the Geospatial course was included as a standard measure of scholastic performance. Scores on the preliminary scholastic assessment test (PSAT) were included as standard measure of academic ability. Finally, course grades for the Geospatial course were included (for models involving only the Geospatial neuroimaging sample) as a performance-based academic measure of curriculum learning. Gender was also included as a predictor in all models.

Including both ΔaIPS_EFT and ΔIPL_EFT simultaneously in boosting analysis in the full neuroimaging sample, ΔIPL_EFT emerged as the most important predictor (accounting for 38% of predictor importance) and ΔaIPS_EFT as the second most important predictor (accounting for 28% of predictor importance; Table S33). Adding Geospatial course grades (Geospatial neuroimaging sample), ΔaIPS_EFT emerged as the most important predictor (accounting for 26% of predictor importance) and ΔIPL_EFT as the second most important predictor (accounting for 24% of predictor importance; Table S35). Analysis to select predictors to create the most informative standard model in the full neuroimaging sample (Table S34) resulted in the selection of three predictors: ΔIPL_EFT (accounting for 47% of predictor importance), PSAT (44%), and ΔaIPS_EFT (10%). Adding Geospatial course grades (Geospatial neuroimaging sample; Table S36) resulted in the selection of two variables: ΔIPL_EFT (accounting for 58% of predictor importance), and PSAT (42%).

Additional predictive ensemble modeling analyses were run to investigate the predictive contributions of the neural change variables individually (i.e., including only ΔaIPS_EFT alongside all performance-based assessments, and including only ΔIPL_EFT alongside all

performance-based assessments). In the ΔaIPS_EFT model, predictive ensemble model boosting indicated that ΔaIPS_EFT contributed most to accuracy in predicting ΔReasoning, accounting for 51% of predictor importance (Table S38). Adding Geospatial course grades to the model (Geospatial neuroimaging sample), ΔaIPS_EFT again emerged as the most important predictor, accounting for 33% of predictor importance (Table S39). The ΔIPL_EFT model closely paralleled the ΔaIPS_EFT model. ΔIPL_EFT contributed most to accuracy in predicting ΔReasoning, accounting for 56% of predictor importance (Table S41). Adding Geospatial course grades to the model (Geospatial neuroimaging sample), ΔIPL_EFT again emerged as the most important predictor, accounting for 35% of predictor importance (Table S43).

Additional comparisons of neural change vs. performance-based assessments

In addition to the prediction models, standard-regression models assessed whether the above-identified neural changes *comparatively* outperformed traditional performance-based assessments in predicting improvement on the verbal reasoning transfer task (ΔReasoning), and whether *adding* neural changes to performance-based measures yielded a significant increase beyond the predictive value (model fit) obtained from performance-based assessments alone. All performance-based assessments included in the predictive modeling analyses (above) were used in these analyses (ΔEFT, ΔSHOMI, ΔMRT, GPA, PSAT, Geospatial course grades). The neural changes predictors in these analyses were also the same ones included in the prediction modeling analyses, i.e., the longitudinal changes in IPS and IPL during EFT (ΔaIPS_EFT, ΔIPL_EFT) that emerged as Group-by-Time effects (indicating that they were associated with the spatial curriculum), and that predicted longitudinal improvement in Reasoning performance. As discussed above, neural change on the Reasoning task, though important for insights into mechanisms-of-change, was not considered a predictor because this measure was obtained during (not separately from) Reasoning.

We first determined which individual performance-based assessment was most predictive of ΔReasoning. Change in EFT performance (ΔEFT) was identified as the most predictive performance-based assessment in both the full neuroimaging sample (adjusted R^2=.13) and the Geospatial neuroimaging sample (adjusted R^2=.08). Of the neural change predictor variables, we found that ΔIPL_EFT yielded the best model fit for ΔReasoning in the full neuroimaging sample (R^2=.28), and that ΔaIPS_EFT yielded the best model fit in the Geospatial neuroimaging sample (R^2=.16). ΔaIPS_EFT alone showed significantly better fit for ΔReasoning than ΔEFT alone in both the full neuroimaging sample (adjusted R^2=.22; likelihood-ratio significance test of difference in model fit: χ^2=6.22, p<.001) and the Geospatial neuroimaging sample (adjusted R^2=.16; likelihood-ratio significance test of difference in model fit: χ^2=2.87, p<.001). Similarly, ΔIPL_EFT alone showed significantly better fit for ΔReasoning than ΔEFT alone in both the full neuroimaging sample (adjusted R^2=.28; likelihood significance ratio test of difference in model fit: χ^2=11.83, p<.001), and the Geospatial neuroimaging sample (adjusted R^2=.12; likelihood-ratio significance test of difference in model fit: χ^2=1.83, p<.001). This demonstrates not only that the neural predictors outperformed the strongest behavioral predictors, but more specifically that each of the neural change outcomes for EFT (i.e., spatial curriculum-related neural change during the spatial scanning task) outperformed the behavioral outcome from the same EFT measure (i.e., spatial-curriculum-related performance change in spatial scanning ability) in predicting transfer.

We next considered combinations of the performance-based assessments and the neural change variables. First, we identified the best-fitting performance-based assessment model. Among the 62 possible models comprising permutations of performance-based assessments in the full neuroimaging sample, the model that achieved the best fit for ΔReasoning included ΔEFT,

ΔMRT, and PSAT (adjusted R^2=.19). Among the 124 possible models comprising permutations of performance-based assessments in the neuroimaging sample of Geospatial students only, the best-fitting model included ΔEFT, PSAT, and Geospatial course grades (adjusted R^2=.09). We then compared these models to models combining the two neural change predictors (ΔaIPS_EFT, ΔIPL_EFT). The neural change-based model for the full neuroimaging sample yielded a model fit that was significantly better than the best-fitting performance-based assessment model (adjusted R^2=.30; likelihood-ratio significance test of difference in model fit: χ^2=7.47, *p*=.006; Table S45). In the Geospatial only neuroimaging sample, the neural change-based model also yielded a fit that was significantly better than the best-fitting performance-based assessment model (adjusted R^2=.19; likelihood-ratio significance test of difference in model fit: χ^2=2.21, *p*<.001; Table S46). Even when considered individually, ΔaIPS_EFT and ΔIPL_EFT each yielded nominally higher model fits for ΔReasoning than the best-fitting combination of performance-based assessments in both the full neuroimaging sample (adjusted R^2=.22 and adjusted R^2=.28, respectively) and the Geospatial neuroimaging sample (adjusted R^2=.19 and adjusted R^2=.12, respectively), though these differences did not reach significance.

Next, we assessed whether *adding* the neural change predictors to the best-fitting combination of performance-based assessments improved the fit of these performance-based assessment models for ΔReasoning. Each of the neural change variables (ΔaIPS_EFT and ΔIPL_EFT) was included individually and together. Analysis in the full neuroimaging sample showed that the fit (R^2 values) of the best-fitting combination of performance-based assessments was significantly improved by the addition of each neural change variable separately and by the addition of both neural variables together (all *p*<.001). Tables S47-49 display comparisons of the performance-based assessment model that achieved the best fit for ΔReasoning in the full neuroimaging sample (ΔEFT, ΔMRT, and PSAT) vs. the same model with the addition of each neural change separately, and the addition of both neural changes together. Tables S50-52 display the corresponding set of model comparisons in the Geospatial neuroimaging sample (with Geospatial course grades included). Within the Geospatial neuroimaging sample, ΔaIPS_EFT was the only neural change to significantly improve the best-fitting performance-based assessment model when added individually (adjusted R^2=.21; likelihood-ratio significance test of difference in model fit: χ^2=5.35, *p*=.021; Table S50), though the addition of ΔIPL_EFT resulted in a nominally improved fit (adjusted R^2=.16; likelihood-ratio significance test of difference in model fit: χ^2=3.37, *p*=.066; Table S52). Adding the combination of ΔaIPS_EFT and ΔIPL_EFT also improved model fit of the best-fitting performance-based assessment model in the Geospatial only neuroimaging sample (adjusted R^2=.20; likelihood-ratio significance test of difference in model fit: χ^2=6.29, *p*=.046; Table S52).

A notable outcome of these analyses was that, for both the full neuroimaging sample and the Geospatial only neuroimaging sample, the model achieving the best overall fit included both neural changes and performance-based assessments (rather than neural changes alone). In the full neuroimaging sample, the model that achieved the best fit included ΔEFT, ΔMRT, PSAT, ΔIPL_EFT, and ΔaIPS_EFT (R^2=.39; Table S53). In the full neuroimaging sample, this model achieved significantly stronger model fit than the best neural change-based model (ΔIPL_EFT and ΔaIPS_EFT; likelihood-ratio significance test of difference in model fit: χ^2=11.79, *p*=.008) and the best performance-based model (ΔEFT, ΔMRT, and PSAT; likelihood-ratio significance test of difference in model fit: χ^2=19.59, *p*<.001). In the Geospatial neuroimaging sample, the model that achieved the best fit included ΔEFT, PSAT, Geospatial course grades, and ΔaIPS_EFT (R^2=.21; Table S54). However, in the Geospatial-only neuroimaging sample, this model was only nominally higher but did not show significantly stronger model fit (both likelihood-ratio

significance test of difference in model fit p>.20) than the best neural change-based model (ΔIPL_EFT and ΔaIPS_EFT) and the best performance-based model (ΔEFT, PSAT, and Geospatial course grades). Overall, these results suggest that neurally-informed approaches to curriculum development are likely to achieve optimal prediction of transferable by employing measures of neural change to bolster (but not replace) traditional assessments.

Post-hoc exploratory analyses investigated whether the neural changes that were associated with improved reasoning performance were related to each other. As described above, the change in SpatialMap-to-DLPFC connectivity was the only longitudinal neural change during the Reasoning task that was significantly associated with improved reasoning performance. As also described above, changes in two PPC regions (ΔaIPS_EFT and ΔIPL_EFT) predicted improved reasoning performance. We thus sought to test whether ΔaIPS_EFT and ΔIPL_EFT were related to change in SpatialMap-to-DLPFC connectivity during Reasoning. The ΔaIPS_EFT and ΔIPL_EFT clusters spatially overlapped (in IPS and IPL) with the areas of increased connectivity to DLPFC during Reasoning, and both ΔaIPS_EFT (β = .414, <.001; Table S55) and ΔIPL_EFT (β = .262, p=.038; Table S56) predicted the increase in SpatialMap-to-DLPFC connectivity. This post-hoc finding provides an additional indication that spatial education may influence IPS and IPL function in ways that support both spatial scanning and verbal reasoning (putatively because the same spatial attentional resources that support spatial scanning also contribute to verbal reasoning in conjunction with DLPFC). Relatedly, and also post-hoc, we tested whether the longitudinal changes in aIPS activity that we observed separately in EFT and Reasoning were associated with each other. Indeed, the longitudinal change in aIPS during EFT (ΔaIPS_EFT) predicted the longitudinal change in aIPS during Reasoning (β = .340, p = .006; Table S57).

Individual Differences and Inter-Subject Variability

Data visualizations are provided for changes in performance and brain activity at the individual subject level (Figs. S16-22). Within the Geospatial group, 77% of the students showed increased reasoning performance and the average change was .07 more problems solved per second (Median=.05, SD=.11). In the control group, 68% of the students showed increased performance and the average change was .03 more problems solved per second (Median=.03, SD=.08). In addition, Cook's distance analysis examining the effect of the Geospatial course on change in reasoning performance found no significant outliers (all Cook's distance < 0.2). Within the Geospatial group, 80% of the students showed increased performance and the average change was .04 more problems solved per second (Median=.04, SD=.05). In the control group, 70% of the students showed increased performance and the average change was .02 more problems solved per second (Median=.02, SD=.04). In addition, Cook's distance analysis found no significant outliers (all Cook's distance < 0.2).

Individual effect sizes (regression slope coefficients) (*99*), were calculated for the effect of time (i.e. change) on behavioral and neural variables for both spatial scanning and reasoning, separately for the two groups (Geospatial and the Control). Data visualizations can be found in Figs. S16-20. Scatterplots were also generated for the correlations between spatial scanning-related neural changes (e.g., change in aIPS_EFT and IPL_EFT) and changes in reasoning performance, separately for each Group (Geospatial and Control). Data visualizations can be found in Figs. S21-22. In addition, Cook's distance analysis on the effect of the Geospatial course on all neural outcome variables found no significant outliers (all cook's distance < 0.2).

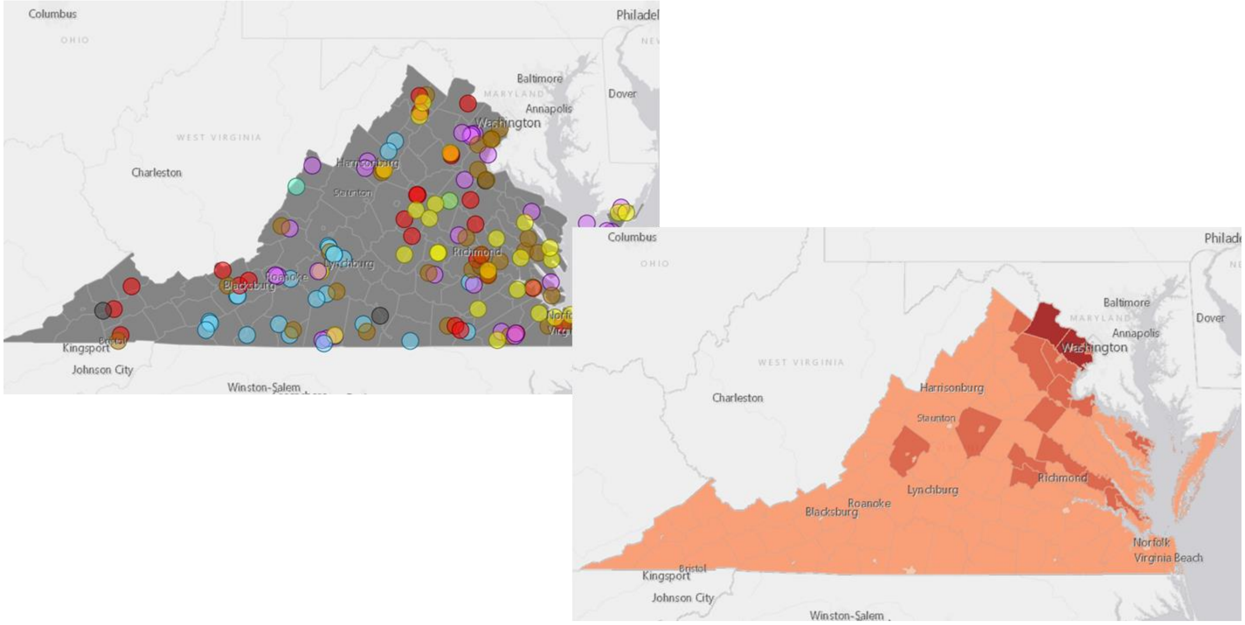

Fig. S1.
Example of maps used in the Geospatial Semester. These maps were created by a student for their final project, which investigated the location of power plants and the power usage across the state of Virginia.

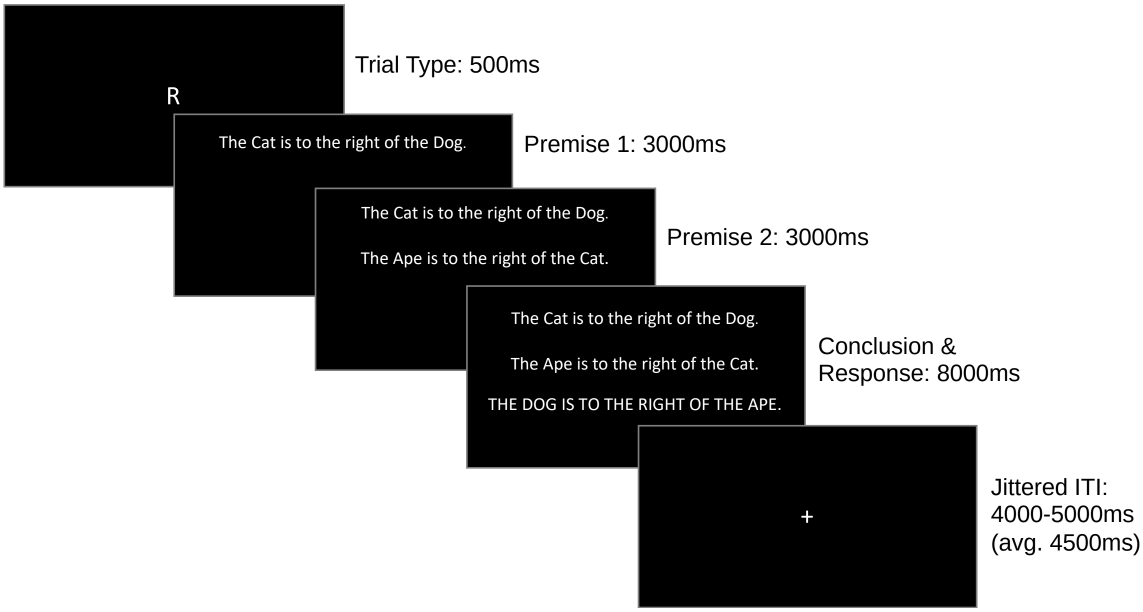

Fig. S2.
Task design of the Reasoning task.

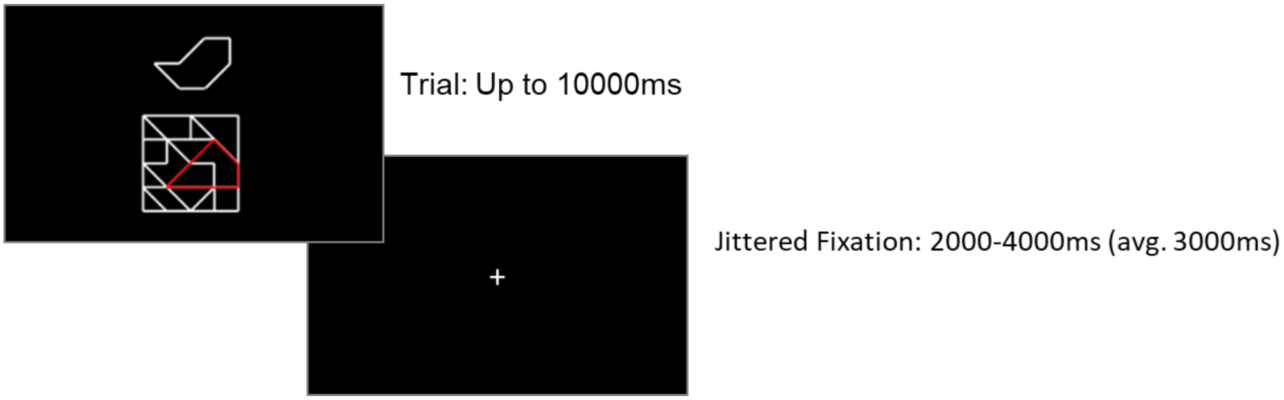

Fig. S3.
Task design of the Embedded Figures Task (EFT).

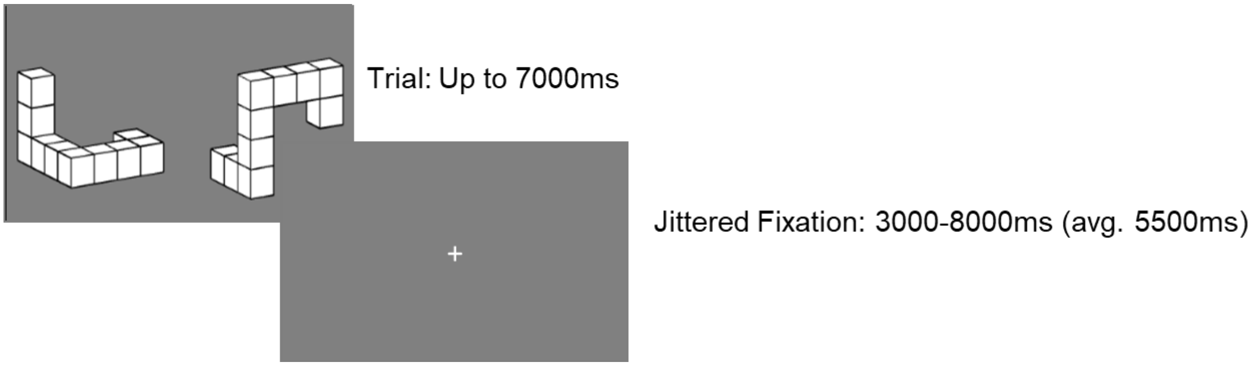

Fig. S4.
Task design of the Mental Rotation Task (MRT).

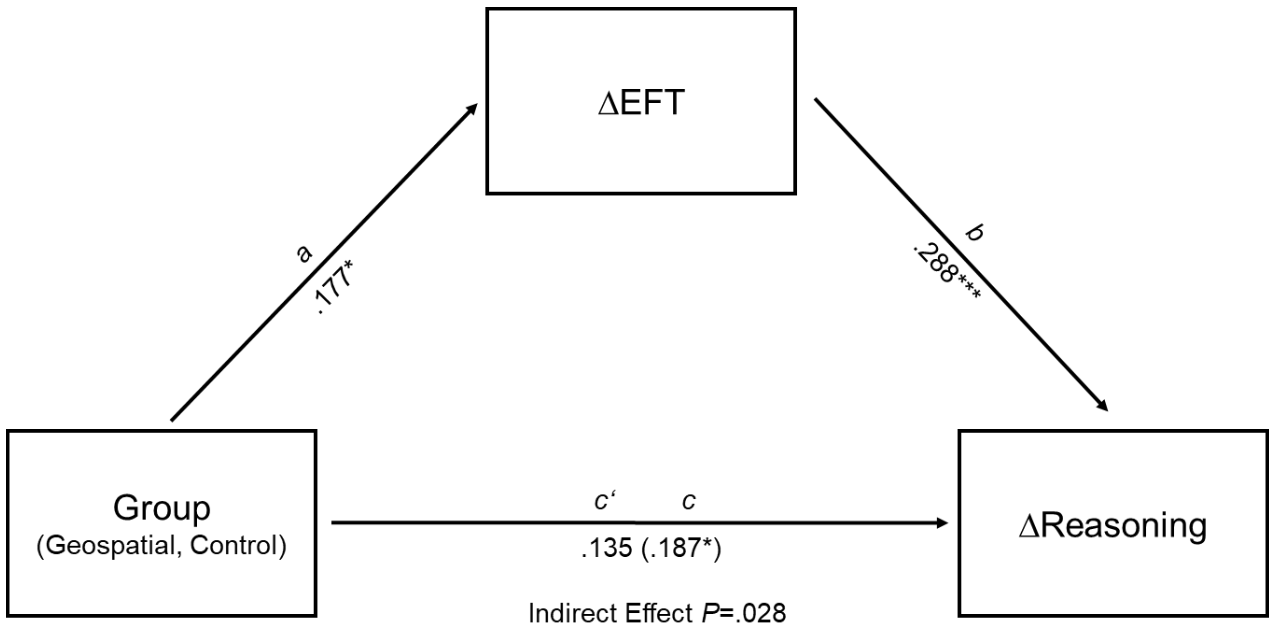

Fig. S5.
Mediation model with Group (Geospatial=1, Control=0) modeled as the independent variable, ΔEFT modeled as the mediator, ΔReasoning modeled as the dependent variable, covarying GPA, PSAT, and Gender. Improved spatial scanning performance (ΔEFT) significantly mediated the association of the Geospatial curriculum with improved reasoning performance (considering all Reasoning trials). Paths displayed with standardized regression coefficients. *$p<.05$, **$p<.01$, ***$p<.001$.

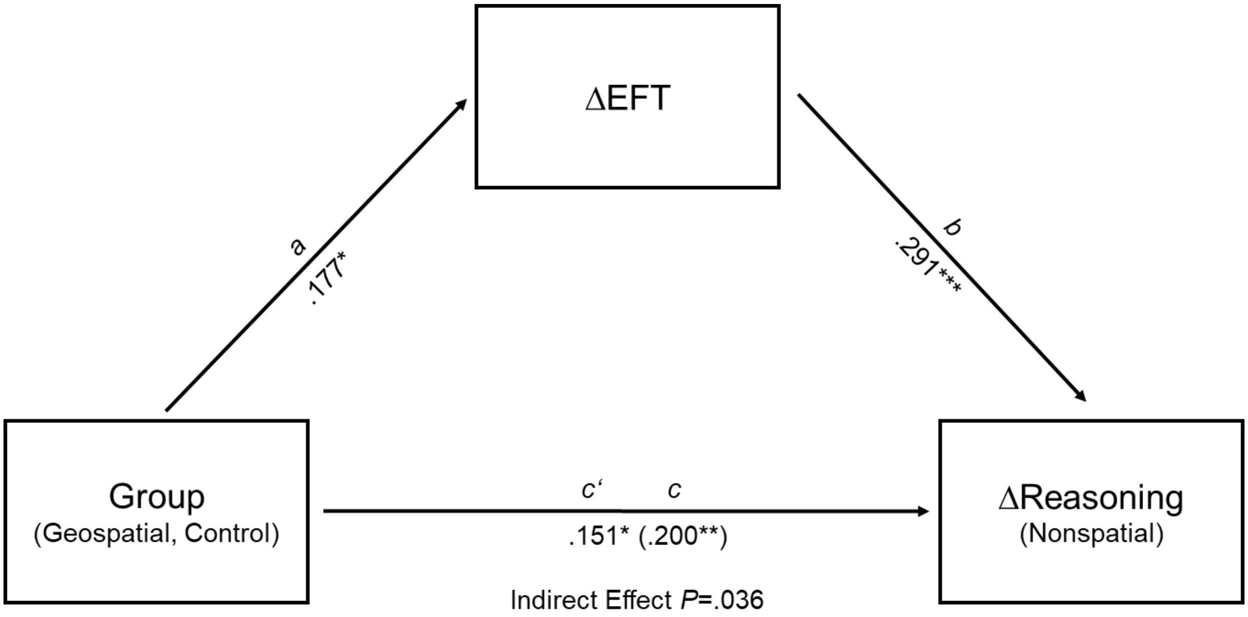

Fig. S6.
Mediation model with Group (Geospatial=1, Control=0) modeled as the independent variable, ΔEFT modeled as the mediator, ΔReasoning for trials involving nonspatial relations (e.g., “better”) modeled as the dependent variable, covarying GPA, PSAT, and Gender. Improved spatial scanning performance (ΔEFT) significantly mediated the association of the Geospatial curriculum with improved reasoning performance (considering nonspatial Reasoning trials only). Paths displayed with standardized regression coefficients. *p<.05, **p<.01, ***p<.001.

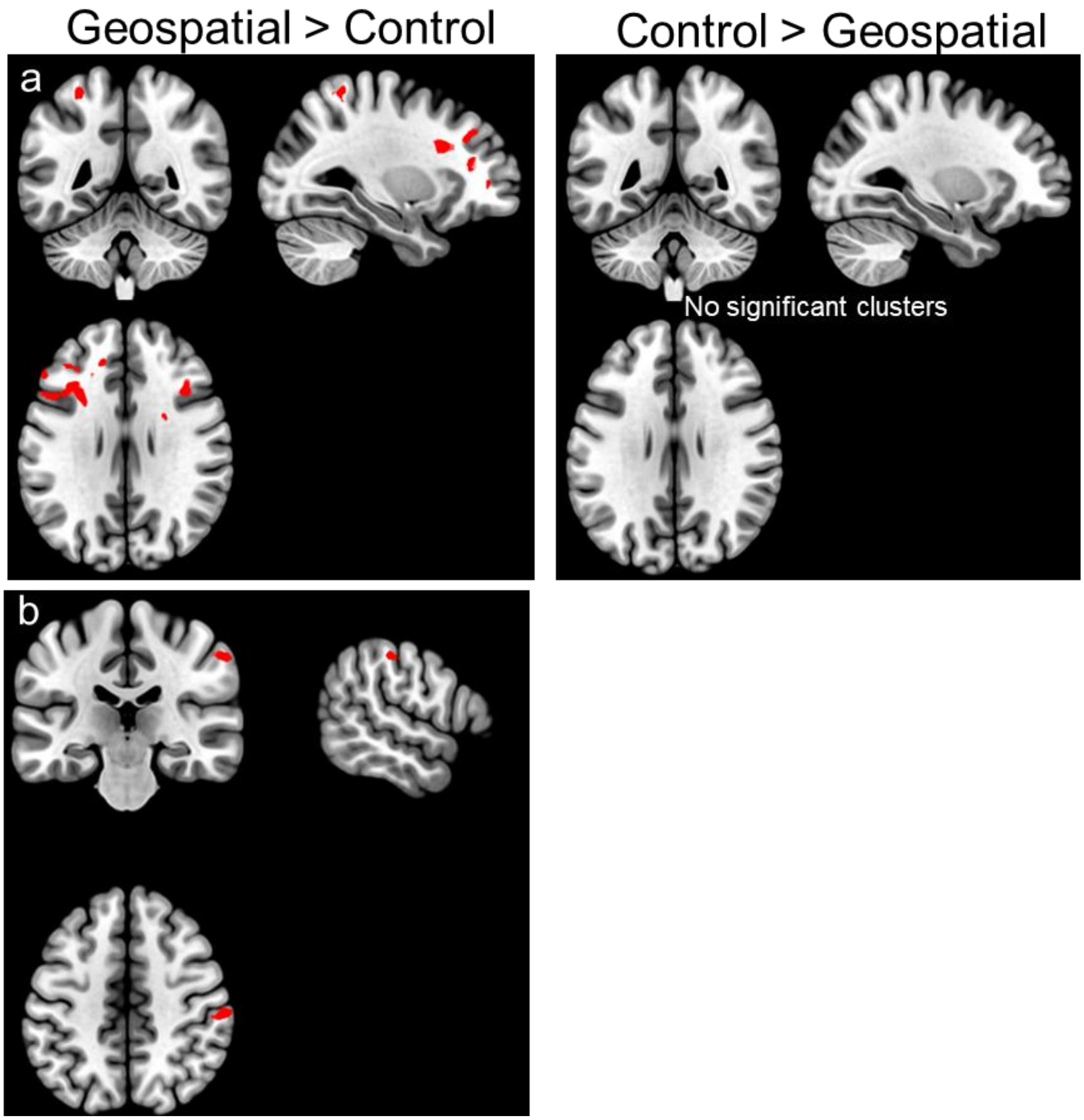

Fig. S7.
Clusters of longitudinal increases in activity (red) during EFT from the Geospatial>Control and Control>Geospatial interaction terms of the whole-brain Group(Geospatial, Control) X Time(T1, T2) ANOVA (i.e. brain regions showing differential increases in activity for Geospatial vs. Control from T1 to T2). Greater increase in activity was observed for Geospatial students in left anterior intra-parietal sulcus (**Fig. S7a**) and right inferior parietal lobule(**Fig. S7b**); both regions are strongly implicated in spatial cognition(*45*).

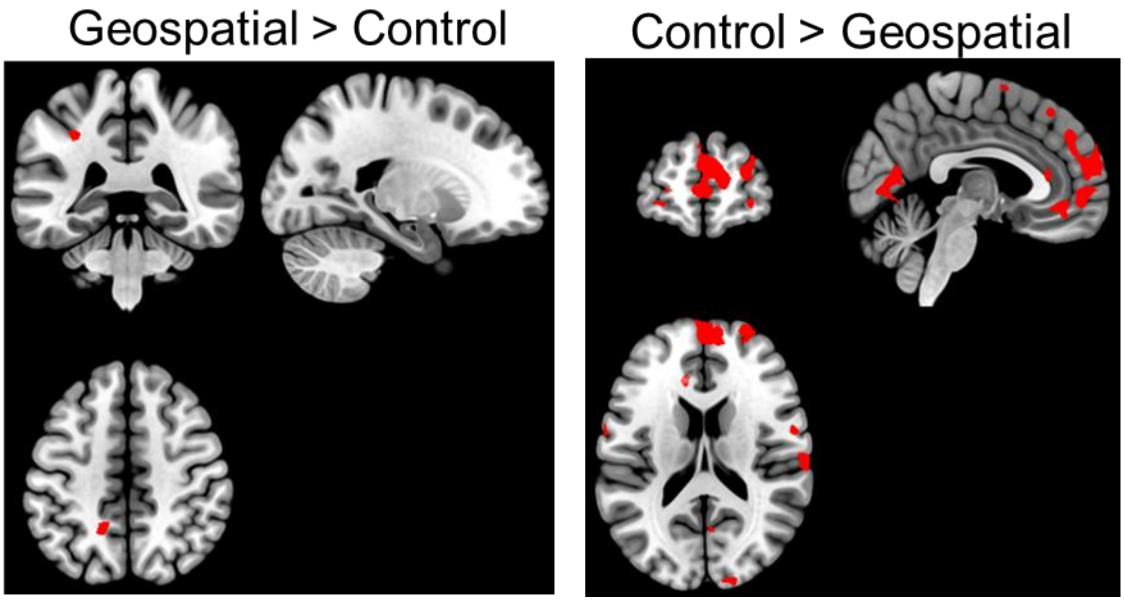

Fig. S8.
Clusters of longitudinal increases in activity (red) during Reasoning from the Geospatial>Control and Control>Geospatial interaction terms of the whole-brain Group(Geospatial, Control) X Time(T1, T2) ANOVA (i.e. brain regions showing differential increases in activity for Geospatial vs. Control from T1 to T2). Greater increase in activity within SpatialMap was observed for Geospatial students in two clusters: left anterior intra-parietal sulcus and a cluster extending from left anterior intra-parietal sulcus into superior parietal lobe. No decreases in activity (i.e., Control > Geospatial) fell within SpatialMap.

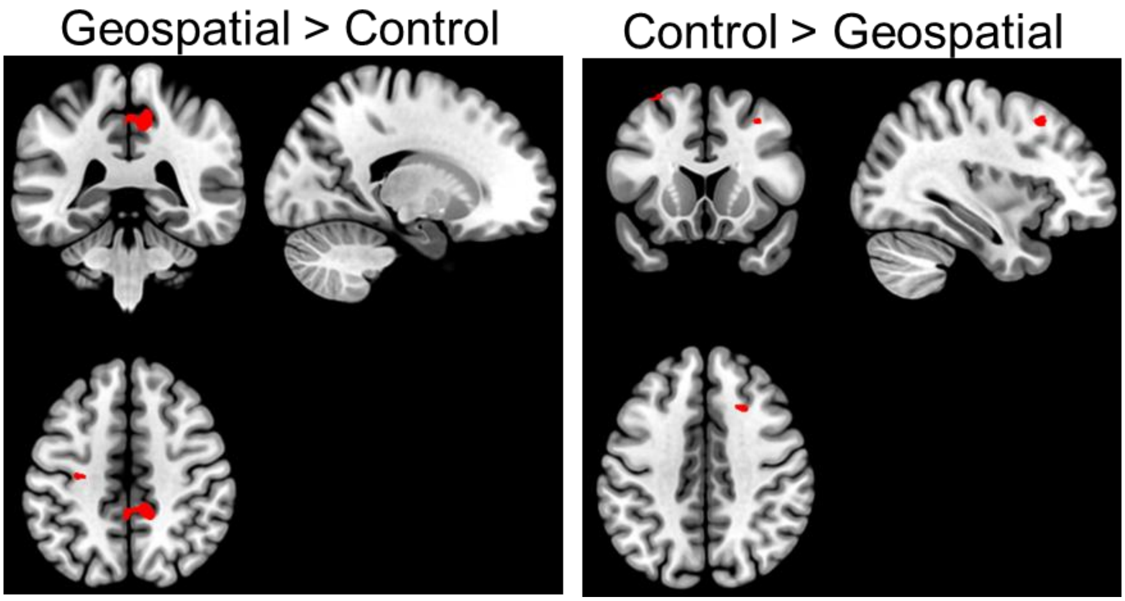

Fig. S9.
Clusters of longitudinal increases in activity (red) during MRT from the Geospatial>Control and Control>Geospatial interaction terms of the whole-brain Group(Geospatial, Control) X Time(T1, T2) ANOVA (i.e. brain regions showing differential increases in activity for Geospatial vs. Control from T1 to T2).

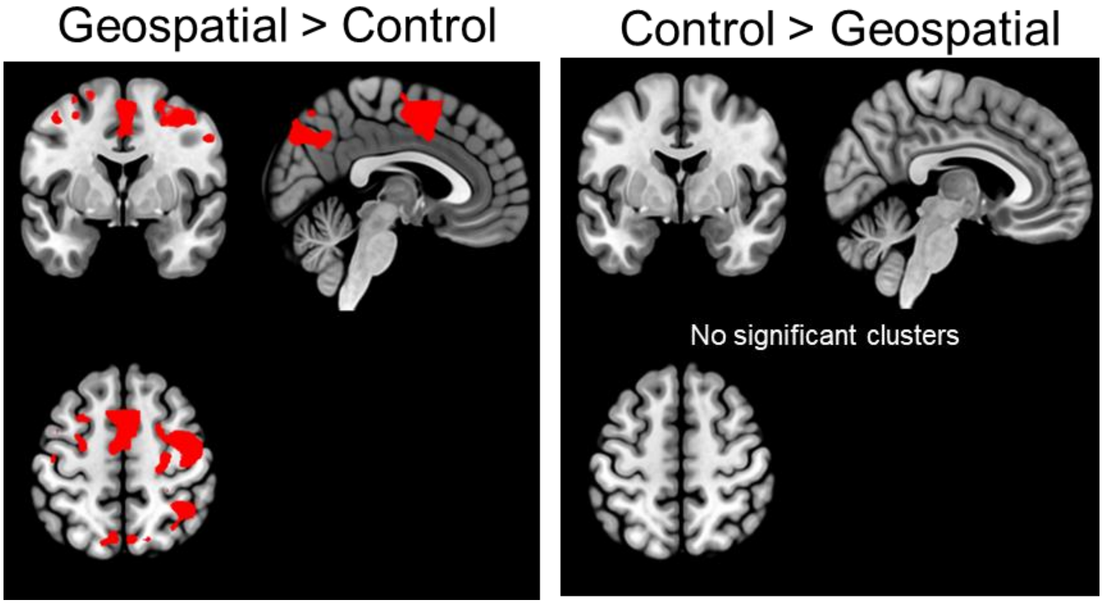

Fig. S10.
Clusters of longitudinal increases in connectivity from regions within the SpatialMap ROI to the left DLPFC seed region (red) during Reasoning from the Geospatial>Control and Control>Geospatial interaction terms of the whole-brain Group(Geospatial, Control) X Time(T1, T2) ANOVA (i.e. brain regions showing differential increases in connectivity to PFC for Geospatial vs. Control from T1 to T2).Greater increase in connectivity from SpatialMap to left DLPFC was observed for Geospatial participants in bilateral posterior parietal cortex (including both anterior intra-parietal sulcus and inferior parietal lobe) and premotor cortex.

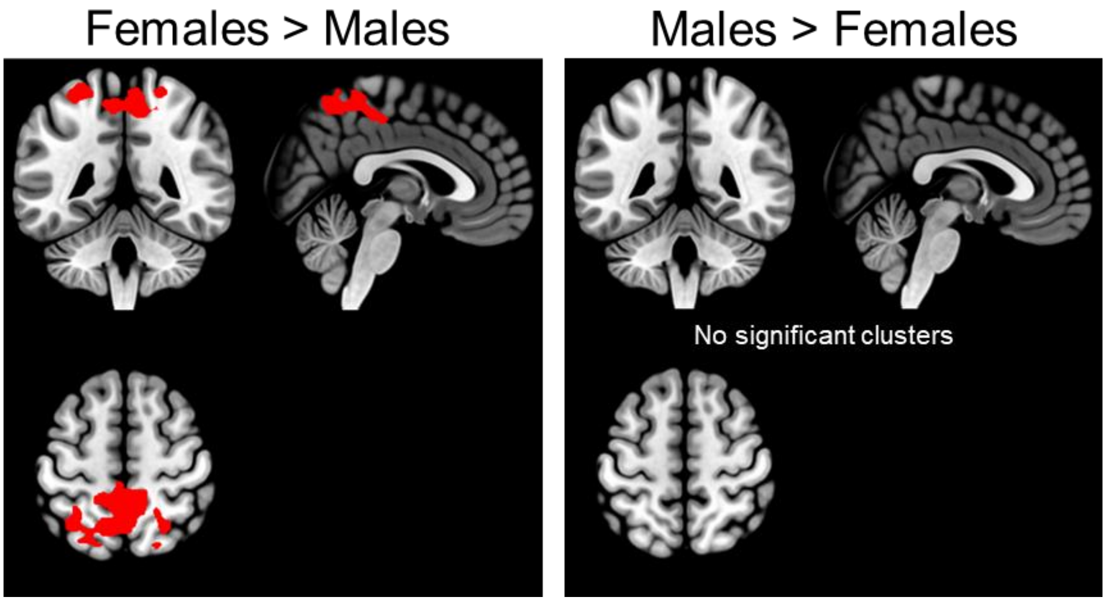

Fig. S11.
Clusters of longitudinal increases in connectivity from regions within the SpatialMap ROI to the left DLPFC seed region (red) during Reasoning for Female>Male and Male>Female contrasts in the from the Geospatial>Control and Control>Geospatial interaction terms of the whole-brain Group(Geospatial, Control) X Time(T1, T2) ANOVA (i.e. brain regions where the difference in the increase in SpatialMap-to-DLPFC connectivity for female vs. male students was greater in the Geospatial group than in the Control group). Greater increase in connectivity from SpatialMap to PFC was observed for female Geospatial participants in bilateral superior parietal lobe.

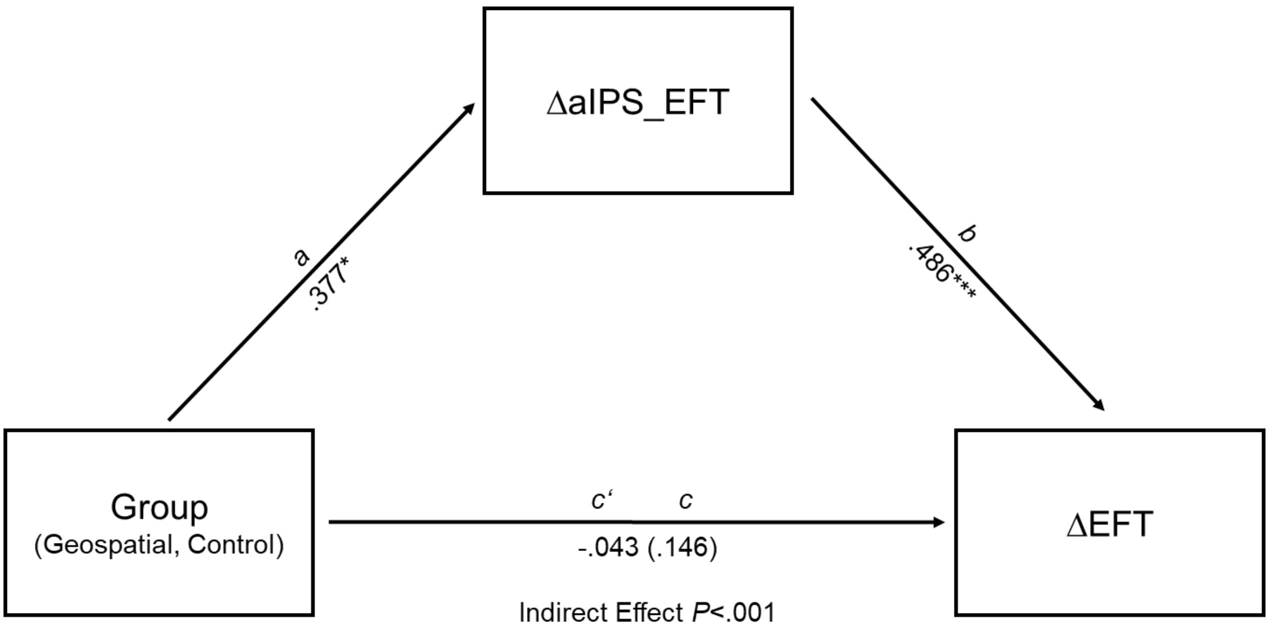

Fig. S12.
Mediation model with Group (Geospatial=1, Control=0) modeled as the independent variable, ΔaIPS_EFT modeled as the mediator, and ΔEFT modeled as the dependent variable, covarying GPA, PSAT, and Gender. Longitudinal increase in aIPS activity during EFT significantly mediated the association of the Geospatial curriculum with improved EFT performance. Paths displayed with standardized regression coefficients. *p<.05, **p<.01, ***p<.001.

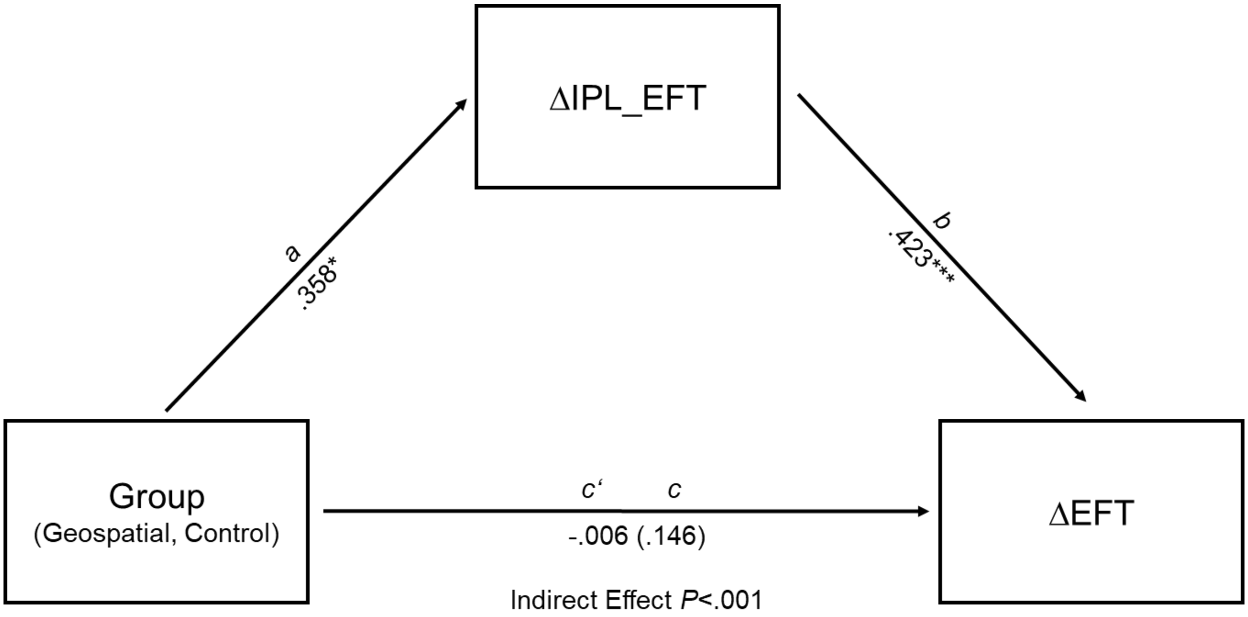

Fig. S13.
Mediation model with Group (Geospatial=1, Control=0) modeled as the independent variable, ΔIPL_EFT modeled as the mediator, and ΔEFT modeled as the dependent variable, covarying GPA, PSAT, and Gender. Longitudinal increase in IPL activity during EFT significantly mediated the association of the Geospatial curriculum with improved EFT performance. Paths displayed with standardized regression coefficients. *p<.05, **p<.01, ***p<.001.

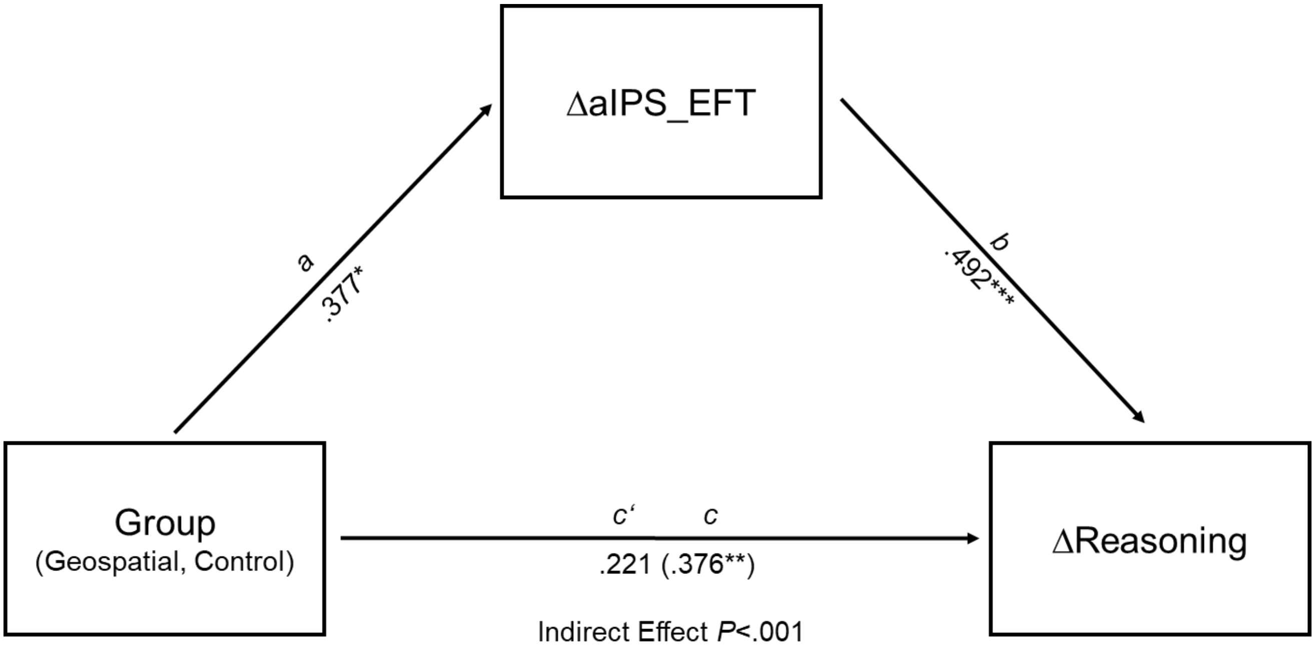

Fig. S14.
Mediation model with Group (Geospatial=1, Control=0) modeled as the independent variable, ΔaIPS_EFT modeled as the mediator, and ΔReasoning modeled as the dependent variable, covarying GPA, PSAT, and Gender. Longitudinal increase in aIPS activity during EFT significantly mediated the association of the Geospatial curriculum with improved reasoning performance. Paths displayed with standardized regression coefficients. *p<.05, **p<.01, ***p<.001.

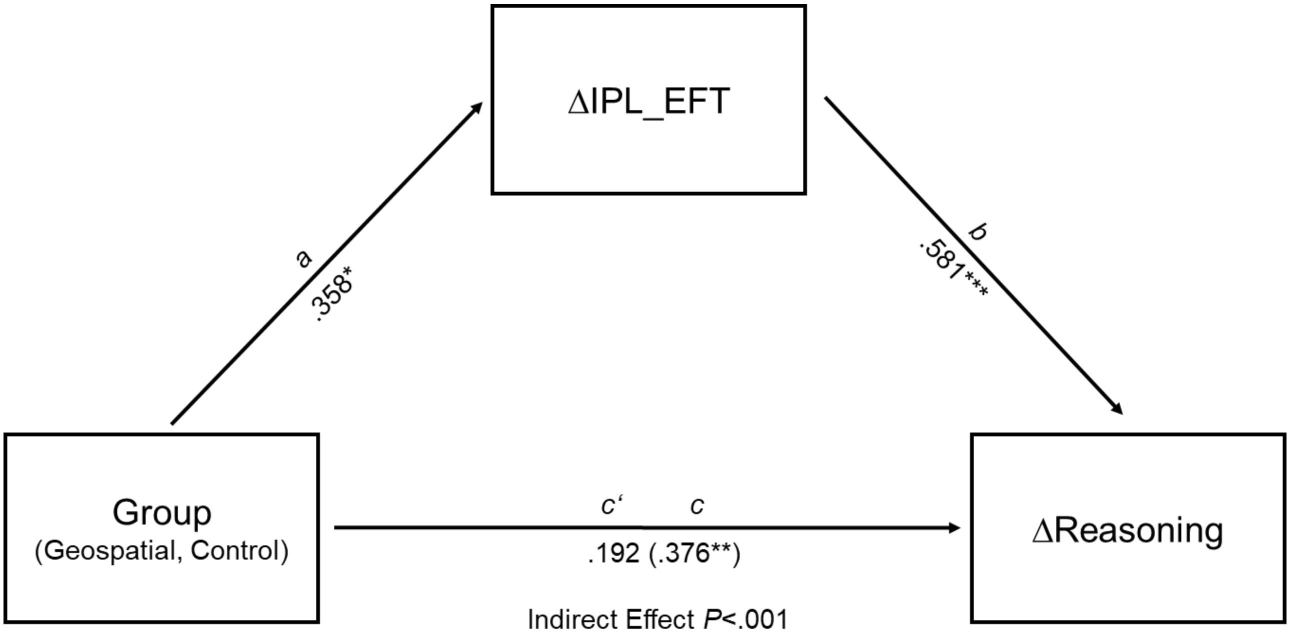

Fig. S15.
Mediation model with Group (Geospatial=1, Control=0) modeled as the independent variable, ΔIPL_EFT modeled as the mediator, and ΔReasoning modeled as the dependent variable, covarying GPA, PSAT, and Gender. Longitudinal increase in IPL activity during EFT significantly mediated the association of the Geospatial curriculum with improved reasoning performance. Paths displayed with standardized regression coefficients. *p<.05, **p<.01, ***p<.001.

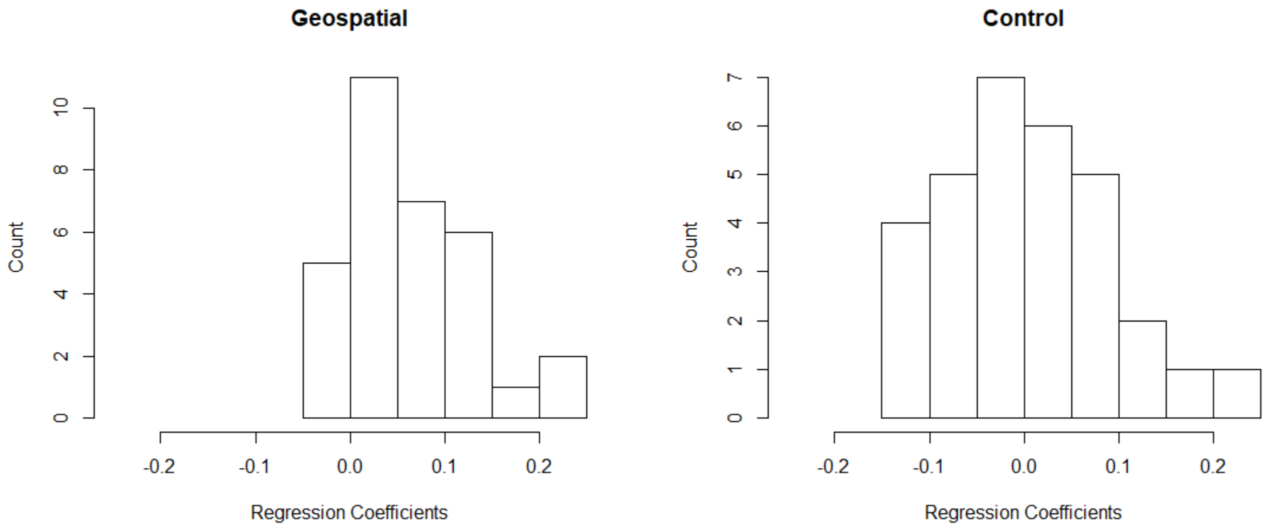

Fig. S16
Histograms of the individual regression slope coefficients for the effect of time on reasoning performance in the Geospatial and Control groups.

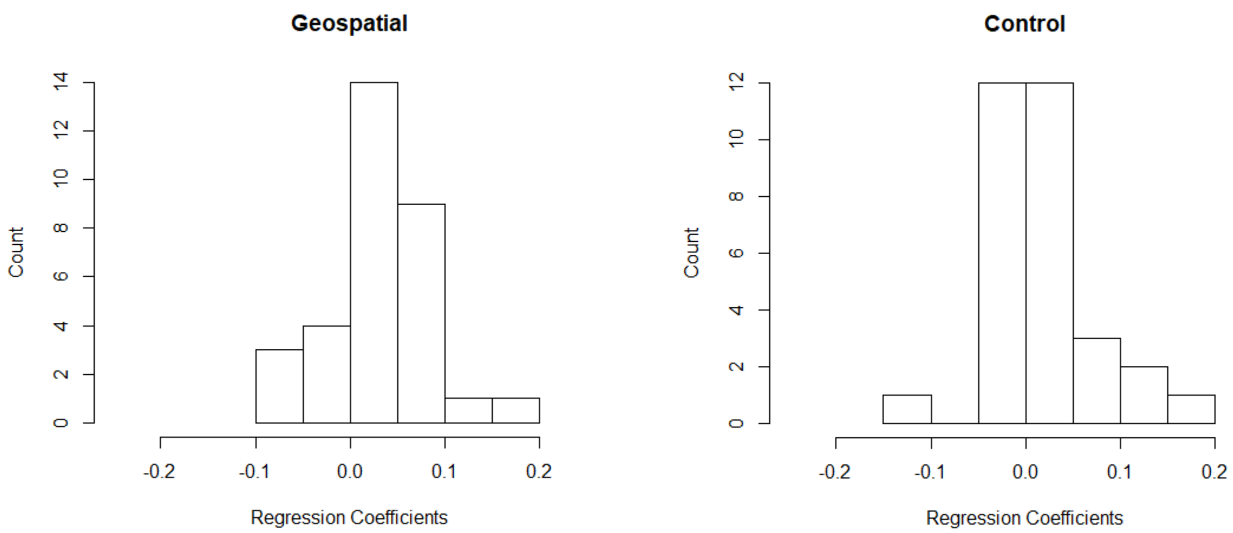

Fig. S17
Histograms of the individual regression slope coefficients for the effect of time on spatial scanning performance in the Geospatial and Control groups.

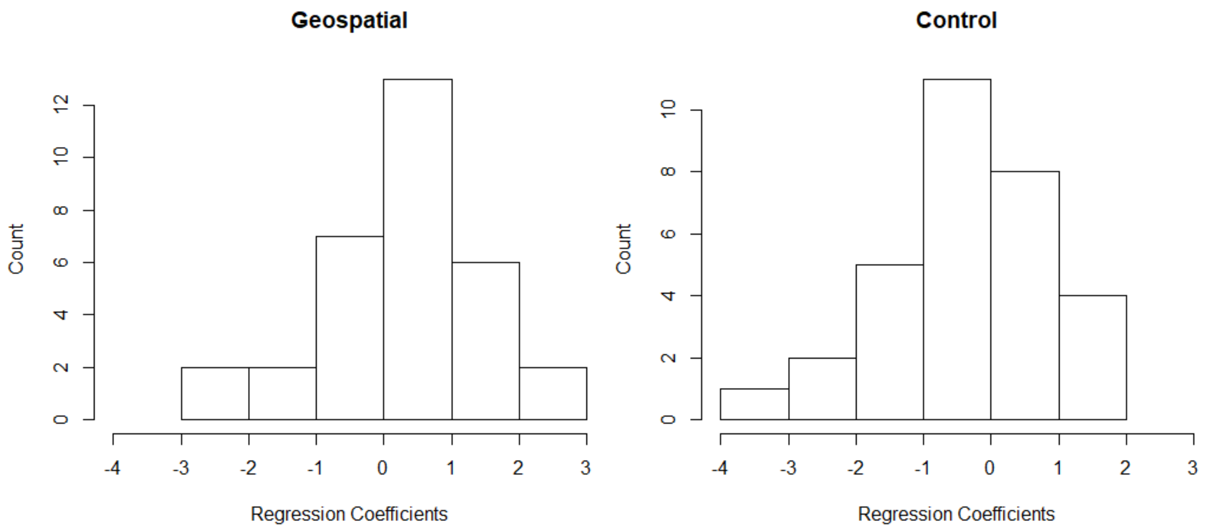

Fig. S18
Histograms of the individual regression slope coefficients for the effect of time on aIPS_Reasoning activity in the Geospatial and Control groups.

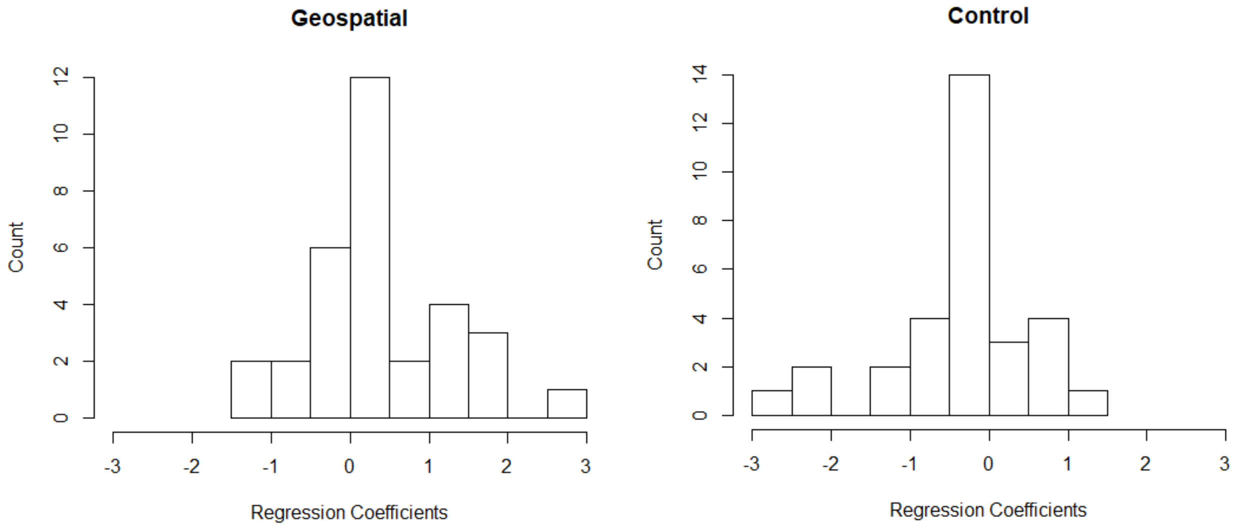

970

Fig. S19

Histograms of the individual regression slope coefficients for the effect of time on aIPS_EFT activity in the Geospatial and Control groups.

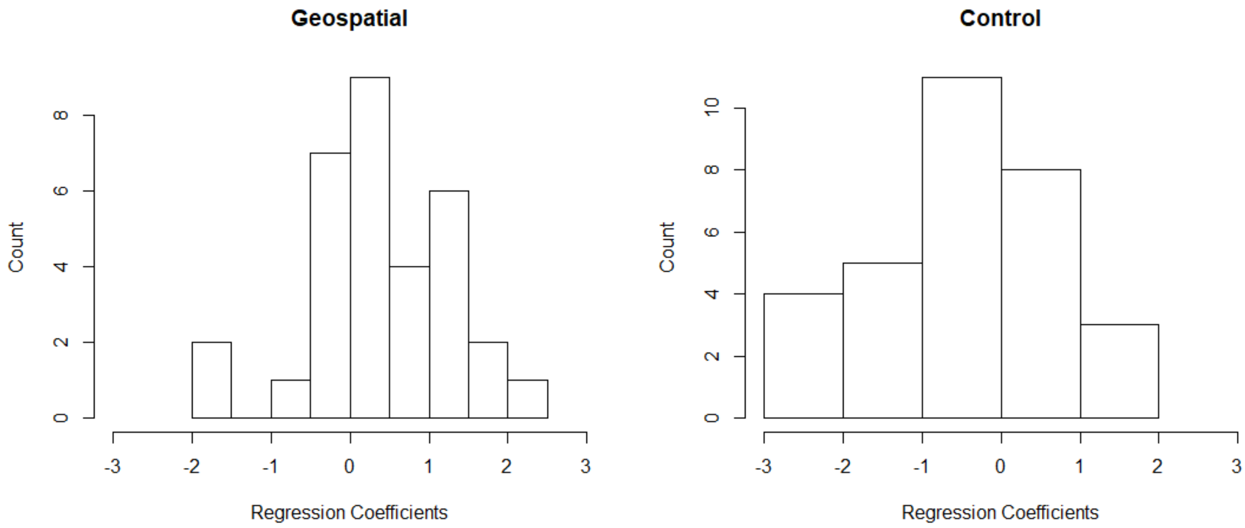

Fig. S20
Histograms of the individual regression slope coefficients for the effect of time on IPL_EFT activity in the Geospatial and Control groups.

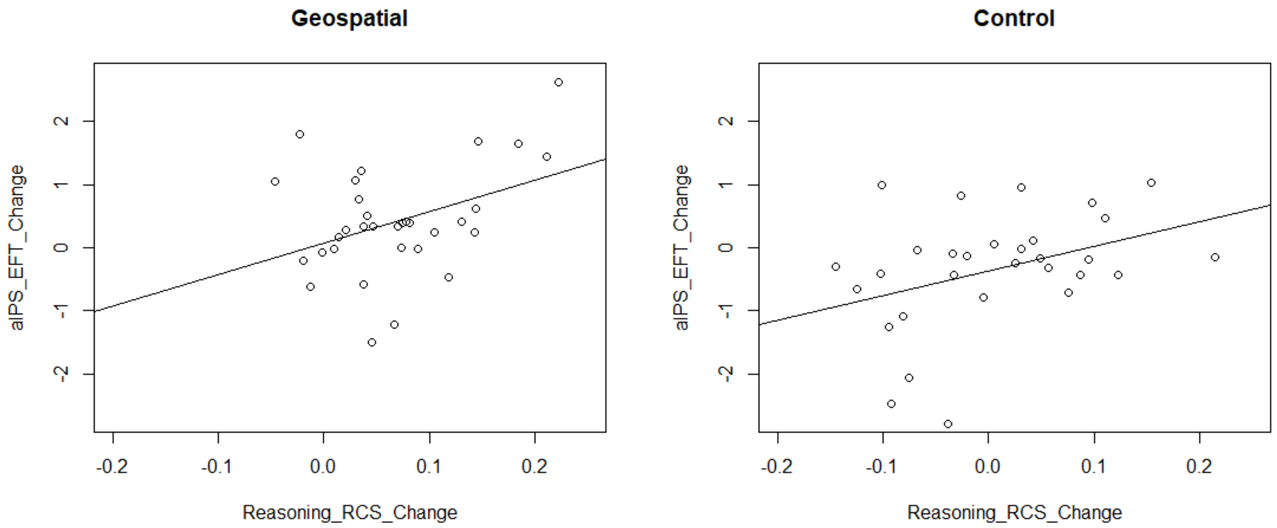

Fig. S21
Scatterplots of the correlation between aIPS_EFT and change in reasoning performance for the Geospatial and Control group.

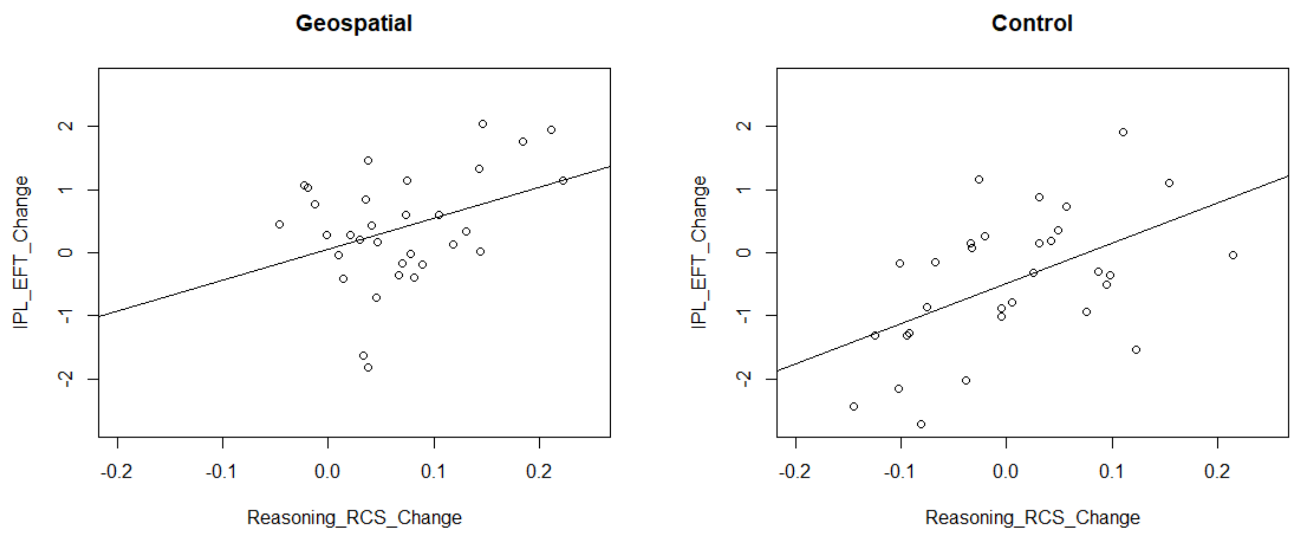

Fig. S22
Scatterplots of the correlation between IPL_EFT and change in reasoning performance for the Geospatial and Control groups.

	Total (*N*=182)			Geospatial (*N*=77)		Control (*N*=105)		Difference (t-test)	
Variable	Range	M	SD	M	SD	M	SD	*t*	*p*
Age	15-18	16.64	.51	16.66	.50	16.63	.52	.44	.66
Gender	48.4% Female, 51.6% Male			41.6%Female 58.4% Male		53.3%Female 46.7% Male		1.58	.12
Race	82.3% White, 17.7% Other			83.1% White, 16.9% Other		81.7% White, 18.3% Other		-1.04	.30
Mother Graduated College	44.5% Yes 55.5% No			38.9% Yes 61.1% No		48.6% Yes 51.4% No		-1.29	.20
Competent with GIS	13.8% Yes 86.2% No			15.6% Yes 84.4% No		12.5% Yes 87.5% No		.58	.56
Taken Course with Geospatial Teacher	21.9% Yes 78.1% No			28.6% Yes 71.4% No		17.8% Yes 82.2% No		1.03	.31
Hours Spent Playing Video Games per week	0-72	5.39	8.78	5.82	9.97	5.09	7.82	.52	.60
PSAT	106-225	163.26	22.93	163.70	23.51	162.93	22.61	.21	.83
Overall GPA	1.43-4.22	3.60	.43	3.59	.45	3.60	.41	-.11	.91
Math GPA	1.06-4.30	3.31	.64	3.35	.57	3.28	.69	.78	.44
Science GPA	0.68-4.30	3.46	.60	3.48	.63	3.44	.58	.53	.60
Paper Folding	2-20	11.53	3.79	11.00	3.70	11.91	3.82	-1.62	.11
SHOMI (Pre-test)	67-135	102.74	12.05	103.14	11.32	102.44	12.61	.39	.70
CAQ Spatial	16-70	42.89	10.30	42.43	10.63	43.22	10.09	-.50	.62

Table S1.
Descriptive statistics for all background variables used in propensity score matching, reported for full sample, and separately for Geospatial and Control samples.

Predictor	*df*	Sum of Squares	Mean Square	*F*	*p*	np^2
(Intercept)	1	58.46	58.46	5.38	.022	.033
Gender	1	52.93	52.93	4.87	.029	.030
GPA	1	27.96	27.96	2.57	.111	.016
PSAT	1	6.27	6.27	.577	.449	.004
Group	1	5.45	5.45	.501	.480	.003
Time	1	2.59	2.59	.598	.440	.004
Group x Time	1	23.31	23.31	5.38	.022	.033
Gender x Group x Time	1	1.43	1.43	.329	.567	.002
Error	157	679.96	4.33			

Table S2.
Group (Geospatial, Control) by Time (T1, T2) ANOVA results for EFT, covarying Gender, GPA, and PSAT. RCS values were converted from correct responses per second to correct responses per minute (i.e., multiplied by a constant of 60) for ease of display.

Predictor	*df*	Sum of Squares	Mean Square	*F*	*p*	np^2
(Intercept)	1	313.19	313.19	3.98	.048	.025
Gender	1	135.05	135.03	1.71	.192	.011
GPA	1	6.94	6.94	.088	.767	.001
PSAT	1	3320.79	3320.79	42.16	<.001	.211
Group	1	.785	.785	.010	.921	0
Time	1	26.86	26.86	1.46	.229	.009
Group x Time	1	103.03	103.3	5.60	.019	.034
Gender x Group x Time	1	5.44	5.44	.295	.588	.002
Error	158	2909.15	18.41			

Table S3.

Group (Geospatial, Control) by Time (T1, T2) ANOVA results for Reasoning, covarying Gender, GPA and PSAT. RCS values were converted from correct responses per second to correct responses per minute (i.e., multiplied by a constant of 60) for ease of display.

Predictor	*df*	Sum of Squares	Mean Square	*F*	*p*	np^2
(Intercept)	1	294.20	294.20	2.61	.108	.016
Gender	1	133.79	133.79	1.19	.277	.007
GPA	1	73.71	73.71	.656	.419	.004
PSAT	1	4664.89	4664.89	41.49	<.001	.208
Group	1	25.70	25.70	.229	.633	.001
Time	1	60.04	60.04	1.75	.188	.011
Group x Time	1	228.66	228.66	6.65	.011	.040
Gender x Group x Time	1	2.87	2.87	.084	.773	.001
Error	158	5434.51	34.49			

Table S4.
Group (Geospatial, Control) by Time (T1, T2) ANOVA results for Reasoning trials involving nonspatial relations (e.g., “better”), covarying Gender, GPA and PSAT. RCS values were converted from correct responses per second to correct responses per minute (i.e., multiplied by a constant of 60) for ease of display.

Predictor	*df*	Sum of Squares	Mean Square	*F*	*p*	np^2
(Intercept)	1	265.31	265.31	14.96	<.001	.087
Gender	1	239.97	239.97	13.53	<.001	.079
GPA	1	3.73	3.73	.210	.647	.001
PSAT	1	27.48	27.48	1.55	.215	.010
Group	1	1.49	1.49	.084	.772	.001
Time	1	2.34	2.34	.375	.541	.002
Group x Time	1	8.73	8.73	1.40	.239	.009
Gender x Group x Time	1	.229	.229	.037	.849	0
Error	158	980.62	6.25			

Table S5.
Group (Geospatial, Control) by Time (T1, T2) ANOVA results for MRT, covarying Gender, GPA, and PSAT. RCS values were converted from correct responses per second to correct responses per minute (i.e., multiplied by a constant of 60) for ease of display.

Predictor	*df*	Sum of Squares	Mean Square	*F*	*p*	np^2
(Intercept)	1	36290.67	36290.67	150.67	<.001	.491
Gender	1	2504.48	2504.48	10.39	.002	.062
GPA	1	130.35	130.35	.541	.463	.003
PSAT	1	22.65	22.65	.094	.760	.001
Group	1	594.19	594.19	2.47	.118	.016
Time	1	383.64	383.64	8.18	.005	.050
Group x Time	1	481.86	481.86	10.27	.002	.062
Gender x Group x Time	1	205.15	205.15	4.37	.038	.027
Error	156	7316.49	46.90			

Table S6.
Group (Geospatial, Control) by Time (T1, T2) ANOVA results for SHOMI, covarying Gender, GPA, and PSAT**.**

EFT						
	Accuracy (#correct/total)		Reaction Time (seconds)		Rate Correct Score (trials correct per second)	
	Average	SD	Average	SD	Average	SD
Total						
Timepoint 1	.707	.121	5.59	.992	.120	.039
Timepoint 2	.728	.124	4.98	1.036	.147	.051
Geospatial						
Timepoint 1	.685	.120	5.52	1.10	.119	.039
Timepoint 2	.720	.124	4.78	1.18	.156	.058
Control						
Timepoint 1	.722	.120	5.64	.905	.121	.039
Timepoint 2	.734	.124	5.13	.895	.141	.045

Table S7.
Descriptive statistics for EFT in full sample at both timepoints, and separately for Geospatial and Control groups.

Reasoning						
	Accuracy (#correct/total)		Reaction Time (seconds)		Rate Correct Score (trials correct per second)	
	Average	SD	Average	SD	Average	SD
Total						
Timepoint 1	.819	.123	3.20	.882	.269	.110
Timepoint 2	.837	.149	2.92	.924	.315	.144
Geospatial						
Timepoint 1	.814	.116	3.27	.885	.258	.106
Timepoint 2	.837	.146	2.89	.991	.324	.154
Control						
Timepoint 1	.823	.129	3.15	.881	.277	.113
Timepoint 2	.836	.151	2.93	.877	.308	.136

Table S8.
Descriptive statistics for Reasoning in full sample at both timepoints, and separately for Geospatial and Control groups.

MRT						
	Accuracy (#correct/total)		Reaction Time (seconds)		Rate Correct Score (trials correct per second)	
	Average	SD	Average	SD	Average	SD
Total						
Timepoint 1	.774	.123	4.15	.509	.176	.048
Timepoint 2	.810	.119	3.75	.644	.173	.066
Geospatial						
Timepoint 1	.768	.118	4.18	.528	.173	.047
Timepoint 2	.792	.126	3.68	.766	.219	.080
Control						
Timepoint 1	.779	.127	4.12	.495	.178	.049
Timepoint 2	.823	.112	3.81	.536	.210	.053

Table S9.
Descriptive statistics for MRT in full sample at both timepoints, and separately for Geospatial and Control groups.

SHOMI Total Score		
	Average	SD
Total		
Timepoint 1	102.74	12.05
Timepoint 2	102.83	12.59
Geospatial		
Timepoint 1	103.14	11.32
Timepoint 2	105.94	12.55
Control		
Timepoint 1	102.44	12.61
Timepoint 2	100.48	12.17

Table S10.
Descriptive statistics for SHOMI in full sample at both timepoints, and separately for Geospatial and Control groups.

Predictor	se	β	p	Adj. R^2
ΔEFT	.157	0.288	<.001	
Gender	.016	-.047	.533	
GPA	.022	-.227	.015	
PSAT	.0004	.145	.113	
Overall Model			<.001	.087

Table S11.

Results of model regressing ΔReasoning on ΔEFT, covarying GPA, PSAT, and Gender.

Predictor	se	β	p	Adj. R^2
ΔEFT	.216	.291	<.001	
Gender	.021	-.028	.716	
GPA	.029	-.248	.008	
PSAT	.0006	.167	.067	
Overall Model			<.001	0.096

Table S12.
Results of model regressing ΔReasoning (nonspatial relation trials only) on ΔEFT, covarying GPA, PSAT, and Gender.

Predictor	se	β	p	Adj. R^2
ΔMRT	.124	.453	<.001	
Gender	.014	-.064	.368	
GPA	.020	-.201	.020	
PSAT	.0004	.163	.057	
Overall Model			<.001	.211

Table S13.

Results of model regressing ΔReasoning on ΔMRT, covarying GPA, PSAT, and Gender.

Predictor	se	β	p	Adj. R^2
ΔMRT	.162	.522	<.001	
Gender	.019	-.053	.431	
GPA	.027	-.229	.006	
PSAT	.0005	.186	.024	
Overall Model			<.001	.287

Table S14.
Results of model regressing ΔReasoning (nonspatial relation trials only) on ΔMRT, covarying GPA, PSAT, and Gender.

Predictor	se	β	p	Adj. R^2
ΔMRT	.127	.400	<.001	
ΔEFT	.152	.167	.024	
Gender	.014	-.077	.277	
GPA	.020	-.221	.010	
PSAT	.0004	.149	.078	
Overall Model			<.001	.229

Table S15.
Results of model regressing ΔReasoning on ΔMRT and ΔEFT, covarying GPA, PSAT, and Gender.

Predictor	se	β	p	Adj. R^2
ΔMRT	.168	.476	<.001	
ΔEFT	.199	.149	.034	
Gender	.019	-.061	.365	
GPA	.026	-.245	.003	
PSAT	.0005	.17	.029	
Overall Model			<.001	.301

Table S16.
Results of model regressing ΔReasoning (nonspatial relation trials only) on ΔMRT and ΔEFT, covarying GPA, PSAT, and Gender.

Predictor	se	β	p	Adj. R^2
ΔSHOMI	.0008	-.009	.909	
Gender	.017	-.014	.861	
GPA	.023	-.191	.054	
PSAT	.0004	.166	.085	
Overall Model			.341	.003

Table S17.
Results of model regressing ΔReasoning on ΔSHOMI, covarying GPA, PSAT, and Gender.

Hemisphere	Region	BA	Voxels	Z-score	*P*	X	Y	Z
Left	Middle Frontal Gyrus	45	193	3.63	.0002	-30	18	26
Right	Middle Frontal Gyrus	45	162	3.51	.0004	40	26	24
Left	Middle Frontal Gyrus	39	101	3.12	.0009	-40	36	34
Right	Inferior Parietal Lobe	40	67	3.32	.0008	58	-26	46
Left	Frontal Pole	46	65	3.65	.0003	-36	44	4
Left	Anterior Intra-Parietal Sulcus	40	46	3.42	.0006	-22	-58	56
Left	Premotor Cortex	6	44	3.57	.0004	-10	16	68

Table S18.
Clusters of longitudinal increases in activation during EFT (Search>Baseline contrast) from the Geospatial>Control interaction term of the whole-brain Group(Geospatial, Control) X Time(T1, T2) ANOVA (i.e., brain regions where Geospatial students showed increased activation from T1 to T2, relative to Controls).
Note: Coordinates are in MNI space (*100*); BA=Brodmann area. Cluster size refers to the number of voxels in the cluster. Coordinates are listed for activation peaks within each cluster, and brain regions are listed for the locations of those peaks.

Hemisphere	Region	BA	Voxels	Z-score	*P*	X	Y	Z	SpatialMap
Left	Anterior Intra-Parietal Sulcus and Superior Parietal Lobe	7	42	3.53	.0004	-16	-50	50	
Left	Anterior Intra-Parietal Sulcus	40	25	3.19	.0009	-32	-38	40	✓

Table S19.
Clusters of longitudinal increases in activation during Reasoning (Reasoning>Match contrast) from the Geospatial>Control interaction term of the whole-brain Group(Geospatial, Control) X Time(T1, T2) ANOVA (i.e., brain regions where Geospatial students showed increased activation from T1 to T2, relative to Controls).
Note: Coordinates are in MNI space (*100*); BA=Brodmann area. Cluster size refers to the number of voxels in the cluster. Coordinates are listed for activation peaks within each cluster, and brain regions are listed for the locations of those peaks. A check mark in the SpatialMap column indicates that a cluster overlapped with the Neurosynth-based SpatialMap ROI.

Hemisphere	Region	BA	Voxels	Z-score	*P*	X	Y	Z	SpatialMap
Right	Frontal Pole	10	1381	3.97	.00007	12	59	11	
Right	Secondary Somatosensory Cortex	40	456	3.48	.0005	66	-22	16	
Right	Secondary Visual Cortex	18	281	3.42	.0006	16	-88	34	
Right	Frontal Pole	47	273	3.91	.00009	32	40	16	
Right	Premotor Cortex	6	243	3.36	.0008	56	0	32	
Right	Primary Visual Cortex	17	175	3.36	.0008	1	-68	10	
Right	Frontal Pole	46	123	3.69	.0002	52	46	6	
Left	Primary Somatosensory Cortex	3	96	3.71	.0002	-64	-4	20	

Table S20.
Clusters of longitudinal increases in activation during Reasoning (Reasoning>Match contrast) from the Control>Geospatial interaction term of the whole-brain Group(Geospatial, Control) X Time(T1, T2) ANOVA (i.e., brain regions where Control students showed increased activation from T1 to T2, relative to Geospatial students).
Note: Coordinates are in MNI space (*100*); BA=Brodmann area. Cluster size refers to the number of voxels in the cluster. Coordinates are listed for activation peaks within each cluster, and brain regions are listed for the locations of those peaks. A check mark in the SpatialMap column indicates that a cluster overlapped with the Neurosynth-based SpatialMap ROI.

Hemisphere	Region	BA	Voxels	Z-score	*P*	X	Y	Z
Right	Posterior Cingulate	31	152	3.92	.00009	10	-40	46
Left	Inferior Occipital Gyrus	19	66	3.22	.0009	-12	-80	28

Table S21.
Clusters of longitudinal increases in activation during MRT (True trials with greater than 0 degrees of rotation>True trials with 0 degrees of rotation) from the Geospatial>Control interaction term of the whole-brain Group(Geospatial, Control) X Time(T1, T2) ANOVA (i.e., brain regions where Geospatial students showed increased activation for mental rotation from T1 to T2, relative to Controls).
Note: Coordinates are in MNI space (*100*); BA=Brodmann area. Cluster size refers to the number of voxels in the cluster. Coordinates are listed for activation peaks within each cluster, and brain regions are listed for the locations of those peaks.

Hemisphere	Region	BA	Voxels	Z-score	*P*	X	Y	Z
Left	Superior Frontal Gyrus	6	64	3.53	.0004	-20	20	64
Right	Fusiform Gyrus	37	61	4.34	.00001	48	-48	-1
Right	Supplementary Motor Area	8	51	3.35	.0008	34	20	46
Right	Frontal Pole	9	45	3.71	.0002	20	59	30
Left	Brainstem	-	42	3.38	.0007	-14	-18	-23

Table S22.
Clusters of longitudinal increases in activation during MRT (True trials with greater than 0 degrees of rotation>True trials with 0 degrees of rotation) from the Control>Geospatial interaction term of the whole-brain Group(Geospatial, Control) X Time(T1, T2) ANOVA (i.e., brain regions where Control students showed increased activation for mental rotation from T1 to T2, relative to Geospatial students).
Note: Coordinates are in MNI space (*100*); BA=Brodmann area. Cluster size refers to the number of voxels in the cluster. Coordinates are listed for activation peaks within each cluster, and brain regions are listed for the locations of those peaks.

Hemisphere	Region	BA	Voxels	Z-score	*P*	X	Y	Z	SpatialMap
Right	Thalamus	-	3277	3.72	.0002	4	-12	-4	
Right	Premotor Cortex	6	1573	4.08	.00004	4	14	52	✓
Right	Posterior Parietal Cortex	7	835	3.38	.0007	1	-70	40	✓
Left	Pallidum	-	733	3.42	.0006	-26	-10	-4	
Right	Posterior Parietal Cortex	7	458	3.40	.0007	32	-46	60	✓
Left	Anterior Intra-Parietal Sulcus	7	165	3.19	.0009	-26	-62	48	✓
Left	Premotor Cortex	6	138	3.22	.0008	-44	4	52	✓

Table S23.
Clusters of longitudinal increases in connectivity from regions within the SpatialMap ROI to the left DLPFC seed region during Reasoning (Reasoning>Match contrast) from the Geospatial>Control interaction term of the whole-brain Group(Geospatial, Control) X Time(T1, T2) ANOVA in the PPI analysis (i.e. brain regions where Geospatial students showed increased connectivity of SpatialMap to PFC from T1 to T2, relative to Controls).
Note: Coordinates are in MNI space (*100*); BA=Brodmann area. Cluster size refers to the number of voxels in the cluster. Coordinates are listed for activation peaks within each cluster, and brain regions are listed for the locations of those peaks. A check mark in the SpatialMap column indicates that a cluster overlapped with the Neurosynth-based SpatialMap ROI.

Hemisphere	Region	BA	Voxels	Z-score	*P*	X	Y	Z	SpatialMap
Bilateral	Superior Parietal Lobe	7	2378	4.23	.00002	1	-56	58	✓

Table S24.
Clusters of longitudinal increases in connectivity from regions within the SpatialMap ROI to the a prior left DLPFC seed region during Reasoning (Reasoning>Match contrast) for Female Geospatial students in the Geospatial>Control interaction term of the whole-brain Group(Geospatial, Control) X Time(T1, T2) ANOVA in the PPI analysis (i.e. brain regions where female Geospatial students showed greater increased connectivity of SpatialMap to PFC than male Geospatial students relative to Controls from T1 to T2).
Note: Coordinates are in MNI space (*100*); BA=Brodmann area. Cluster size refers to the number of voxels in the cluster. Coordinates are listed for activation peaks within each cluster, and brain regions are listed for the locations of those peaks. A check mark in the SpatialMap column indicates that a cluster overlapped with the Neurosynth-based SpatialMap ROI.

Predictor	*df*	Sum of Squares	Mean Square	*F*	*p*	np^2
(Intercept)	1	127.2	127.2	194.57	<.001	.761
Group	1	.638	.638	.975	.327	.016
Time	1	3.96	3.96	12.52	.001	.170
Group x Time	1	1.49	1.49	4.71	.034	.072
Error	61	39.88	.654			

Table S25.
Group (Geospatial, Control) by Time (T1, T2) ANOVA results for RSA-based change in similarity between nonspatial relation Reasoning trials and spatial relation Reasoning trials in aIPS.

Predictor	se	β	p	Adj. R^2
ΔaIPS_EFT	.007	.486	<.001	
Gender	.013	-.024	.840	
GPA	.021	0	.998	
PSAT	.0004	.107	.445	
Overall Model			.002	.195

Table S26.
Results of model regressing ΔEFT on ΔaIPS_EFT, covarying GPA, PSAT, and Gender.

Predictor	se	β	p	Adj. R^2
ΔIPL_EFT	.0062	.424	.001	
Gender	.014	-.065	.604	
GPA	.021	.046	.747	
PSAT	.0004	.102	.474	
Overall Model			.01	.134

Table S27.
Results of model regressing ΔEFT on ΔIPL_EFT, covarying GPA, PSAT, and Gender.

Predictor	se	β	p	Adj. R^2
ΔaIPS_EFT	.009	.492	<.001	
Gender	.019	-.031	.783	
GPA	.031	-.052	.698	
PSAT	.0005	.311	.020	
Overall Model			<.001	.271

Table S28.
Results of model regressing ΔReasoning on ΔaIPS_EFT, covarying GPA, PSAT, and Gender.

Predictor	se	β	p	Adj. R^2
ΔIPL_EFT	9.45E-05	.581	<.001	
Gender	1.81E-05	-.101	.340	
GPA	2.86E-05	-.026	.831	
PSAT	4.87E-07	.322	.011	
Overall Model			<.001	.366

Table S29.
Results of model regressing ΔReasoning on ΔIPL_EFT, covarying GPA, PSAT, and Gender.

Predictor	se	β	p	Adj. R^2
ΔSpatialMap-to-DLPFC Connectivity	.007	.309	.016	
Gender	.021	-.061	.634	
GPA	.033	.066	.647	
PSAT	.0006	.239	.099	
Overall Model			.025	.119

Table S30.
Results of model regressing ΔReasoning on ΔSpatialMap-to-DLPFC Connectivity during Reasoning, covarying GPA, PSAT, and Gender.

Predictor	se	β	p	Adj. R^2
ΔaIPS_Reasoning	.009	.152	.238	
Gender	.022	.022	.863	
GPA	.035	.068	.698	
PSAT	.0006	.246	.105	
Overall Model			.161	.045

Table S31.
Results of model regressing ΔReasoning on ΔaIPS_Reasoning, covarying GPA, PSAT, and Gender.

Predictor	se	β	p	Adj. R^2
ΔRSA_aIPS_Reasoning	.013	.242	.062	
Gender	.022	.057	.660	
GPA	.034	.058	.691	
PSAT	.0006	.271	.068	
Overall Model			.069	.081

Table S32.
Results of model regressing ΔReasoning on ΔRSA_aIPS_Reasoning (RSA-based change in nonspatial-to-spatial similarity between nonspatial and spatial relation Reasoning trials in aIPS), covarying GPA, PSAT, and Gender.

Predictor	Predictor Importance
ΔIPL_EFT	38%
ΔaIPS_EFT	28%
ΔEFT	16%
PSAT	9%
ΔMRT	4%
GPA	4%
ΔSHOMI	3%
Gender	<1%

Table S33.
Contribution of each predictor (% of total predictor "importance") to predictive modeling of ΔReasoning across the ensemble model identified via boosting (to maximize model accuracy), with both neural change predictors (ΔaIPS_EFT and ΔIPL_EFT) included in the analysis in the full neuroimaging sample.

Predictor	Predictor Importance
ΔIPL_EFT	47%
PSAT	44%
ΔaIPS_EFT	10%

Table S34.
Contribution of each predictor (% of total predictor "importance") to predictive modeling of ΔReasoning across the ensemble model identified via standard model creation (to select variables that produce the most informative standard model), with both neural change predictors (ΔaIPS_EFT and ΔIPL_EFT) included in the analysis in the full neuroimaging sample.

Predictor	Predictor Importance
ΔaIPS_EFT	26%
ΔIPL_EFT	24%
ΔEFT	18%
PSAT	16%
GPA	12%
ΔMRT	3%
ΔSHOMI	1%
Geospatial Course Grade	<1%
Gender	<1%

Table S35.
Contribution of each predictor (% of total predictor "importance") to predictive modeling of ΔReasoning across the ensemble model identified via boosting (to maximize model accuracy), with both neural change predictors (ΔaIPS_EFT and ΔIPL_EFT) included in the analysis in the analysis in the Geospatial neuroimaging sample.

Predictor	Predictor Importance
ΔaIPS_EFT	58%
PSAT	42%

Table S36.
Contribution of each predictor (% of total predictor "importance") to predictive modeling of ΔReasoning across the ensemble model identified via standard model creation (to select variables that produce the most informative standard model), with both neural change predictors (ΔaIPS_EFT and ΔIPL_EFT) included in the analysis in the Geospatial neuroimaging sample.

Predictor	Predictor Importance
ΔaIPS_EFT	51%
ΔEFT	22%
PSAT	12%
GPA	6%
ΔSHOMI	4%
ΔMRT	4%
Gender	<1%

Table S37.
Predictor contributions ("importance") to accuracy in predictive modeling of ΔReasoning (ensemble model boosting) for model including ΔaIPS_EFT as the only neural change variable in the full neuroimaging sample.

Predictor	Predictor Importance
ΔaIPS_EFT	72%
PSAT	28%

Table S38.

Contribution of each predictor (% of total predictor "importance") to predictive modeling of ΔReasoning across the ensemble model identified via standard model creation (to select variables that produce the most informative standard model), with ΔaIPS_EFT as the only neural change variable in the full neuroimaging sample.

Predictor	Predictor Importance
ΔaIPS_EFT	33%
PSAT	21%
GPA	21%
ΔEFT	20%
ΔMRT	4%
ΔSHOMI	1%
Geospatial Course Grade	<1%
Gender	<1%

Table S39.
Predictor contributions ("importance") to accuracy in predictive modeling of ΔReasoning (ensemble model boosting) for model including ΔaIPS_EFT as the neural change variable in the Geospatial neuroimaging sample.

Predictor	Predictor Importance
ΔaIPS_EFT	58%
PSAT	42%

Table S40.
Contribution of each predictor (% of total predictor "importance") to predictive modeling of ΔReasoning across the ensemble model identified via standard model creation (to select variables that produce the most informative standard model), with ΔaIPS_EFT as the only neural change variable in the Geospatial neuroimaging sample.

Predictor	Predictor Importance
ΔIPL_EFT	56%
ΔEFT	19%
PSAT	12%
ΔMRT	5%
GPA	5%
ΔSHOMI	3%
Gender	<1%

Table S41.
Predictor contributions (“importance”) to accuracy in predictive modeling of ΔReasoning (ensemble model boosting) for model including ΔIPL_EFT as the only neural change variable in the full neuroimaging sample.

Predictor	Predictor Importance
ΔIPL_EFT	75%
PSAT	25%

Table S42.
Contribution of each predictor (% of total predictor "importance") to predictive modeling of ΔReasoning across the ensemble model identified via standard model creation (to select variables that produce the most informative standard model), with ΔIPL_EFT as the only neural change variable in the full neuroimaging sample.

Predictor	Predictor Importance
ΔIPL_EFT	35%
ΔEFT	26%
PSAT	22%
GPA	14%
ΔMRT	2%
ΔSHOMI	1%
Gender	<1%
Geospatial Course Grade	<1%

Table S43.
Predictor contributions ("importance") to accuracy in predictive modeling of ΔReasoning (ensemble model boosting) for model including ΔIPL_EFT as the only neural change variable in the Geospatial neuroimaging sample.

Predictor	Predictor Importance
ΔIPL_EFT	59%
PSAT	41%

Table S44.
Contribution of each predictor (% of total predictor "importance") to predictive modeling of ΔReasoning across the ensemble model identified via standard model creation (to select variables that produce the most informative standard model), with ΔIPL_EFT as the only neural change variable in the Geospatial neuroimaging sample.

Behavioral Regression Model					Neural Regression Model				
Predictor	se	*β*	*p*	Adj. R^2	Predictor	se	*β*	*p*	Adj. R^2
ΔEFT	.191	.318	.011		ΔaIPS_EFT	.012	.232	.106	
PSAT	.0004	.251	.037		ΔIPL_EFT	.011	.290	.008	
ΔMRT	.258	.167	.173						
Overall Model			.002	.192	Overall Model			<.001	.299
Likelihood-ratio Test of Difference in Model Fit									
				χ^2	*p*				
				7.47	.006				

Table S45.
Comparison of the performance-based assessment model that achieved the best fit for ΔReasoning in the full neuroimaging sample vs. the neural change-based model including ΔaIPS_EFT and ΔIPL_EFT. The difference in model fit was significance tested via a likelihood-ratio test.

Behavioral Regression Model					Neural Regression Model				
Predictor	se	β	*p*	Adj. R^2	Predictor	se	β	*p*	Adj. R^2
ΔEFT	.249	.367	.054		ΔaIPS_EFT	.017	.353	.088	
PSAT	.0007	.240	.211		ΔIPL_EFT	.016	.210	.300	
Geospatial Course Grades	.047	-.04	.816						
Overall Model			.153	.089	Overall Model			.025	.188
Likelihood-ratio Test of Difference in Model Fit									
				χ^2	*p*				
				2.20	<.001				

Table S46.
Comparison of the performance-based assessment model that achieved the best fit for ΔReasoning in the Geospatial neuroimaging sample vs. the neural change-based model including ΔaIPS_EFT and ΔIPL_EFT.

Behavioral Regression Model					Behavioral + Neural Regression Model				
Predictor	se	*β*	*p*	Adj. R^2	Predictor	se	*β*	*p*	Adj. R^2
ΔEFT	.191	.318	.011		ΔEFT	.200	.138	.284	
PSAT	.0004	.251	.037		PSAT	.0004	.269	.017	
ΔMRT	.258	.167	.173		ΔMRT	.242	.128	.266	
					ΔaIPS_EFT	.011	.389	.003	
Overall Model			.002	.192	Overall Model			<.001	.299
Likelihood-ratio Test of Difference in Model Fit									
				χ^2	*p*				
				9.61	0.002				

Table S47.
Comparison of the performance-based assessment model that achieved the best fit for ΔReasoning in the full neuroimaging sample vs. the same regression model with the ΔaIPS_EFT neural change variable added. The difference in model fit was significance tested via a likelihood-ratio test.

Behavioral Regression Model					Behavioral + Neural Regression Model				
Predictor	se	*β*	*p*	Adj. R^2	Predictor	se	*β*	*p*	Adj. R^2
ΔEFT	.191	.318	.011		ΔEFT	.181	.124	.284	
PSAT	.0004	.251	.037		PSAT	.0004	.305	.005	
ΔMRT	.258	.167	.173		ΔMRT	.225	.134	.212	
					ΔIPL_EFT	.009	.489	<.001	
Overall Model			.002	.192	Overall Model			<.001	.387

Likelihood-ratio Test of Difference in Model Fit

χ^2	*p*
17.696	<.001

Table S48.

Comparison of the performance-based assessment model that achieved the best fit for ΔReasoning in the full neuroimaging sample vs. the same regression model with the ΔIPL_EFT neural change variable added.

Behavioral Regression Model					Behavioral + Neural Regression Model				
Predictor	se	*β*	*p*	Adj. R^2	Predictor	se	*β*	*p*	Adj. R^2
ΔEFT	.191	.318	.011		ΔEFT	.189	0.09	.483	
PSAT	.0004	.251	.037		PSAT	.0004	0.31	.005	
ΔMRT	.258	.167	.173		ΔMRT	.226	0.13	.249	
					ΔaIPS_EFT	.012	0.15	.276	
					ΔIPL_EFT	.011	0.41	.004	
Overall Model			.002	.192	Overall Model			<.001	.390

Likelihood-ratio Test of Difference in Model Fit	
χ^2	*p*
19.03	<.001

Table S49.
Comparison of the performance-based assessment model that achieved the best fit for ΔReasoning in the full neuroimaging sample vs. the same regression model with the ΔaIPS_EFT and ΔIPL_EFT neural change variables added. The difference in model fit was significance tested via a likelihood-ratio test.

Behavioral Regression Model					Behavioral + Neural Regression Model				
Predictor	se	*β*	*p*	Adj. R^2	Predictor	se	*β*	*p*	Adj. R^2
ΔEFT	.249	.367	.054		ΔEFT	.275	.132	0513	
PSAT	.0007	.238	.211		PSAT	.0007	.297	.101	
Geospatial Course Grades	.047	-.043	.816		Geospatial Course Grades	.044	-.051	.769	
					ΔaIPS_EFT	.018	.445	.037	
Overall Model			.153	.089	Overall Model			.044	.212

Likelihood-ratio Test of Difference in Model Fit

χ^2	*p*
5.35	.021

Table S50.

Comparison of the performance-based assessment model that achieved the best fit for ΔReasoning in the Geospatial neuroimaging sample vs. the same regression model with the ΔaIPS_EFT neural change variable added. The difference in model fit was significance tested via a likelihood-ratio test.

Behavioral Regression Model					Behavioral + Neural Regression Model				
Predictor	se	*β*	*p*	Adj. R^2	Predictor	se	*β*	*p*	Adj. R^2
ΔEFT	.249	.367	.054		ΔEFT	.252	.273	.149	
PSAT	.0007	.238	.211		PSAT	.0007	.234	.201	
Geospatial Course Grades	.047	-.043	.816		Geospatial Course Grades	.046	-.043	.813	
					ΔIPL_EFT	.014	.313	.098	
Overall Model			.153	.089	Overall Model			.089	.156

Likelihood-ratio Test of Difference in Model Fit

χ^2	*p*
3.37	.066

Table S51.

Comparison of the performance-based assessment model that achieved the best fit for ΔReasoning in the Geospatial neuroimaging sample vs. the same regression model with the ΔIPL_EFT neural change variable added. The difference in model fit was significance tested via a likelihood-ratio test.

Behavioral Regression Model					Behavioral + Neural Regression Model				
Predictor	se	β	p	Adj. R^2	Predictor	se	β	p	Adj. R^2
ΔEFT	.249	.367	.054		ΔEFT	.276	.127	.531	
PSAT	.0007	.238	.211		PSAT	.0007	.284	.121	
Geospatial Course Grades	.047	-.043	.816		Geospatial Course Grades	.044	-.049	.779	
					ΔaIPS_EFT	.019	.355	.132	
					ΔIPL_EFT	.016	.173	.293	
Overall Model			.153	.089	Overall Model			.066	.204

Likelihood-ratio Test of Difference in Model Fit

χ^2	p
6.29	.046

Table S52.

Comparison of the performance-based assessment model that achieved the best fit for ΔReasoning in the Geospatial neuroimaging sample vs. the same regression model with the ΔaIPS_EFT and ΔIPL_EFT neural change variables added. The difference in model fit was significance tested via a likelihood-ratio test.

Predictor	se	β	p	Adj. R^2
ΔEFT	.189	.085	.483	
PSAT	.0004	.303	.005	
ΔMRT	.226	.123	.249	
ΔaIPS_EFT	.012	.155	.276	
ΔIPL_EFT	.011	.407	.004	
Overall Model			<.001	0.39

Table S53.

Results of best-fitting model for predicting ΔReasoning combining performance-based assessments and neural changes in the full neuroimaging sample. This neural change *and* performance-based assessment-based model was the best-fitting model overall for this sample. P-value shown for ΔIPL_EFT and overall model fit is after correction for multiple comparisons.

Predictor	se	β	p	Adj. R^2
ΔEFT	.275	.132	.513	
PSAT	.0007	.297	.101	
Geospatial Course Grades	.044	-.051	.769	
ΔaIPS_EFT	.018	.445	.037	
Overall Model			.044	.212

Table S54.
Results of best-fitting model for predicting ΔReasoning combining performance-based assessments and neural changes in the Geospatial neuroimaging sample. This neural change *and* performance-based assessment-based model was the best-fitting model overall for this sample.

Predictor	se	β	p	Adj. R^2
ΔaIPS_EFT	.179	.414	<.001	
Overall Model			<.001	.158

Table S55.
Results of model regressing change in SpatialMap-to-DLPFC Connectivity on ΔaIPS_EFT.

Predictor	se	β	p	Adj. R^2
ΔIPL_EFT	.190	.262	.038	
Overall Model			.038	.053

Table S56.
Results of model regressing change in SpatialMap-to-DLPFC Connectivity on ΔIPL_EFT.

Predictor	se	β	p	Adj. R^2
ΔaIPS_EFT	.150	.340	.006	
Overall Model			.006	.101

Table S57.
Results of model regressing ΔaIPS_Reasoning on ΔaIPS_EFT.

REFERENCES AND NOTES

1. J. D. E. Gabrieli, The promise of educational neuroscience: Comment on bowers (2016). *Psychol. Rev.* **123**, 613–619 (2016).

2. D. T. Willingham, J. W. Lloyd, How educational theories can use neuroscientific data. *Mind Brain Educ.* **1**, 140–149 (2007).

3. S. Dehaene, *How We Learn: Why Brains Learn Better Than Any Machine...For Now* (Penguin, 2021).

4. S. M. Barnett, S. J. Ceci, When and where do we apply what we learn?: A taxonomy for far transfer. *Psychol. Bull.* **128**, 612–637 (2002).

5. D. L. Cusumano, Is it working?: An overview of curriculum based measurement and its uses for assessing instructional, intervention, or program effectiveness. *Behav. Anal. Today*. **8**, 24–34 (2007).

6. J. M. Voogt, J. M. Pieters, A. Handelzalts, Teacher collaboration in curriculum design teams: Effects, mechanisms, and conditions. *Educ. Res. Eval.* **22**, 121–140 (2016).

7. B. Garelick, Miracle math: A successful program from singapore tests the limits of school reform in the suburbs. *Educ. Next* **6**, 38–45 (2006).

8. P. N. Johnson-Laird, Mental models and human reasoning. *Proc. Natl. Acad. Sci. U.S.A.* **107**, 18243–18250 (2010).

9. I. Kant, *Critique of Pure Reason* (1781).

10. M. Knauff, A neuro-cognitive theory of deductive relational reasoning with mental models and visual images. *Spat. Cogn. Comput.* **9**, 109–137 (2009).

11. P. N. Johnson-laird, P. N. Johnson-laird, Mental models, deductive reasoning and the brain, in *The Cognitive Neurosciences* (MIT Press, 1995), pp. 999–1008.

12. J. Prado, A. Chadha, J. R. Booth, The brain network for deductive reasoning: A quantitative meta-analysis of 28 neuroimaging studies. *J. Cogn. Neurosci.* **23**, 3483–3497 (2011).

13. C. Wendelken, E. Ferrer, K. J. Whitaker, S. A. Bunge, Fronto-parietal network reconfiguration supports the development of reasoning ability. *Cereb. Cortex* **26**, 2178–2190 (2016).

14. A. P. Mackey, A. T. Miller Singley, S. A. Bunge, Intensive reasoning training alters patterns of brain connectivity at rest. *J. Neurosci.* **33**, 4796–4803 (2013).

15. R. E. Jung, R. J. Haier, The parieto-frontal integration theory (P-FIT) of intelligence: Converging neuroimaging evidence. *Behav. Brain Sci.* **30**, 135–154 (2007).

16. V. Goel, R. J. Dolan, Functional neuroanatomy of three-term relational reasoning. *Neuropsychologia* **39**, 901–909 (2001).

17. National Research Council, *Learning to Think Spatially.* (National Academies Press, 2006).

18. N. S. Newcombe, "Harnessing spatial thinking to support stem learning" (Organisation for Economic Cooperation and Development, 2017) pp. 51.

19. J. Wai, D. Lubinski, C. P. Benbow, Spatial ability for STEM domains: Aligning over 50 years of cumulative psychological knowledge solidifies its importance. *J. Educ. Psychol.* **101**, 817–835 (2009).

20. D. H. Uttal, D. I. Miller, N. S. Newcombe, Exploring and enhancing spatial thinking: Links to achievement in science, technology, engineering, and mathematics? *Curr. Dir. Psychol. Sci.* **22**, 367–373 (2013).

21. J. Wai, D. H. Uttal, Why spatial reasoning matters for education policy. *Am. Enterp. Inst.*, 8 (2018).

22. S. Dehejia, R. H. Wahba, Propensity score-matching methods for nonexperimental causal studies. *Rev. Econ. Stat.* **84**, 151–161 (2002).

23. S. O. Becker, A. Ichino, Estimation of average treatment effects based on propensity scores. *Stata J. Promot. Commun. Stat. Stata*. **2**, 358–377 (2002).

24. B. Kolvoord, K. Keranen, S. Rittenhouse, The geospatial semester: Concurrent enrollment in geospatial technologies. *J. Geog.* **118**, 3–10 (2019).

25. H. A. Witkin, Individual differences in ease of perception of embedded figures. *J. Pers.* **19**, 1–15 (1950).

26. R. Shepard, J. Metzler, Mental rotation of three-dimensional objects. *Science* **171**, 701–703 (1971).

27. H. R. Liesefeld, M. Janczyk, Combining speed and accuracy to control for speed-accuracy trade-offs(?). *Behav. Res. Methods* **51**, 40–60 (2019).

28. M. Kim, R. Bednarz, Effects of a GIS course on self-assessment of spatial habits of mind (SHOM). *J. Geog.* **112**, 165–177 (2013).

29. M. Caliendo, S. Kopeinig, Some practical guidance for the implementation of propensity score matching. *J. Econ. Surv.* **22**, 31–72 (2008).

30. D. Voyer, S. Voyer, M. P. Bryden, Magnitude of sex differences in spatial abilities: A meta-analysis and consideration of critical variables. *Psychol. Bull.* **117**, 250–270 (1995).

31. C. C. Ruff, M. Knauff, T. Fangmeier, J. Spreer, Reasoning and working memory: Common and distinct neuronal processes. *Neuropsychologia* **41**, 1241–1253 (2003).

32. P. N. J.-Laird, Mental models and deduction. *Trends Cogn. Sci.* **5**, 434–442 (2001).

33. E. Walter, P. Dassonville, Activation in a frontoparietal cortical network underlies individual differences in the performance of an embedded figures task. *PLOS ONE* **6**, e20742 (2011).

34. M. Peters, C. Battista, Applications of mental rotation figures of the Shepard and Metzler type and description of a mental rotation stimulus library. *Brain Cogn.* **66**, 260–264 (2008).

35. S. P. Wright, Adjusted P-values for simultaneous inference. *Biometrics* **48**, 1005–1013 (1992).

36. M. Miočević, H. P. O'Rourke, D. P. MacKinnon, H. C. Brown, Statistical properties of four effect-size measures for mediation models. *Behav. Res. Methods* **50**, 285–301 (2018).

37. J. Cohen, *Statistical power analysis for the behavioral sciences* (Academic Press, Burlington, Elsevier Science, 2013) pp. 459.

38. A. J. Fairchild, D. P. MacKinnon, M. P. Taborga, A. B. Taylor, R^2 effect-size measures for mediation analysis. *Behav. Res. Methods* **41**, 486–498 (2009).

39. T. Yarkoni, R. A. Poldrack, T. E. Nichols, D. C. Van Essen, T. D. Wager, Large-scale automated synthesis of human functional neuroimaging data. *Nat. Methods* **8**, 665–670 (2011).

40. C. R. Gillebert, D. Mantini, V. Thijs, S. Sunaert, P. Dupont, R. Vandenberghe, Lesion evidence for the critical role of the intraparietal sulcus in spatial attention. *Brain* **134**, 1694–1709 (2011).

41. K. L. Alfred, A. C. Connolly, J. S. Cetron, D. J. M. Kraemer, Mental models use common neural spatial structure for spatial and abstract content. *Commun. Biol.* **3**, 17 (2020).

42. J. T. Coull, C. D. Frith, Differential activation of right superior parietal cortex and intraparietal sulcus by spatial and nonspatial attention. *Neuroimage* **8**, 176–187 (1998).

43. J. C. Lynch, V. B. Mountcastle, W. H. Talbot, T. C. Yin, Parietal lobe mechanisms for directed visual attention. *J. Neurophysiol.* **40**, 362–389 (1977).

44. C. D. Chambers, J. M. Payne, M. G. Stokes, J. B. Mattingley, Fast and slow parietal pathways mediate spatial attention. *Nat. Neurosci.* **7**, 217–218 (2004).

45. J. M. Zacks, Neuroimaging studies of mental rotation: A meta-analysis and review. *J. Cogn. Neurosci.* **20**, 1–19 (2008).

46. M. Piazza, V. Izard, P. Pinel, D. Le Bihan, S. Dehaene, Tuning curves for approximate numerosity in the human intraparietal sulcus. *Neuron* **44**, 547–555 (2004).

47. H. Yang, The case for being automatic: Introducing the automatic linear modeling (LINEAR) procedure in SPSS statistics. *Mult. Linear Regres. Viewpoints* **39**, 27–37 (2013).

48. D. H. Uttal, N. G. Meadow, E. Tipton, L. L. Hand, A. R. Alden, C. Warren, N. S. Newcombe, The malleability of spatial skills: A meta-analysis of training studies. *Psychol. Bull.* **139**, 352–402 (2013).

49. Z. Hawes, J. Moss, B. Caswell, S. Naqvi, S. MacKinnon, Enhancing Children's spatial and numerical skills through a dynamic spatial approach to early geometry instruction: Effects of a 32-week intervention. *Cogn. Instr.* **35**, 236–264 (2017).

50. T. Lowrie, T. Logan, M. Hegarty, The influence of spatial visualization training on students' spatial reasoning and mathematics performance. *J. Cogn. Dev.* **20**, 729–751 (2019).

51. E. M. Berger, E. Fehr, H. Hermes, D. Schunk, K. Winkel, The impact of working memory training on children's cognitive and noncognitive skills. *SSRN Electron. J.*, 1–78 (2020).

52. S. Bergman-Nutley, T. Klingberg, Effect of working memory training on working memory, arithmetic and following instructions. *Psychol. Res.* **78**, 869–877 (2014).

53. L. S. Liben, The STEM gender gap: The case for spatial interventions. *Int. J. Gender Sci. Technol.* **7**, 133–150 (2015).

54. P. A. Alexander, S. Jablansky, L. M. Singer, D. Dumas, Relational reasoning: What we know and why it matters. *Brain Sci.* **3**, 36–44 (2016).

55. S. A. Bunge, E. R. Leib, How does education hone reasoning ability? *Curr. Dir. Psychol. Sci.* **29**, 167–173 (2020).

56. M. Meshulam, L. Hasenfratz, H. Hillman, Y.-F. Liu, M. Nguyen, K. A. Norman, U. Hasson, Neural alignment predicts learning outcomes in students taking an introduction to computer science course. *Nat. Commun.* **12**, 1922 (2021).

57. S. Dehaene, M. Kerszberg, J.-P. Changeux, A neuronal model of a global workspace in effortful cognitive tasks. *Proc. Natl. Acad. Sci. U.S.A.* **95**, 14529–14534 (1998).

58. B. Dunst, M. Benedek, E. Jauk, S. Bergner, K. Koschutnig, M. Sommer, A. Ischebeck, B. Spinath, M. Arendasy, M. Bühner, H. Freudenthaler, A. C. Neubauer, Neural efficiency as a function of task demands. *Dermatol. Int.* **42**, 22–30 (2014).

59. D. M. Barch, T. S. Braver, L. E. Nystrom, S. D. Forman, D. C. Noll, J. D. Cohen, Dissociating working memory from task difficulty in human prefrontal cortex. *Neuropsychologia* **35**, 1373–1380 (1997).

60. T. Yarkoni, D. M. Barch, J. R. Gray, T. E. Conturo, T. S. Braver, BOLD correlates of trial-by-trial reaction time variability in gray and white matter: A multi-study fMRI analysis. *PLOS ONE* **4**, e4257 (2009).

61. N. Judd, T. Klingberg, Training spatial cognition enhances mathematical learning in a randomized study of 17,000 children. *Nat. Hum. Behav.* **5**, 1548–1554 (2021).

62. A. L. Gentner, D. Stevens, *Mental Models* (Psychology Press, 2014).

63. J. S. Cetron, A. C. Connolly, S. G. Diamond, V. V. May, J. V. Haxby, D. J. M. Kraemer, Using the force: STEM knowledge and experience construct shared neural representations of engineering concepts. *npj Sci. Learn.* **5**, 6 (2020).

64. J. S. Cetron, A. C. Connolly, S. G. Diamond, V. V. May, J. V. Haxby, D. J. M. Kraemer, Decoding individual differences in STEM learning from functional MRI data. *Nat. Commun.* **10**, 2027 (2019).

65. A. Vandierendonck, A comparison of methods to combine speed and accuracy measures of performance: A rejoinder on the binning procedure. *Behav. Res. Methods* **49**, 653–673 (2017).

66. S. R. Damarla, T. A. Keller, R. K. Kana, V. L. Cherkassky, D. L. Williams, N. J. Minshew, M. A. Just, Cortical underconnectivity coupled with preserved visuospatial cognition in autism: Evidence from an fMRI study of an embedded figures task. *Autism Res.* **3**, 273–279 (2010).

67. H. A. Ring, S. Baron-Cohen, S. Wheelwright, S. C. R. Williams, M. Brammer, C. Andrew, E. T. Bullmore, Cerebral correlates of preserved cognitive skills in autism. *Brain* **122**, 1305–1315 (1999).

68. D. Voyer, J. Hou, Type of items and the magnitude of gender differences on the mental rotations test. *Can. J. Exp. Psychol.* **60**, 91–100 (2006).

69. J. Verzani, *Using R for Introductory Statistics* (Chapman and Hall/CRC, 2018).

70. I. Corp, IBM SPSS Statistics for Windows, version 27.0 (2020).

71. C. J. M. Maas, J. J. Hox, Sufficient sample sizes for multilevel modeling. *Methodology* **1**, 86–92 (2005).

72. O. Paccagnella, Sample size and accuracy of estimates in multilevel models. *Methodology* **7**, 111–120 (2011).

73. J. A. Schoeneberger, The impact of sample size and other factors when estimating multilevel logistic models. *J. Exp. Educ.* **84**, 373–397 (2016).

74. N. Sommet, D. Morselli, Keep calm and learn multilevel logistic modeling: A simplified three-step procedure using stata, R, Mplus, and SPSS. *Int. Rev. Soc. Psychol.* **30**, 203–218 (2017).

75. S. M. Smith, Fast robust automated brain extraction. *Hum. Brain Mapp.* **17**, 143–155 (2002).

76. M. Jenkinson, P. Bannister, M. Brady, S. Smith, Improved optimization for the robust and accurate linear registration and motion correction of brain images. *Neuroimage* **17**, 825–841 (2002).

77. M. Jenkinson, S. Smith, A global optimisation method for robust affine registration of brain images. *Med. Image Anal.* **5**, 143–156 (2001).

78. M. W. Woolrich, B. D. Ripley, M. Brady, S. M. Smith, Temporal autocorrelation in univariate linear modeling of FMRI data. *Neuroimage* **14**, 1370–1386 (2001).

79. C. F. Beckmann, M. Jenkinson, S. M. Smith, General multilevel linear modeling for group analysis in FMRI. *Neuroimage* **20**, 1052–1063 (2003).

80. A. Eklund, T. E. Nichols, H. Knutsson, Cluster failure: Why fMRI inferences for spatial extent have inflated false-positive rates. *Proc. Natl. Acad. Sci. U.S.A.* **113**, 7900–7905 (2016).

81. K. Friston, C. Buechel, G. Fink, J. Morris, E. Rolls, R. Dolan, Psychophysiological and modulatory interactions in neuroimaging. *Neuroimage* **6**, 218–229 (1997).

82. J. X. O'Reilly, M. W. Woolrich, T. E. J. Behrens, S. M. Smith, H. Johansen-Berg, Tools of the trade: Psychophysiological interactions and functional connectivity. *Soc. Cogn. Affect. Neurosci.* **7**, 604–609 (2012).

83. P. A. Kriegeskorte, N. Mur, M. Bandettini, Representational similarity analysis-connecting the branches of systems neuroscience. *Front. Syst. Neurosci.* **2**, 4 (2008).

84. I. M. Lyons, D. Ansari, S. L. Beilock, Qualitatively different coding of symbolic and nonsymbolic numbers in the human brain. *Hum. Brain Mapp.* **36**, 475–488 (2015).

85. M. Knauff, P. N. Johnson-Laird, Visual imagery can impede reasoning. *Mem. Cognit.* **30**, 363–371 (2002).

86. R. Shepard, C. Feng, A chronometric study of mental paper folding. *Cogn. Psychol.* **3**, 228–243 (1972).

87. R. A. Doyle, D. Voyer, I. D. Cherney, The relation between childhood spatial activities and spatial abilities in adulthood. *J. Appl. Dev. Psychol.* **33**, 112–120 (2012).

88. G. M. Furnival, R. W. Wilson, Regressions by leaps and bounds. *Dent. Tech.* **16**, 499–511 (1974).

89. E. A. Stuart, H. A. Huskamp, K. Duckworth, J. Simmons, Z. Song, M. E. Chernew, C. L. Barry, Using propensity scores in difference-in-differences models to estimate the effects of a policy change. *Heal. Serv. Outcomes Res. Methodol.* **14**, 166–182 (2014).

90. P. N. Johnson-Laird, The history of mental models, in *Psychology of Reasoning* (Psychology Press, 2004), pp. 189–222.

91. C. C. Richardson, A. Gonzalez, L. Leal, M. Z. Castillo, C. A. Carman, PSAT component scores as a predictor of success on AP exam performance for diverse students. *Educ. Urban Soc.* **48**, 384–402 (2016).

92. D. J. Woltz, C. A. Was, Availability of related long-term memory during and after attention focus in working memory. *Mem. Cognit.* **34**, 668–684 (2006).

93. A. Vandierendonck, Further tests of the utility of integrated speed-accuracy measures in task switching. *J. Cogn.* **1**, 8 (2018).

94. I. Gauthier, K.-W. Chua, J. J. Richler, How holistic processing of faces relates to cognitive control and intelligence. *Atten. Percept. Psychophys.* **80**, 1449–1460 (2018).

95. D. Tingley, T. Yamamoto, K. Hirose, L. Keele, K. Imai, Mediation: R package for causal mediation analysis. *J. Stat. Softw.* **59**, 1–38 (2014).

96. P. E. Shrout, N. Bolger, Mediation in experimental and nonexperimental studies: New procedures and recommendations. *Psychol. Methods* **7**, 422–445 (2002).

97. J. Wertheim, M. Ragni, The neurocognitive correlates of human reasoning: A meta-analysis of conditional and syllogistic inferences. *J. Cogn. Neurosci.* **32**, 1061–1078 (2020).

98. H. Drucker, Improving regressors using boosting techniques, in *Proceedings of the 14th International Conferences on Machine Learning* (Morgan Kaufmann Publishers Inc., 1997), pp. 107–115.

99. R. Pfister, K. Schwarz, R. Carson, M. Jancyzk, Easy methods for extracting individual regression slopes: Comparing SPSS, R, and Excel. *Tutor. Quant. Methods Psychol.* **9**, 72–78 (2013).

100. A. C. Evans, D. L. Collins, S. R. Mills, E. D. Brown, R. L. Kelly, T. M. Peters, 3D statistical neuroanatomical models from 305 MRI volumes, in *1993 IEEE Conference Record Nuclear Science Symposium and Medical Imaging Conference* (IEEE 1993), pp. 1813–1817.
